# Supplementary material for: Economic and modeling evidence for tuberculosis preventive therapy among people living with HIV: A systematic review and meta-analysis
Source: PLoS Med. 2021 Sep 14;18(9):e1003712. doi: 10.1371/journal.pmed.1003712 (PMC8439468; doi:10.1371/journal.pmed.1003712)
Supplement: S1 Text — Table A: Search strategy for MEDLINE, Embase, and Web of Science. Table B: Quality assessment checklist. Table C: Quality assessment results. Table D: Number of studies that used each type of data source for key input parameter categories. Table E: Comparing input parameters that were based on data to those that were based on assumptions. Table F: description of included studies. Table G: Key outcomes among studies that report effectiveness or utility outcomes only. Table H: Key outcomes and results of studies that reported costs and cost-effectiveness outcomes (2020 USD). Table I: Detailed outcomes of studies that reported cost and cost-effectiveness outcomes. Table J: Detailed outcomes of studies that reported effectiveness outcomes only. Table K: Data used for regression analyses. Table L: Comparing one-way sensitivity analysis results across cost and cost-effectiveness studies. Table M: Comparing one-way sensitivity analysis results across studies that only report effectiveness or utility outcomes. Table N: Threshold analysis results among included studies (that reported key thresholds where conclusions changed). Table O: Effect of 0.5× and 3× GDP per capita willingness-to-pay threshold on univariable analysis of incremental net monetary benefit. Table P: Effect of 0.5× and 3× GDP per capita willingness-to-pay threshold on multivariable analysis of incremental net monetary benefit. Table Q: Effect of 0.5× and 3× GDP per capita willingness-to-pay threshold on pooling analysis of incremental net monetary benefit. Fig A: Comparing the association between art use, TPT efficacy, and time horizon (model inputs). Fig B: Forest plot: pooling incremental net monetary benefit in LMICs. Fig C: Forest plot: pooling incremental net monetary benefit in HICs. Fig D: Model inputs: comparing time horizon by TPT regimen category and country-level income. Fig E: Model inputs: comparing TPT efficacy in preventing active TB by TPT regimen category and country-level income. Fig F. Mo [file pmed.1003712.s003.docx]

**Supplementary Material**

Economic and modelling evidence for tuberculosis preventive therapy in people living with HIV: a systematic review & meta-analysis

TABLE OF CONTENTS

[SEARCH STRATEGY 4](#_Toc75880514)

[Table A. Search Strategy for Medline, Embase and Web of Science 4](#_Toc75880515)

[QUALITY ASSESSMENT 5](#_Toc75880516)

[Table B. Quality assessment checklist 5](#_Toc75880517)

[Table C. Quality assessment results 6](#_Toc75880518)

[Data sources 9](#_Toc75880519)

[Table D. Number of studies that used each type of data source for key input parameter categories 9](#_Toc75880520)

[Input parameters and assumptions 9](#_Toc75880521)

[Table E. Comparing input parameters that were based on data to those that were based on assumptions 10](#_Toc75880522)

[DESCRIPTION OF INCLUDED STUDIES 11](#_Toc75880523)

[Table F. Description of included studies 11](#_Toc75880524)

[Table G. Key outcomes among studies that report effectiveness or utility outcomes only 16](#_Toc75880525)

[Table H. Key outcomes and results of studies that reported costs and cost-effectiveness outcomes (2020 USD) 18](#_Toc75880526)

[Table I. Detailed outcomes of studies that reported cost and cost-effectiveness outcomes 22](#_Toc75880527)

[Table J. Detailed outcomes of studies that reported effectiveness outcomes only 29](#_Toc75880528)

[REGRESSION ANALYSIS OF INPUT PARAMETERS ASSOCIATED WITH OUTCOMES 34](#_Toc75880529)

[Table K. Data used for regression analyses 34](#_Toc75880530)

[Fig A. Comparing the association between ART use, TPT efficacy and time horizon (model inputs). 39](#_Toc75880532)

[Fig B. Forest plot: pooling incremental net monetary benefit in low- and middle-income countries. 40](#_Toc75880534)

[Fig C. Forest plot: pooling incremental net monetary benefit in high-income countries. 40](#_Toc75880536)

[SUPPLEMENTAL RESULTS: SENSITIVITY AND THRESHOLD ANALYSES 41](#_Toc75880537)

[Outcomes may be sensitive to changes in ART coverage and efficacy: 41](#_Toc75880538)

[Table L. Comparing one-way sensitivity analysis results across cost and cost-effectiveness studies 42](#_Toc75880539)

[Table M. Comparing one-way sensitivity analysis results across studies that only report effectiveness or utility outcomes 50](#_Toc75880540)

[Table N. Threshold analysis results among included studies (that reported key thresholds where conclusions changed) 53](#_Toc75880541)

[Table O. Effect of 0.5x and 3x GDP per capita willingness-to-pay threshold on univariable analysis of incremental net monetary benefit 56](#_Toc75880542)

[Table P. Effect of 0.5x and 3x GDP per capita willingness-to-pay threshold on multivariable analysis of incremental net monetary benefit 57](#_Toc75880543)

[Table Q. Effect of 0.5x and 3x GDP per capita willingness-to-pay threshold on pooling analysis of incremental net monetary benefit 58](#_Toc75880544)

[SUPPLEMENTAL RESULTS: FIGURES 59](#_Toc75880545)

[Fig D. Model inputs: comparing time horizon by TPT regimen category and country-level income. 59](#_Toc75880546)

[Fig E. Model inputs: comparing TPT efficacy in preventing active TB by TPT regimen category and country-level income. 60](#_Toc75880547)

[Fig G. Model outputs: comparing per-person cost of strategies that included TPT by TPT regimen category and country-level income. 62](#_Toc75880548)

[Fig H. Model inputs: select variables and their relationship to the per-person cost of strategies that included TPT. 63](#_Toc75880549)

[Fig I. Model inputs versus model outputs: comparing calculated effectiveness based on model inputs (efficacy x adherence) to reported effectiveness based on model outputs (percent reduction in active TB incidence). 64](#_Toc75880550)

[Fig J. Model outputs: comparing reduction in active TB incidence by TPT regimen category and country-level income. 65](#_Toc75880551)

[Fig K. Model outputs: comparing incremental cost per active TB case averted by country-level income and TPT regimen category 66](#_Toc75880552)

[Outliers 67](#_Toc75880553)

[DATA EXTRACTION FORM 68](#_Toc75880554)

[REFERENCES 70](#_Toc75880555)

# SEARCH STRATEGY

## Table A. Search Strategy for Medline, Embase and Web of Science

| **INDEX** | **KEYWORD** |
| --- | --- |
| *1* | exp Tuberculosis/ |
| *2* | exp Latent Tuberculosis/ |
| *3* | (Latent TB or latent tuberculosis or tuberculosis infection or TB infection or inactive tuberculosis or LTBI or latent tuberc*).ti,ab,kw. |
| *4* | exp HIV/ |
| *5* | exp HIV Infections/ |
| *6* | exp Acquired Immunodeficiency Syndrome/ |
| *7* | (HIV or Human Immunodeficiency Virus or PLHIV or patient living with HIV or person living with HIV or people living with HIV or AIDS).ti,ab,kw. |
| *8* | exp Isoniazid/ |
| *9* | exp Rifampin/ |
| *10* | exp Rifamycins/ |
| *11* | exp Rifabutin/ |
| *12* | exp Pyrazinamide/ |
| *13* | exp Antitubercular Agents/ |
| *14* | (TPT or IPT or 3HP or 1HP or INH or RIF or RPT or PZA).ti,ab,kw. |
| *15* | (TB preventive therapy or TB preventative therapy or LTBI therapy or LTBI preventive therapy or LTBI preventative therapy).ti,ab,kw. |
| *16* | (TB or LTBI adj3(prophylaxis or chemoprophylaxis or chemoprevention)).ti, ab,kw. |
| *17* | exp Cost Benefit Analysis/ |
| *18* | exp Cost Effectiveness/ |
| *19* | exp Economic Evaluation/ |
| *20* | exp Cost-Effectiveness Analysis/ |
| *21* | exp Health Care Costs/ |
| *22* | exp Costs and Cost Analysis/ |
| *23* | exp Mathematical Model/ |
| *24* | exp Mathematical Models/ |
| *25* | (model or modelling or modelling study or dynamic model or transmission model or decision analysis model or decision analysis or CEA or CUA or cost utility analysis or cost-effective* or impact or potential impact or impact analysis or potential risks or potential benefits or econometric analysis or economic analysis).ti,ab,kw. |
| *26* | 1 OR 2 OR 3 |
| *27* | 4 OR 5 OR 6 OR 7 |
| *28* | 8 OR 9 OR 10 OR 11 OR 12 OR 13 OR 14 OR 15 OR 16 |
| *29* | 17 OR 18 OR 19 OR 20 OR 21 OR 22 OR 23 OR 24 OR 25 |
| *30* | 26 AND 27 AND 28 AND 29 |

*TB = tuberculosis, HIV = human immunodeficiency virus, PLHIV = people living with HIV, AIDS = acquired immunodeficiency syndrome, TPT = tuberculosis preventive therapy, IPT = isoniazid preventive therapy, 3HP = 3 months of isoniazid and rifapentine, 1HP = 1 month of isoniazid and rifapentine, INH = isoniazid, RIF = rifampin or rifamycin = RPT = rifapentine, PZA = pyrazinamide, LTBI = latent tuberculosis infection, CEA = cost-effectiveness analysis, CUA = cost-utility analysis*

# QUALITY ASSESSMENT

Each item in the quality assessment checklist is described below. A low-quality study was one that either (1) did not meet one of checklist items #1 and #3, or (2) met less than 7 out of 10 of the checklist items altogether. All other studies were of high quality. Criterion #1 was deemed essential because descriptions of “study arms” or “strategies” are key to being able to tease apart effects of TPT among outcomes, and criterion #3 was deemed essential because a model’s outcomes are only of high-quality if the model’s input parameters are of high quality. Table S2 on the following page lists quality assessment results of each study. A full reference list of all included studies is included at the end of this document [1-57].

## Table B. Quality assessment checklist

| **Checklist Item** | **Description** | |
| --- | --- | --- |
| **(1)** | Includes a clear description of intervention and control, or other interventions being compared | |
| **(2)** | Indicates currency used and year (cost-studies only) | |
| **(3)** | Uses a systematic review or meta-analysis to inform the following input parameters if included in the model OR if evaluating a local trial, uses local trial data (studies published before 1999 are exempted from this criteria for TPT related parameters because no reviews specific to TPT efficacy in PLHIV existed prior to that year) | i. Rate of TPT completion/adherence |
|  |  | ii. TPT efficacy in preventing active TB |
|  |  | iii. TST sensitivity |
|  |  | iv. TST specificity |
|  |  | v. IGRA sensitivity |
|  |  | vi. IGRA specificity |
| **(4)** | Data used for input parameters reflects relevant setting or population (e.g. if the study focuses on high-income countries, then the LTBI prevalence parameter shouldn’t come from a low-income country) | |
| **(5)** | Includes at least one primary data source (epidemiologic study, regional program or clinic, gov’t report, interviews with staff, program evaluation, hospital records) if the study question relates to a specific setting | |
| **(6)** | Clearly outlines key assumptions related to model structure, parameters, or analysis methods | |
| **(7)** | Uncertainty of outcomes are communicated (i.e. results have confidence intervals, or some form of probabilistic sensitivity analysis has been done) | |
| **(8)** | Explores uncertainty in input parameters through a type of sensitivity analysis (for transmission models, a careful calibration of input parameters and/or post-hoc scenario analysis is considered equivalent to a sensitivity analysis, as calibration is more common practice) | |
| **(9)** | Discusses potential limitations of analysis | |
| **(10)** | Includes a section on potential conflicts of interest | |

*TB = tuberculosis, TPT = tuberculosis preventive therapy, TST = tuberculin skin test, IGRA = interferon gamma release assay*

## Table C. Quality assessment results

| **Study** | **CHECKLIST ITEMS** | | | | | | | | | | | | | | |
| --- | --- | --- | --- | --- | --- | --- | --- | --- | --- | --- | --- | --- | --- | --- | --- |
|  | (1) | (2) | (3) | | | | | | (4) | (5) | (6) | (7) | (8) | (9) | (10) |
|  |  |  | (i) | (ii) | (iii) | (iv) | (v) | (vi) |  |  |  |  |  |  |  |
| Azadi, M. | ✔ | ✔ |  |  |  |  |  |  | ✔ | ✔ | ✔ | ✔ | ✔ | ✔ | ✔ |
| Awoke, T. D. | ✔ |  |  |  |  |  |  |  | ✔ | ✔ | ✔ |  | ✔ |  | ✔ |
| Bacaer, N. | ✔ | NA |  | ✔ |  |  |  |  | ✔ | ✔ | ✔ | ✔ | ✔ | ✔ | ✔ |
| Bachmann, M. | ✔ | ✔ |  |  |  |  |  |  | ✔ |  | ✔ |  | ✔ | ✔ | ✔ |
| Basu, S. | ✔ | NA | ✔ | ✔ |  |  |  |  | ✔ |  | ✔ | ✔ | ✔ | ✔ | ✔ |
| Bell, J. | ✔ | ✔ |  |  |  |  |  |  | ✔ | ✔ | ✔ |  | ✔ | ✔ | ✔ |
| Brewer, T. (1996) | ✔ | NA |  |  |  |  |  |  | ✔ | ✔ | ✔ | ✔ | ✔ |  | ✔ |
| Brewer, T. (2001) | ✔ | ✔ | ✔ |  |  |  |  |  | ✔ | ✔ | ✔ | ✔ | ✔ | ✔ | ✔ |
| Burgos, J. | ✔ | ✔ |  |  |  |  |  |  | ✔ | ✔ |  | ✔ | ✔ | ✔ | ✔ |
| Cohen, T. | ✔ | NA |  |  |  |  |  |  | ✔ |  | ✔ |  | ✔ |  | ✔ |
| Currie, C. | ✔ | ✔ | ✔ | ✔ |  |  |  |  | ✔ | ✔ | ✔ | ✔ | ✔ | ✔ | ✔ |
| de Siqueira Filha, N. (2018) | ✔ | ✔ |  |  |  |  |  |  | ✔ | ✔ | ✔ |  |  | ✔ | ✔ |
| de Siqueira Filha, N. (2018) | ✔ | ✔ |  |  |  |  |  |  | ✔ | ✔ | ✔ | ✔ | ✔ | ✔ | ✔ |
| Dowdy, D. | ✔ | NA |  | ✔ |  |  |  |  | ✔ | ✔ | ✔ | ✔ | ✔ | ✔ | ✔ |
| Dye, C. | ✔ | NA |  | ✔ |  |  |  |  | ✔ | ✔ | ✔ |  | ✔ |  |  |
| Ferguson, O. | ✔ | ✔ |  |  |  |  |  |  | ✔ |  | ✔ | ✔ | ✔ | ✔ | ✔ |
| Foster, S. | ✔ | ✔ |  |  |  |  |  |  | ✔ | ✔ | ✔ |  | ✔ | ✔ | ✔ |
| Freiman, J. | ✔ | NA |  |  |  |  |  |  | ✔ |  | ✔ | ✔ | ✔ | ✔ | ✔ |
| Gilbert, J. A. | ✔ | ✔ | ✔ | ✔ |  |  |  |  | ✔ | ✔ | ✔ | ✔ | ✔ | ✔ | ✔ |
| Gourevitch, M. | ✔ | ✔ |  |  |  |  |  |  | ✔ | ✔ | ✔ |  | ✔ | ✔ | ✔ |
| Gupta, S. | ✔ | ✔ |  | ✔ | ✔ |  |  |  | ✔ | ✔ | ✔ | ✔ | ✔ | ✔ | ✔ |
| Guwatudde, D. | ✔ | NA |  | ✔ |  |  |  |  | ✔ |  | ✔ |  | ✔ | ✔ | ✔ |
| Hausler, H. P. | ✔ | ✔ | ✔ | ✔ |  |  |  |  | ✔ | ✔ | ✔ |  | ✔ | ✔ | ✔ |
| Heymann, S. J. | ✔ | ✔ |  |  |  |  |  |  | ✔ |  | ✔ |  | ✔ |  |  |
| Houben, R. | ✔ | NA | ✔ |  |  |  |  |  | ✔ | ✔ | ✔ | ✔ | ✔ |  | ✔ |
| Hseih, Y. L. | ✔ | ✔ |  | ✔ |  |  |  |  | ✔ | ✔ | ✔ | ✔ | ✔ | ✔ | ✔ |
| Johnson, K. | ✔ | ✔ | ✔ | ✔ |  |  |  |  | ✔ |  | ✔ |  | ✔ | ✔ | ✔ |
| Jo, Y. | ✔ | ✔ |  | ✔ |  |  | ✔ |  | ✔ | ✔ | ✔ | ✔ | ✔ | ✔ | ✔ |
| Jordan, T. J. | ✔ | NA |  |  |  |  |  |  | ✔ |  | ✔ |  | ✔ | ✔ |  |
| Kapoor, S. | ✔ | ✔ |  |  |  |  |  |  | ✔ |  | ✔ | ✔ | ✔ | ✔ | ✔ |
| Kendall, E. | ✔ | NA | ✔ |  |  |  |  |  | ✔ | ✔ | ✔ | ✔ | ✔ | ✔ |  |
| Kim, H-Y. | ✔ | ✔ | ✔ |  |  |  | ✔ | ✔ | ✔ | ✔ | ✔ | ✔ | ✔ | ✔ | ✔ |
| Kowada, A. | ✔ | ✔ |  |  | ✔ | ✔ | ✔ | ✔ | ✔ |  | ✔ | ✔ | ✔ | ✔ | ✔ |
| Kunkel, A. | ✔ | NA |  |  |  |  |  |  | ✔ | ✔ | ✔ | ✔ | ✔ | ✔ |  |
| Linas, B. | ✔ | ✔ |  |  |  |  |  |  | ✔ | ✔ | ✔ |  | ✔ | ✔ |  |
| Long, E. | ✔ | NA |  |  |  |  |  |  | ✔ |  | ✔ |  | ✔ | ✔ |  |
| Maheswaran, H. | ✔ | ✔ |  | ✔ |  |  |  |  | ✔ |  | ✔ | ✔ | ✔ | ✔ |  |
| Mandal, S. | ✔ | NA |  | ✔ |  |  |  |  | ✔ |  | ✔ | ✔ | ✔ | ✔ | ✔ |
| Marx, F. M. | ✔ | NA |  |  |  |  |  |  | ✔ | ✔ | ✔ | ✔ | ✔ | ✔ | ✔ |
| Masobe, P. | ✔ | ✔ |  |  |  |  |  |  | ✔ |  | ✔ |  | ✔ | ✔ |  |
| Mills, H. (2011) | ✔ | NA |  |  |  |  |  |  | ✔ |  | ✔ | ✔ |  | ✔ |  |
| Mills, H. (2013) | ✔ | NA |  |  |  |  |  |  | ✔ |  | ✔ | ✔ |  | ✔ |  |
| Perlman, D. C. | ✔ | ✔ |  |  |  |  |  |  | ✔ | ✔ | ✔ |  | ✔ | ✔ |  |
| Pho, M. | ✔ | ✔ | ✔ |  |  |  |  |  | ✔ | ✔ | ✔ |  | ✔ | ✔ | ✔ |
| Rhines, A. | ✔ | NA |  |  |  |  |  |  | ✔ | ✔ | ✔ |  | ✔ | ✔ | ✔ |
| Rose, D. (1992) | ✔ | NA |  |  |  |  |  |  | ✔ | ✔ | ✔ |  | ✔ | ✔ |  |
| Rose, D. N. (2000) | ✔ | NA |  |  |  |  |  |  | ✔ | ✔ | ✔ | ✔ | ✔ | ✔ |  |
| Samandari, T. | ✔ | ✔ | ✔ |  |  |  |  |  | ✔ | ✔ | ✔ |  | ✔ | ✔ | ✔ |
| Sawert, H. | ✔ |  |  |  |  |  |  |  | ✔ | ✔ | ✔ | ✔ | ✔ | ✔ |  |
| Shayo, G. | ✔ | ✔ |  |  |  |  |  |  | ✔ | ✔ | ✔ |  | ✔ | ✔ | ✔ |
| Shrestha. R. (2006) | ✔ | ✔ | ✔ |  |  |  |  |  | ✔ | ✔ | ✔ |  | ✔ | ✔ |  |
| Shrestha. R. (2007) | ✔ | ✔ | ✔ |  |  |  |  |  | ✔ | ✔ | ✔ |  | ✔ | ✔ |  |
| Smith, T. | ✔ | ✔ | ✔ |  |  |  |  |  | ✔ | ✔ | ✔ |  | ✔ | ✔ | ✔ |
| Snyder, D. C. | ✔ | ✔ |  |  |  |  |  |  | ✔ | ✔ | ✔ |  | ✔ | ✔ |  |
| Sterling, T. R. | ✔ | NA |  |  |  |  |  |  | ✔ |  | ✔ |  | ✔ | ✔ |  |
| Sumner, T. | ✔ | NA |  |  |  |  |  |  | ✔ |  | ✔ | ✔ | ✔ |  | ✔ |
| Sutton, B. | ✔ | ✔ | ✔ | ✔ |  |  |  |  | ✔ | ✔ | ✔ |  | ✔ | ✔ |  |
| Tasillo, A. | ✔ | ✔ |  |  | ✔ | ✔ | ✔ | ✔ | ✔ | ✔ | ✔ | ✔ | ✔ | ✔ |  |
| Terris-Prestholt, F. | ✔ | ✔ |  |  |  |  |  |  | ✔ | ✔ | ✔ |  | ✔ | ✔ | ✔ |
| Vynnycky, E. | ✔ | NA |  |  |  |  |  |  | ✔ | ✔ | ✔ | ✔ | ✔ |  | ✔ |
| Yan, I. | ✔ | NA |  | ✔ |  |  |  |  | ✔ | ✔ | ✔ | ✔ | ✔ | ✔ | ✔ |

Modelling assumptions and data sources for model parameters were essential components of our quality assessment checklist. As such, we illustrate a deeper analyses of these components below.

## Data sources

In Table S4, the use of either primary or secondary data sources for key input parameter categories are presented. For instance, the costs parameter seems to be most frequently derived from primary prospective or retrospective data sources (n=34; 51%), such as clinical trials, regional programs or clinics, government reports or data and interviews with clinic staff. In contrast, information on TB pathogenetic parameters (TB progression, reactivation, or relapse) mostly comes from secondary data sources (n=39; 91%), including published literature and unpublished reports, and only a few (n=4; 9%) are from primary data sources.

## Table D. Number of studies that used each type of data source for key input parameter categories

|  | ***KEY INPUT PARAMETER CATEGORIES*** | | | | |
| --- | --- | --- | --- | --- | --- |
| ***TYPE OF DATA SOURCE*** | ***Costs*** | ***TPT Cascade*** | ***TPT Efficacy*** | ***LTBI Prevalence*** | ***TB Progression, Reactivation or Relapse*** |
| **PRIMARY PROSPECTIVE DATA** | | | | | |
| Clinical trial | 6 | 8 | 8 | 1 | 1 |
| Regional program or clinic | 7 | 5 | 3 | 3 | 0 |
| Government report/data | 10 | 2 | 1 | 1 | 3 |
| Interviews with clinic staff | 3 | 1 | 1 | 0 | 0 |
| **PRIMARY RETROSPECTIVE DATA** | | | | | |
| Program evaluation | 2 | 3 | 1 | 0 | 0 |
| Hospital/clinic records | 6 | 1 | 0 | 0 | 0 |
| **SECONDARY DATA** | | | | | |
| Published literature | 20 | 17 | 36 | 14 | 35 |
| Unpublished reports (e.g. from NGOs) | 7 | 1 | 1 | 4 | 4 |

*TPT = tuberculosis preventive therapy, LTBI = latent tuberculosis infection, TB = tuberculosis, NGO = non-governmental organization*

## Input parameters and assumptions

Building on the input parameter categories and their data sources, we compared the actual values of parameters across studies. There were several parameters that were common inputs across models, such as LTBI prevalence and TPT efficacy. We differentiated between studies that based input parameter values on data, and studies that based values on assumptions (without citing any sort of data). There were some differences seen between data-informed values and assumed values. For example, LTBI prevalence tended to be lower when informed by data, although its range overlapped with the range of assumed values. Similarly, TPT efficacy and duration of TPT efficacy tended to be lower when informed by data; there was less overlap in their ranges, meaning there was a larger difference between data-informed values and assumed values. Studies that didn’t cite data for TPT efficacy and duration of TPT efficacy tended to overestimate them. This was in contrast to the rate of TPT completion or adherence, which tended to be *underestimated* by studies that didn’t use data to inform its value. Overall, there is evident heterogeneity across studies in terms of model parameters.

## Table E. Comparing input parameters that were based on data to those that were based on assumptions

|  | **Parameter based on data** | | **Parameter based on assumption** | |
| --- | --- | --- | --- | --- |
| **Parameter** | **Number of studies** | **Median (Q1 - Q3) of parameter estimate** | **Number of studies** | **Median (Q1 - Q3) of parameter estimate** |
| LTBI prevalence | 31 | 0.33 (0.16 - 0.5) | 5 | 0.4 (0.3 - 0.62) |
| Probability of progression to active TB | 22 | 0.11 (0.05 - 0.21) | 4 | 0.13 (0.09 - 0.21) |
| Probability of reactivation to active TB | 21 | 0.05 (0.02 - 0.1) | 5 | 0.06 (0.04 - 0.08) |
| Rate of TPT completion/adherence* | 25 | 0.8 (0.65 - 0.89) | 5 | 0.72 (0.67 - 0.79) |
| TPT efficacy in preventing active TB* | 49 | 0.68 (0.6 - 0.85) | 5 | 0.83 (0.83 - 1) |
| Duration of TPT efficacy (years after completion of TPT)* | 14 | 0 years (0 years - 2 years) | 10 | 3 years (1 years - 5 years) |
| Probability of adverse event from TPT** | 17 | 0.006 (0.001 - 0.028) | 3 | 0.0031 (0.0017 - 0.0046) |
| TST sensitivity | 6 | 0.78 (0.62 - 0.89) | 0 | NA |
| TST specificity | 7 | 0.95 (0.93 - 0.97) | 0 | NA |
| IGRA sensitivity | 6 | 0.8 (0.65 - 0.85) | 0 | NA |
| IGRA specificity | 5 | 0.99 (0.98 - 0.99) | 0 | NA |
| Cost of TPT per person (2020 USD)* | 33 | $46.99 ($28.75 - $279.99) | 2 | $88.93 ($79.61 - $98.24) |
| Cost of TST per person (2020 USD) | 15 | $8.51 ($2.72 - $21.16) | 0 | NA |
| Cost of IGRA per person (2020 USD) | 5 | $78.36 ($66.21 - $86.86) | 1 | $59.01 ($59.01 - $59.01) |
| Cost of adverse event (2020 USD)** | 15 | $1520.68 ($329.28 - $6706.66) | 0 | NA |

*LTBI = latent tuberculosis infection, TB = tuberculosis, TPT = tuberculosis preventive therapy, TST = tuberculin skin test, IGRA = interferon gamma release assay, USD = United States Dollars*

*The probability of an adverse event from TPT is a time-associated probability; as long as an individual is given TPT, there exists a probability of occurrence of an adverse event. The probabilities of progression and reactivation are also time-associated*

** Reflects all TPT regimens*

*** Reflects the probability and costs of severe adverse event, which included generic treatment for hepatotoxicity (median probability of occurring = 0.005), acquired drug resistance (median probability of occurring = 0.010), or symptoms of rash, pruritus, gastrointestinal distress, and arthralgia (median probability of occurring = 0.045)*

# DESCRIPTION OF INCLUDED STUDIES

## Table F. Description of included studies

| **Study Design** | **First Author & Year of Publication** | **Costing Perspective** | **Study Setting** | **Baseline TB incidence in Study Setting** | **Baseline HIV prevalence in Study Setting** | **Population** | **Analytic Horizon** | **TPT Regimens Included*** | **Indication for TPT using LTBI test?** |
| --- | --- | --- | --- | --- | --- | --- | --- | --- | --- |
| **Studies that report cost and cost-effectiveness outcomes** | | | | | | | | | |
| Cost Effectiveness Analysis | Azadi, M.  2014 | Health System | Rio de Janeiro, Brazil | 95 per 100,000 | 10% (among TB cases) | PLHIV | 20 years | 6 INH | Yes (TST) |
| Cost Effectiveness Analysis | Bell, J.  1999 | Societal | Uganda | NR | NR | PLHIV | 8.4 years | 6 INH, 3 INH RIF | No |
| Dynamic Modelling | Awoke, T. D.  2018 | NR | Global/no specific setting | NR | NR | General (PLHIV subset) | 10 years | Regimen not specified | No |
| Cost Effectiveness Analysis | Bachmann, M. O.  2006 | Health System | South Africa | 1,992 per 100,000 | Approx. 10% | PLHIV | 10 years | 6 INH | No |
| Cost Effectiveness Analysis | Burgos, J.  2009 | Health System | Tijuana, Mexico | 57 per 100,000 | NR | General (PLHIV subset) | 20 years | 6 INH | Yes (IGRA) |
| Dynamic Modelling | Currie, C.  2005 | Health System | Kenya | 577 per 100,000 | 29% (among 15-49 year olds) | General (PLHIV subset) | 20 years | 6 INH, Lifetime INH | No |
| Cost Analysis | de Siqueira Filha, N. 2018 | Patient | Recife, Brazil | NR | NR | PLHIV | 1 year | 12 INH | Yes (TST) |
| Cost Analysis | de Siqueira Filha, N. 2018 | Health System | Recife, Brazil | NR | NR | PLHIV | 1 year | 12 INH | Yes (TST) |
| Cost Effectiveness Analysis | Ferguson, O. 2020 | Health System | Uganda | NR | NR | PLHIV | 20 years | 1 INH RPT, 3 INH RPT | No |
| Cost Effectiveness Analysis | Foster, S.  1997 | Health System | VTC site in Zambia | 400 per 100,000 | 30% (among patients) | PLHIV | 5 years | 6 INH, Lifetime INH | Yes (TST) & No |
| Cost Effectiveness Analysis | Gilbert, J. A.  2016 | Health System | Rural South Africa | 838 per 100,000 | 10.2% | General (PLHIV subset) | 10 years | 36 INH, 12 INH, Lifetime INH | Yes (TST) & No |
| Cost Effectiveness Analysis | Gourevitch, M.  1998 | Health System | Bronx, New York, USA | NR | NR | People who use drugs (PLHIV subset) | 5 years | 12 INH | Yes (TST) |
| Cost Effectiveness Analysis | Gupta, S.  2014 | Health System | South Africa | 3,950 per 100,000 | NR | PLHIV | 3 years | 36 INH, 6 INH | Yes (TST) & No |
| Cost Effectiveness Analysis | Hausler, H. P.  2006 | Health System | Cape Town, South Africa | 488 per 100,000 | 17% | General (PLHIV subset) | 1 year | 6 INH | No |
| Dynamic Modelling | Hsieh, Y. L. | Health System | Malawi | 246.2 per 100,000 | 8.8% | General (PHLIV subset) | 12 years | 6 INH, Lifetime INH | No |
| Dynamic Modelling | Jo, Y. | Health System | USA (California, Florida, New York, Texas) | 5.3 per 100,000 (California) | NR | General (PLHIV subset) | 30 years | 3 INH RPT | Yes (IGRA) |
| Cost Effectiveness Analysis | Johnson, K.  2018 | Health System | Uganda | NR | NR | PLHIV | 20 years | 3 INH RPT, 9 INH | No |
| Cost Effectiveness Analysis | Kapoor, S.  2016 | Health System | India | NR | NR | PLHIV pregnant women | 12 years | 6 INH | No |
| Cost Effectiveness Analysis | Kim, H-Y.  2018 | Health System | South Africa | NR | NR | PLHIV pregnant women | 1 year | 12 INH | Yes (TST, IGRA) & No |
| Cost Effectiveness Analysis | Kowada, A.  2013 | Health System | Low TB incidence countries | 1,800 per 100,000 (among close contacts) | NR | PLHIV pregnant women | 30 years | 6 INH | Yes (TST, IGRA) |
| Cost Effectiveness Analysis | Linas, B.  2011 | Health System | USA | NR | NR | General (PLHIV subset) | 20 years | 9 INH | Yes (TST, IGRA) & No |
| Cost Effectiveness Analysis | Maheswaran, H.  2012 | Health System | Sub-Saharan Africa | NR | NR | PLHIV | 2 years | 6 INH | No |
| Least Cost Analysis | Masobe, P.  1995 | Health System | South Africa | NR | NR | PLHIV | 8 years | 6 INH | Yes (TST) & No |
| Cost Effectiveness Analysis | Perlman, D. C.  2001 | Health System | New York City, USA | NR | NR | People who use drugs (PLHIV subset) | 5 years | 9 INH | Yes (TST) |
| Cost Effectiveness Analysis | Pho, M.  2012 | Health System | India | 6,900 per 100,000 | NR | PLHIV | 10 years | 36 INH | No |
| Cost Effectiveness Analysis | Samandari, T.  2011 | Health System | Botswana | 4,1000 per 100,000 | 25% (among 15-49 year olds) | PLHIV | 3 years | 6 INH | No |
| Cost Effectiveness Analysis | Sawert, H.  1998 | Health System | Italy (to be generalized to other HICs) | NR, | NR | PLHIV | 10 years | INH (Duration not specified) | Yes (TST) & No |
| Cost Effectiveness Analysis | Shayo, G.  2018 | Provider | Tanzania | 511 per 100,000 | NR | PLHIV | 2 years | 6 INH | No |
| Cost Effectiveness Analysis | Shrestha. R.  2006 | Program | Kampala, Uganda | 35-162 per 100,000 | NR | PLHIV | 2 years | 9 INH, 6 INH | Yes (TST) & No |
| Cost Effectiveness Analysis | Shrestha. R.  2007 | Program | Kampala, Uganda | NR | NR | PLHIV | 2 years | 9 INH, 6 INH | Yes (TST) & No |
| Cost Effectiveness Analysis | Smith, T.  2015 | Health System | Botswana | NR | NR | PLHIV | 3 years | 36 INH, 6 INH | Yes (TST) & No |
| Cost Effectiveness Analysis | Snyder, D. C.  1999 | Program | San Francisco, California, USA | NR | 21% (among those who began IPT) | People who use drugs (PLHIV subset) | 10 years | 12 INH | Yes (TST) |
| Cost Analysis | Sutton, B.  2009 | Third-Party Payer | Cambodia | NR | 13% (among those with TB) | PLHIV | 3 years | 9 INH | No |
| Cost Effectiveness Analysis | Tasillo, A.  2017 | Health System | USA | NR | NR | General (PLHIV subset) | Lifetime (Expected 34 years) | 3 INH RPT | Yes (TST, IGRA) & No |
| Cost Analysis | Terris-Prestholt, F. 2008 | Provider | Zambia | 653 per 100,000 | 22% | PLHIV | 2 years | 6 INH | No |
| Econometric Analysis | Yan, I.  2016 | NA | 41 high HIV-TB burden countries | Various | Various | General (PLHIV subset) | 15 years | INH (Duration not specified) | Varies by country |
| **Studies that report effectiveness or utility outcomes only** | | | | | | | | | |
| Dynamic Modelling | Basu, S.  2009 | NA | Botswana | 551 per 100,000 | 23.9% (among adults) | PLHIV | 47 years | INH (Duration not specified) | Yes (TST) & No |
| Dynamic Modelling | Bacaer, N.  2008 | NA | Township near Cape Town, South Africa | 2,000 per 100,000 | >20% | General (PLHIV subset) | 100 years | INH (Duration not specified) | No |
| Dynamic Modelling | Brewer, T.  2001 | NA | USA | 90 per 100,000 (among PLHIV) | 13.58% (among people experiencing homelessness) | General and people experiencing homelessness (PLHIV subset) | 10 years | INH (Duration not specified) | No |
| Effectiveness Analysis | Brewer, T.  1996 | NA | USA | NR | 0.39% | General (PLHIV subset) | 10 years | INH (Duration not specified) | Yes (TST) |
| Dynamic Modelling | Cohen, T.  2016 | NA | High TB-HIV burden settings, no specific setting | NR | 16.3% | General (PLHIV subset) | 30 years | INH (Duration not specified) | No |
| Dynamic Modelling | Dowdy, D.  2014 | NA | Rio de Janeiro, Brazil | 122 per 100,000 (among 15-19 year olds) | NR | PLHIV | 5 years | 6 INH | No |
| Dynamic Modelling | Dye, C.  2013 | NA | South Africa, India, China, USA | South Africa: 980 per 100,000; NR for other countries | NR | General (PLHIV subset) | 40 years | INH (Duration not specified) | Yes (TST, IGRA) |
| Effectiveness Analysis | Freiman, J.  2018 | NA | Brazil, India, Uganda | Varies by duration of ART | NR | PLHIV | 36-43 years | 6 INH, 36 INH | No |
| Dynamic Modelling | Guwatudde, D.  2004 | NA | Uganda | 138 per 100,000 | 10% | General (PLHIV subset) | 20 years | INH (Duration not specified) | Yes (TST) |
| Dynamic Modelling | Heymann, S. J.  1993 | NA | Sub-Saharan Africa | NR | NR | General (PLHIV subset) | 10 years | INH (Duration not specified) | Yes (TST) |
| Dynamic Modelling | Houben, R.  2014 | NA | Kampala, Uganda; Nairobi, Kenya; Soweto, South Africa | NR | NR | PLHIV | 3 years for Kampala and Soweto, 2 years for Nairobi | 6 INH, 3 INH RPT, 3 INH RIF | Yes (TST) |
| Effectiveness Analysis | Jordan, T. J.  1991 | NA | USA | NR | NR | PLHIV | Lifetime (Expected 9 years) | INH (duration not specified) | Yes (TST) & No |
| Dynamic Modelling | Kendall, E.  2019 | NA | Khayelitsha, South Africa | 1,600 per 100,000 | 33% (antenatal) | PLHIV | 5 years | 12 INH, Lifetime INH | No |
| Dynamic Modelling | Kunkel, A.  2016 | NA | Botswana | 400-900 per 100,000 | 15-70% | PLHIV | 34 years | 6 INH | Yes (TST) & No |
| Dynamic Modelling | Long, E.  2007 | NA | India (southern states) | NR | 1.3% | General (PLHIV subset) | 20 years | INH (Duration not specified) | No |
| Dynamic Modelling | Mandal, S. 2020 | NA | WHO South-East Asian Region | 218 per 100,000 | Various | General (PLHIV subset) | 12 years | 3 INH RPT** | No |
| Dynamic Modelling | Marx, F. M.  2018 | NA | Cape Town, South Africa | NR | 5.2% (among adults) | Those who previously completed TB treatment (PLHIV subset) | 10 years | Lifetime INH | No |
| Dynamic Modelling | Mills, H.  2013 | NA | Lesotho | NR | 28% | General (PLHIV subset) | 100 years | 6 INH | No |
| Dynamic Modelling | Mills, H.  2011 | NA | No specific setting, but results mimic epidemics in Sub-Saharan Africa | NR | NR | General (PLHIV subset) | 10 years | 9 INH | No |
| Dynamic Modelling | Rhines, A.  2018 | NA | South Africa | 1,117 per 100,000 | 15% | General (PLHIV subset) | 20 years | IPH (Duration not specified) | No |
| Dynamic Modelling | Rose, D.  1992 | NA | USA | Varies | NR | PLHIV | 10 years | 12 INH | Yes (TST) |
| Effectiveness Analysis | Rose, D. N.  2000 | NA | USA | 196 per 100,000 within 1 year of being infected | NR | General (PLHIV subset) | 10 years and lifetime | 6 INH, 12 INH | Yes (TST) & No |
| Effectiveness Analysis | Sterling, T. R.  1999 | NA | No particular setting, but generalizable to the "developing world" | 1,7000 per 100,000 | NR | PLHIV | NR | 12 INH | Yes (TST) |
| Dynamic Modelling | Sumner, T.  2016 | NA | South Africa | NR | NR | PLHIV | 2 years | 12 INH | No |
| Dynamic Modelling | Vynnycky, E.  2014 | NA | South Africa | 800-1200 per 100,000 | 30% | Gold mine workers (PLHIV subset) | 5 years | 9 INH | No |

TB = tuberculosis, HIV = human immunodeficiency virus, TPT = tuberculosis preventive therapy, LTBI = latent tuberculosis infection, PLHIV = people living with HIV, INH = isoniazid, TST = tuberculin skin test, IGRA = interferon gamma release assay, RPT = rifapentine, RIF = rifampin, NA = not applicable, NR = not reported

* The numbers beside each regimen indicates how many months that regimen was given for. Studies may have included other TPT regimens, but the results for those regimens were not reported in the study, so they are not listed in this table

** This article modelled a “3HP-like regimen”; the goal was to model a shorter, rifamycin based regimen

## Table G. Key outcomes among studies that report effectiveness or utility outcomes only

| First Author (Year Published) | Setting | Analytical Horizon | TPT Regimen* | Comparison Made | Key Outcome Reported | Value of Key Outcome Reported |
| --- | --- | --- | --- | --- | --- | --- |
| Global Setting | | | | | | |
| Time Horizon > 5 years | | | | | | |
| INH-Based Regimen > 6 months | | | | | | |
| Sterling, T. R. (1999) | No particular setting | NR | 12 INH | TPT indicated by TST vs. no TPT | Relative reduction in active TB incidence | 70% |
| INH-Based Regimen, Duration Not Specified | | | | | | |
| Cohen, T. (2016) | Settings with high TB incidence | 30 years | INH** | TPT^†^ vs. no TPT | Relative reduction in active TB incidence | 76% |
| Yan, I. (2016) | 41 countries with high TB incidence | 15 years | INH** | 30% TPT coverage vs. no TPT | Relative reduction in mortality | 5 fewer deaths per 10,000 population |
| RIF-Based Regimen | | | | | | |
| Mandal, S.  (2020) | WHO South-East Asian Region | 12 years | 3 INH RPT | TPT vs. no TPT | Relative reduction in active TB incidence | 12% |
| Low- and Middle-Income Settings | | | | | | |
| Time Horizon < 5 years | | | | | | |
| INH-Based Regimen > 6 months | | | | | | |
| Sumner, T. (2016) | South Africa | 2 years | 12 INH | TPT vs. no TPT | Relative reduction in active TB incidence | 21% |
| Multiple TPT Regimens | | | | | | |
| Houben, R. (2014) | Kenya^††^ | 2 years | 6 INH | TPT (6 INH) vs. no TPT | Estimated annual risk of infection in Kenya | 4.9% |
|  |  |  | 3 INH RIF | TPT (3 INH RIF) vs. no TPT | Estimated annual risk of infection in Kenya | 3.7% |
| Time Horizon $\boldsymbol{\geq}$ 5 years | | | | | | |
| INH-Based Regimen $\boldsymbol{\leq}$ 6 months | | | | | | |
| Freiman, J. (2018) | Brazil^††^ | 43 years | 6 INH | TPT vs. no TPT | Relative reduction in active TB incidence in Brazil | 19% |
| Mills, H. (2013) | Lesotho | 100 years | 6 INH | TPT vs. no TPT | Relative reduction in prevalence of latent DS-TB | 71% |
| INH-Based Regimen > 6 months | | | | | | |
| Mills, H. (2011) | Sub-Saharan Africa | 10 years | 9 INH | 50% TPT coverage vs. no TPT | Relative reduction in active TB incidence | 77% |
| Vynnycky, E. (2014) | South Africa | 5 years | 9 INH | TPT vs. no TPT | Relative reduction in active TB incidence | 29% |
| Marx, F. M. (2018) | South Africa | 10 years | Lifelong INH | TPT vs. no TPT | Relative reduction in active TB incidence | 71% |
| Multiple TPT Regimens | | | | | | |
| Kendall, E. (2019) | South Africa | 5 years | 12 INH | TPT (12 INH) vs. no TPT | Relative reduction in active TB incidence | 5% |
|  |  |  | Lifelong INH | TPT (Lifelong INH) vs. no TPT | Relative reduction in active TB incidence | 11% |
| Kunkel, A. (2016) | Botswana | 34 years | 6 INH | TPT (6 INH) vs. no TPT | Relative reduction in active DS-TB incidence | 12% |
|  |  |  | Lifelong INH | TPT (Lifetime INH) vs. no TPT | Relative reduction in active DS-TB incidence | 46% |
| INH-Based Regimen, Duration Not Specified | | | | | | |
| Basu, S. (2009) | Botswana | 47 years | INH | TPT vs. TPT indicated by TST | Relative reduction in active TB incidence | 54% |
| Bacaer, N. (2008) | South Africa | 100 years | INH | TPT vs. no TPT | Relative reduction in active TB incidence | 47% |
| Dowdy, D. (2014) | Brazil | 5 years | INH | TPT indicated by TST vs. no TPT | Percent reduction in active TB incidence | 16% |
| Dye, C. (2013) | South Africa, India, China^††^ | 40 years | INH | TPT vs. no TPT | Relative reduction in active TB incidence in South Africa | 73% |
|  |  |  |  |  | Relative reduction in active TB incidence in India | 100% |
|  |  |  |  |  | Relative reduction in active TB incidence in China | 100% |
| Guwatudde, D. (2004) | Uganda | 20 years | INH | TPT vs. no TPT | Relative reduction in TB-related mortality | 22% |
| Heymann, S. J. (1993) | Sub-Saharan Africa | 10 years | INH | 30% TPT coverage vs. no TPT | Relative reduction in proportion of original population with new latent TB infection | 98% |
| Long, E. (2007) | India | 20 years | INH | TPT vs. no TPT | Relative reduction in active TB incidence | 100% |
| Rhines, A. (2018) | South Africa | 20 years | INH | 90% TPT coverage vs. 5% TPT Coverage | Relative reduction in active TB incidence | 40% |
| High-Income Settings | | | | | | |
| Time Horizon $\boldsymbol{\geq}$ 5 years | | | | | | |
| INH-Based Regimen $\boldsymbol{\leq}$ 6 months | | | | | | |
| Rose, D. N. (2000) | USA | 10 years | 6 INH | TPT vs. TPT indicated by TST | Relative reduction in active TB incidence (reported only as a range) | 41-99% |
| INH-Based Regimen > 6 months | | | | | | |
| Rose, D. (1992) | USA | 10 years | 12 INH | TPT vs. no TPT | Relative reduction in active TB incidence | 42% |
| INH-Based Regimen, Duration Not Specified | | | | | | |
| Jordan, T. J. (1991) | USA | Lifetime | INH | TPT vs. no TPT | Relative increase in life expectancy for black men with unknown TST status | 2.5% |
| Brewer, T. (2001) | USA | 10 years | INH | 10% increase in access to TPT vs. baseline access to TPT | Relative reduction in active TB incidence among people living with AIDS experiencing chronical homelessness | 8.9% |
| Brewer, T. (1996) | USA | 10 years | INH | 10% increase in TPT efficacy vs. baseline TPT efficacy | Percent reduction in active TB incidence among people living with AIDS | 6.2% |

* The numbers beside each regimen indicate the number of months that regimen was given
** Where the TPT regimen just states “INH”, it means that the study did not specify the duration
^†^ When the strategies compared just lists “TPT”, it implies that TPT was not indicated by any test, and was provided to all PLHIV
^††^ These studies had additional settings in which TPT delivery was modelled, but results for key outcomes were not reported
*Abbreviations: NR = not reported, ART = Antiretroviral Therapy, HIV = Human Immunodeficiency Virus, PLHIV = People Living with HIV, INH = Isoniazid, RIF = rifampin, RPT = Rifapentine, QALY = Quality adjusted life years, DALY = Disability adjusted life years, TB = Tuberculosis, DS-TB = Drug-Sensitive Tuberculosis, TPT = Tuberculosis Preventative Therapy, TST = Tuberculin Skin Test, IGRA = Interferon Gamma Release Assay*

## Table H. Key outcomes and results of studies that reported costs and cost-effectiveness outcomes (2020 USD)

| First Author (Year Published) | Setting | Analytical Horizon | TPT Regimen* | Comparison Made | Key Outcome Reported | Value of Key Outcome Reported | Incremental Net Monetary Benefit (Calculated)** |
| --- | --- | --- | --- | --- | --- | --- | --- |
| Global Setting | | | | | | | |

| Kowada, A. (2013) | Countries with low TB incidence | 30 years | 6 INH | TPT indicated by IGRA vs. TPT indicated by TST | Incremental cost per active TB case averted | Cost-saving | NA |
| --- | --- | --- | --- | --- | --- | --- | --- |
| Awoke, T. D. (2018) | Global setting | 10 years | TPT^†^ | TPT^††^ vs. no TPT | Relative marginal program costs | Cost-saving | NA |

| Low- and Middle-Income Countries | | | | | | | |
| --- | --- | --- | --- | --- | --- | --- | --- |
| Time Horizon < 5 years | | | | | | | |
| INH-Based Regimen $\boldsymbol{\leq}$ 6 months | | | | | | | |
| Hausler, H. P. (2006) | South Africa | 1 year | 6 INH | TPT vs. no TPT | Incremental cost per active TB case averted | $700 | $1,353 |
| Shayo, G. (2018) | Tanzania | 2 years | 6 INH | TPT vs. no TPT | Incremental cost per active TB case averted | $152 | $224 |
| Samandari, T. (2011) | Botswana | 3 years | 6 INH | Symptom screen and CXR to rule out active TB before TPT vs. symptom screen to rule out active TB before TPT | Incremental cost per active TB case averted | $9,547 | NA |
| Maheswaran, H. (2012) | Sub-Saharan Africa | 2 years | 6 INH | CXR to rule out active TB before TPT vs. chronic cough to rule out active TB before TPT | Incremental cost per QALY gained | $6,743 | NA |
| Terris-Prestholt, F. (2008) | Zambia | 2 years^‡^ | 6 INH | TPT (no comparison made) | Cost of TPT in Chawama (Site #1) ^‡‡^ | $1,716 | NA |
|  |  |  |  |  | Cost of TPT in Matero (Site #2) ^‡‡^ | $1,624 | NA |
| INH-Based Regimen > 6 months | | | | | | | |
| Kim, H-Y. (2018) | South Africa | 1 year | 12 INH | TPT indicated by TST vs. TPT for all | Incremental cost per active TB case averted | $1,687 | NA |
|  |  |  |  | TPT indicated by IGRA vs. TPT for all | Incremental cost per active TB case averted | $2,933 | NA |
| de Siqueira Filha, N. (2018) | Brazil | 1 year | 12 INH | TPT (no comparison made) | Total health system costs per patient during treatment | $139 | NA |
| de Siqueira Filha, N. (2018) | Brazil | 1 year | 12 INH | TPT (no comparison made) | Total patient costs per patient | $660 | NA |
| Shrestha. R. (2007) | Uganda | 2 years | 9 INH | TPT indicated by TST vs. TPT for all | Incremental cost per QALY gained | $149 | NA |
| Shrestha. R. (2006) | Uganda | 2 years | 9 INH | TPT indicated by TST vs. no TPT | Incremental cost per active TB case treated | $297 | $480 |
|  |  |  |  | TPT vs. no TPT | Incremental cost per active TB case treated | $1,082 | -$112 |
| Sutton, B. (2009) | Cambodia | 3 years^‡^ | 9 INH | TPT vs. no TPT | Incremental cost per active TB case averted | $1,149 | $71 |
| Multiple TPT Regimens | | | | | | | |
| Gupta, S. (2014) | South Africa | 3 years | 36 INH vs. 6 INH | TPT (36 INH) vs. TPT (6 INH) | Incremental cost per active TB case averted | $11,516 | NA |
| Smith, T. (2015) | Botswana | 3 years | 36 INH if TST+  6 INH if TST- | TPT indicated by TST vs. TPT for all | Incremental cost per active TB case averted | $7,782 | NA |
| Time Horizon $\boldsymbol{\geq}$ 5 years | | | | | | | |
| INH-Based Regimen $\boldsymbol{\leq}$ 6 months | | | | | | | |
| Azadi, M. (2014) | Brazil | 20 years | 6 INH | TPT vs. no TPT | Incremental cost per active TB case averted | $2,603 | $94 |
| Burgos, J. (2009) | Mexico | 20 years | 6 INH | TPT vs. no TPT | Incremental cost per active TB case averted | $658 | $737 |
| Masobe, P. (1995) | South Africa | 8 years | 6 INH | TPT vs. no TPT | Incremental cost per active TB case averted | $513 | $4,399 |
| Bachmann, M. O. (2006) | South Africa | 10 years | 6 INH | TPT indicated by CD4 cell count vs. no TPT | Incremental cost per life year gained | Cost-saving | NA |
| Kapoor, S. (2016) | India | 12 years | 6 INH | TPT for all vs. TPT indicated by CD4 cell count | Incremental cost per active TB case averted | $1,110 | NA |
| INH-Based Regimen > 6 months | | | | | | | |
| Johnson, K. (2018) | Uganda | 20 years | 9 INH | TPT vs. no TPT | Incremental cost per DALY averted | $281 | NA |
| Pho, M. (2012) | India | 10 years | 36 INH | TPT vs. no TPT | Incremental cost per active TB case averted | $15,166 | -$135 |
| RIF-Based Regimen | | | | | | | |
| Ferguson, O.  (2020) | Uganda | 20 years | 1 INH RPT vs. 3 INH RPT | TPT (1 INH RPT) vs. TPT (3 INH RPT) | Incremental cost per DALY averted | $1,236 | NA |
| Multiple TPT Regimens | | | | | | | |
| Bell, J. (1999) | Uganda | 8.4 years | 6 INH | TPT (6 INH) vs. no TPT | Incremental cost per active TB case averted | $479 | $443 |
|  |  |  | 3 INH RIF | TPT (3 INH RIF) vs. no TPT | Incremental cost per active TB case averted | $114 | $49 |
| Currie, C. (2005) | Kenya | 20 years | 6 INH | TPT (6 INH) vs. no TPT | Incremental cost per active TB case averted | $412 | $287 |
|  |  |  | Lifetime INH | TPT (Lifetime INH) vs. no TPT | Incremental cost per active TB case averted | $1,925 | -$4 |
| Foster, S. (1997) | Zambia | 5 years | 6 INH | TPT through TB clinic vs. TPT through VCT | Incremental cost per active TB case averted | Cost-saving | NA |
|  |  |  | Lifetime INH |  | Incremental cost per active TB case averted | Cost-saving | NA |
| Hsieh, Y. L.  (2020) | Malawi | 12 years | 6 INH | TPT vs. no TPT | Incremental cost per active TB case averted | $285 | $3 |
|  |  |  | Lifetime INH |  | Incremental cost per active TB case averted | $1,086 | -$15 |
| Gilbert, J. A. (2016) | South Africa | 10 years | 36 INH if TST+  12 INH if TST- | TPT for all vs. TPT indicated by TST | Incremental cost per active TB case averted | $17,836 | NA |
| High-Income Countries | | | | | | |  |
| Time Horizon $\boldsymbol{\geq}$ 5 years | | | | | | | |
| INH-Based Regimen > 6 months | | | | | | | |
| Gourevitch, M. (1998) | USA | 5 years | 12 INH | TPT vs. no TPT | Incremental cost per active TB case averted | $16,249 | $1,081 |
| Snyder, D. C. (1999) | USA | 10 years | 12 INH | TPT vs. no TPT | Incremental cost per active TB averted | Cost-saving | $7,581 |
| Perlman, D. C. (2001) | USA | 5 years | 9 INH | TPT vs. no TPT | Incremental cost per active TB case averted | Cost-saving | $43,465 |
| INH-Based Regimen, Duration Not Specified | | | | | | | |
| Linas, B. (2011) | USA | 20 years | INH | TPT indicated by TST vs. TPT for all | Incremental cost per QALY gained | $14,744 | NA |
|  |  |  |  | TPT indicated by IGRA vs. no TPT | Incremental cost per QALY gained | $27,415 | NA |
| Sawert, H. (1998) | Italy | 10 years | INH | TPT indicated by TST vs. TPT for all | Incremental cost per QALY gained | $23,255 | NA |
| RIF-Based Regimen | | | | | | | |
| Tasillo, A. (2017) | USA | 34 years | 3 INH RPT | TPT indicated by TST and IGRA vs. TPT for all | Incremental cost per QALY gained | $19,667 | NA |
| Jo, Y.  (2020) | USA | 30 years | 3 INH RPT | TPT vs. no TPT | Incremental cost per active TB case averted in California | $9,609 | $3,589 |
|  |  |  |  |  | Incremental cost per active TB case averted in Florida | $4,564 | $11,043 |
|  |  |  |  |  | Incremental cost per active TB case averted in New York | $15,819 | $3,759 |
|  |  |  |  |  | Incremental cost per active TB case averted in Texas | $7,090 | $12,304 |

* The numbers beside each regimen indicate the number of months that regimen was given
** Incremental net monetary benefit was calculated for studies that compared some form of TPT (indicated or not) vs. no TPT, and that reported incremental cost per active TB case averted. In some studies, a point estimate for incremental cost per active TB case averted was calculated from reported costs and active TB cases; in those cases an incremental net monetary benefit was also calculated and included in subsequent analysis, but it is not shown in this table as the incremental cost per active TB case averted was not the “key outcome reported” in those studies
^†^ Where the TPT regimen just states “TPT”, it means that the study did not specify the regimen. Where it states “INH”, it means that the study did not specify the duration
^††^ When the strategies compared just lists “TPT”, it implies that TPT was not indicated by any test, and was provided to all PLHIV
^‡^ These are not modelling studies; the analytic horizon indicates the follow-up period that costing analysis was done for ^‡‡^ The two sites differed slightly in their location: Chawama's clinic was in stand-alone buildings, while Matero’s clinic was situated within the main hospital building
*Abbreviations: USD = United States Dollars, ART = Antiretroviral Therapy, HIV = Human Immunodeficiency Virus, PLHIV = People Living with HIV, INH = Isoniazid, RPT = Rifapentine, QALY = Quality adjusted life years, DALY = Disability adjusted life years, TB = Tuberculosis, TPT = Tuberculosis Preventative Therapy, TST = Tuberculin Skin Test, IGRA = Interferon Gamma Release Assay, CXR = chest x-ray, VCT = voluntary counselling and HIV testing services*

## Table I. Detailed outcomes of studies that reported cost and cost-effectiveness outcomes

| **Study Author & Currency (Country & Year)** | **Strategies Compared*** | **Cost Outcomes** | | | **Effectiveness Outcomes** | | | **Cost-Effectiveness Outcomes** | | |
| --- | --- | --- | --- | --- | --- | --- | --- | --- | --- | --- |
|  |  | **Outcome** | **Strategy** | **Value**  **(95% CI)** | **Outcome** | **Strategy** | **Value**  **(95% CI)** | **Outcome** | **Strategy** | **Value**  **(95% CI)** |
| Awoke, T. D., NR | (1) No mitigation strategy; (2) Preventive education on TB disease and AIDS (no PLHIV on ARV in this scenario); (3) Treating latently infected individuals with TPT (all PLHIV on ARV in this scenario); (4) Preventive education, TPT and ARV together | Cost over 10 years (x10,000) | 1 | $4.92 | Prevalence of TB in year 0 vs. year 10 | 1 | 6.3% vs. 17.3% |  | | |
|  |  |  | 2 | $2.90 |  | 2 | 6.3% vs. 4.8% |  |  |  |
|  |  |  | 3 | -$1.07 |  | 3 | 6.3% vs. 0.3% |  |  |  |
|  |  |  | 4 | -$1.36 |  | 4 | 6.3% vs. 0.2% |  |  |  |
| Azadi, M., 2010 USD | (1) THRio: training HIV clinics in using TST and 6 INH; (2) Usual care | Median discounted cost including intervention implementation, diagnosis, follow-up, and therapy in 100 patients followed 20 years | 1 | $9,748 ($9,530 - $10,078) | Discounted DALYs per 100 patients followed over 20 years | 1 | 580.88 | Incremental cost per DALY averted | (1) vs. (2) | $2,273 ($1,179 - $3,135) |
|  |  |  |  |  |  | 2 | 582.02 |  |  |  |
|  |  |  | 2 | $6461 ($6,278 - $6,783) | Active TB Cases | 1 | 10 | Incremental cost per TB case averted | (1) vs. (2) | $2,191 |
|  |  |  |  |  |  | 2 | 11.5 |  |  |  |
| Bachmann, M. O., 2005 USD | (1) No prevention; (2) late IPT; (3) early IPT; (4) late IPT + CPT (cotrimoxazole preventive therapy) ("late" is when CD4 = 200 cells/ul, "early" is when CD4 = 350 cells/ul) | Mean lifetime healthcare cost | 1 | $2,952 | Mean survival (years) | 1 | 8.2 | Incremental cost per life year gained | (2) vs. (1) | -$430 |
|  |  |  | 2 | $2,784 |  | 2 | 8.6 |  | (3) vs. (1) | -$70 |
|  |  |  | 3 | $2,900 |  | 3 | 9 |  | (4) vs. (1) | $17 |
|  |  |  | 4 | $2,962 |  | 4 | 8.8 |  | (5) vs. (1) | $779 |
| Bell, J., 1997 USD | (1) 6 INH; (2) 3 RIF INH; (3) 2 RIF PZA (4) No preventative therapy | TB medical care and social costs (transport, lodging, food, time spent away from normal activities) (per person) | 1 | $73.81 | Primary Active TB Cases (per 100,000) | 1 | 30,020 | Incremental cost (medical and social including secondary cases) per active TB case averted | (1) vs. (4) | Cost-saving |
|  |  |  | 2 | $91.61 |  | 2 | 30,913 |  | (2) vs. (4) | $70.98 |
|  |  |  | 3 | $90.90 |  | 3 | 29,246 |  | (3) vs. (4) | $91.89 |
|  |  |  | 4 | $86.53 |  | 4 | 38,126 |  |  |  |
| Burgos, J., 2007 USD | (1) 6 INH; (2) No LTBI program | Total cost per 1000 individuals with high HIV & TB risk (HIV risk: 4/100 py, TB risk: 1.1/100 py) | 1 | $1,839,706 | Active TB Cases (among high risk TB and HIV population) | 1 | 177 | Incremental cost per TB death averted | (1) vs. (2) | $737 |
|  |  |  |  |  |  | 2 | 225 |  |  |  |
|  |  |  | 2 | $1,991,310 | TB Deaths (among high risk TB and HIV population) | 1 | 25 | Incremental cost per LTBI case detected |  | $730 |
|  |  |  |  |  |  | 2 | 80 | Incremental cost per active TB case prevented |  | $529 |
| Currie, C., 2003 USD | (1) 6 INH; (2) Lifetime INH; (3) Baseline - no TPT | Total annual cost (in millions) | 1 | $82,800,000 | Annual Deaths Averted vs. Baseline Scenario (baseline 774,000 deaths and 225,000 active TB cases over 20 years) | 1 | 549 | Incremental cost per QALY gained | (1) vs. (3) | $84.70 |
|  |  |  |  |  |  | 2 | 2075 |  | (2) vs. (3) | $373 |
|  |  |  | 2 | $89,700,000 |  | 3 | Reference |  |  |  |
|  |  |  |  |  | Annual Active TB Cases Averted vs. Baseline Scenario (baseline 774,000 deaths and 225,000 active TB cases over 20 years) | 1 | 2119 | Incremental cost per death averted | (1) vs. (3) | $1,136 |
|  |  |  | 3 | $82,200,000 |  | 2 | 5480 |  | (2) vs. (3) | $3,360 |
|  |  |  |  |  |  | 3 | Reference |  |  |  |
| de Siqueira Filha, N., 2015 USD | (1) PLHIV individuals diagnosed and treated for LTBI (12 INH); (2) PLHIV individuals diagnosed and treated for active TB | Total costs per patient (direct and indirect) during treatment period | 1 | $127.20 (p value 0.0601) |  | | |  | | |
|  |  |  | 2 | $839.80 (p value 0.0601) |  |  |  |  |  |  |
|  |  | Total indirect costs per patient during treatment period (income loss, time loss) | 1 | $35.90 (p value 0.032) |  |  |  |  |  |  |
|  |  |  | 2 | $613.70 (p value 0.032) |  |  |  |  |  |  |
| de Siqueira Filha, N., 2015 USD | (1) PLHIV individuals diagnosed and treated for LTBI (12 INH); (2) PLHIV individuals diagnosed and treated for active TB | Mean total inpatient cost per patient (medical and non-medical) during treatment period | 1 | $549.50 |  | | |  | | |
|  |  |  | 2 | $4,371.70 (SD $3,652.70) |  |  |  |  |  |  |
|  |  | Mean total outpatient cost per patient (medical and non-medical) during treatment period | 1 | $609.30 (SD $383.40) |  |  |  |  |  |  |
|  |  |  | 2 | $687.30 (SD $406.30) |  |  |  |  |  |  |
| Ferguson, O., 2019 USD | (1) 1 INH RPT delivered to patients in an HIV clinic in Uganda (rate of treatment completion is 94%; (2) 3 INH RPT delivered to patients in an HIV clinic in Uganda (rate of treatment completion is 74%) | Total cost per 1000 individuals | 1 | $1,528,168 | Number of cases of reactivated TB per 1000 individuals | 1 | 11.1 | Incremental cost per DALY averted | (1) vs. (2) | $1,221 |
|  |  |  |  |  |  | 2 | 21.3 |  |  |  |
|  |  |  | 2 | $1,522,009 | Number of DALYs per 1000 individuals | 1 | 7391.7 | Incremental cost per reactivated TB case averted (calculated) |  | $603.82 |
|  |  |  |  |  |  | 2 | 7396.7 |  |  |  |
| Foster, S., 1991 UK £ | (1) Base case with voluntary testing and counselling (VTC) site for HIV, TPT is suggested; (2) Referral from VTC to a TB clinic to start TPT; (3) Referral from STD clinic to TB clinic to start TPT; (4) TPT is offered to large numbers of people based on occupation or residence | Cost of TPT | 1 | 4011 | TB cases prevented (index + additional), assuming that preventing 1 case of TB prevents an additional 2 cases | 1 | 51 | Incremental cost per TB case prevented | (2) vs. (1) | Cost saving |
|  |  |  | 2 | 3492 |  | 2 | 192 |  | (3) vs. (1) | Cost saving |
|  |  |  | 3 | 3577 |  | 3 | 52 |  | (4) vs. (1) | Cost saving |
|  |  |  | 4 | 3492 |  | 4 | 226 |  |  |  |
| Gilbert, J. A., 2015 USD | (1) Status quo with 36 INH for TST+ and 12 INH for TST- ("36/12 INH"); (2) Status quo with lifetime INH for all; (3) Community TB/HIV screening every 2 years with 36/12 INH; (4) Community TB/HIV screening every 2 years with lifetime INH for all | Discounted lifetime costs | 1 | $225,249,000 | Total TB cases (out of a community of 90,000) | 1 | 4189 | Incremental cost per TB case averted | (2) vs. (1) | $16,316 |
|  |  |  |  |  |  | 2 | 3718 |  | (3) vs. (1) | $65,528 |
|  |  |  | 2 | $232,934,000 |  | 3 | 3795 |  | (4) vs. (1) | $39,393 |
|  |  |  |  |  |  | 4 | 3167 |  |  |  |
|  |  |  | 3 | $251,067,000 | Total HIV cases (out of a community of 90,000) | 1 | 8359 | Incremental cost per HIV case averted | (2) vs. (1) | Dominated |
|  |  |  |  |  |  | 2 | 8368 |  | (3) vs. (1) | $52,052 |
|  |  |  | 4 | $265,509,000 |  | 3 | 7863 |  | (4) vs. (1) | $56,072 |
|  |  |  |  |  |  | 4 | 7641 |  |  |  |
| Gourevitch, M., 1998 USD | (1) 0% efficacy of 12 INH; (2) 40% efficacy of 12 INH (3) 65% efficacy of 12 INH; (4) 90% efficacy of 12 INH | Associated hospital costs after 5 years of follow-up of 507 persons who are HIV-seropositive, PPD positive | 1 | $307,422 | Expected number of TST-positive TB cases after 5 years of follow-up of 507 persons among PLHIV | 1 | 8 | Net savings per person receiving chemoprophylaxis (combined result for PLHIV and HIV-) | (2) vs. (1) | $1,289 |
|  |  |  | 2 | $192,139 |  | 2 | 5 |  | (3) vs. (1) | $2,226 |
|  |  |  | 3 | $115,283 |  | 3 | 3 |  | (4) vs. (1) | $3,148 |
|  |  |  | 4 | $38,428 |  | 4 | 1 | Program costs per TB case prevented | (3) vs. (1) | $10,274 |
|  |  | Associated hospital costs after 5 years of follow-up of 507 persons who are HIV-seropositive, anergic | 1 | $268,994 | Expected number of anergic TB cases after 5 years of follow-up of 507 persons among PLHIV | 1 | 7 |  |  |  |
|  |  |  | 2 | $153,711 |  | 2 | 4 |  |  |  |
|  |  |  | 3 | $115,283 |  | 3 | 3 |  |  |  |
|  |  |  | 4 | $38,428 |  | 4 | 1 |  |  |  |
| Gupta, S., 2010 USD | (1) 90% coverage of ART (ARTexp), TB infection control in hospitals (IC), intensified TB case finding (ICF), 36 INH; (2) ARTexp ICF 36 INH; (3) ART IC ICF 36 INH; (4) ARTexp IC ICF 6 INH TST 36 INH | Total cost with TB diagnostic algorithm: sputum smear and chest radiography | 1 | $6,580,182 | Total TB cases with TB diagnostic algorithm: sputum smear and chest radiography | 1 | 791 | Incremental cost per TB case averted | (1) vs. (2) | $196 |
|  |  |  | 2 | $6,571,933 |  | 2 | 833 |  | (2) vs. (3) | $294,099 |
|  |  |  | 3 | $5,101,438 |  | 3 | 838 |  | (3) vs. (4) | -$69,066 |
|  |  |  | 4 | $6,344,626 |  | 4 | 856 |  |  |  |
| Hausler, H. P., 2002 USD | (1) Voluntary counselling and testing; (2) Intensified case finding; (3) 6 INH; (4) No intervention | Community health centre: cost per person | 1 | $9 |  | | | Community health centre: cost per TB case averted | (1) vs. (4) | $178 |
|  |  |  | 2 | $166 |  |  |  |  | (2) vs. (4) | $664 |
|  |  |  | 3 | $110 |  |  |  |  | (3) vs. (4) | $962 |
| Hseih, Y.,  2017 USD | (1) No TPT; (2) 6 INH; (3) Lifetime INH (with an expected duration of 28 months). Strategies (2) and (3) occur under a budgetary constraint of $10.8 million |  | | | Active TB incidence per 100,000 in 2030 (baseline incidence is 246.2 per 100,000 in 2018) | 1 | 137 | Incremental cost per TB case averted | (2) vs. (1) | $270 ($175 - $382) |
|  |  |  |  |  |  | 2 | 127 |  |  |  |
|  |  |  |  |  |  | 3 | 125 |  |  |  |
|  |  |  |  |  | Number of incident TB cases averted among those 15 years and older in 2030 | (2) vs. (1) | 20,033 |  | (3) vs. (1) | $1029 ($778 - $1446) |
|  |  |  |  |  |  | (3) vs. (1) | 22,500 |  |  |  |
|  |  |  |  |  | Number of TB deaths averted | (2) vs. (1) | 9,591 |  |  |  |
|  |  |  |  |  |  | (3) vs. (1) | 10,037 |  |  |  |
| Jo, Y.,  2018 USD | (1) TTT* for PLHIV in California  (2) No TTT for PLHIV in California  (3) TTT for PLHIV in Florida  (4) No TTT for PLHIV in Florida  *2 more regions included in study but results not shown here*  TTT = targeted testing and treatment; tested with IGRA and treated with 3 INH RPT | Cost of self-administered TPT (10,532 individuals completed TPT in California; 4,116 individuals completed TPT in Florida) | 1 | $7,270,696 | Number of TB cases averted | (1) vs. (2) | 669 | Incremental cost per TB case averted | (1) vs. (2) | $9,323 |
|  |  |  | 2 | $0 |  |  |  |  |  |  |
|  |  |  | 3 | $2,546,809 |  | (3) vs. (4) | 739 |  | (3) vs. (4) | $4,428 |
|  |  |  | 4 | $0 |  |  |  |  |  |  |
|  |  | Total intervention cost | 1 | $19,627,676 | QALYs gained due to averted TB | (1) vs. (2) | 951 | Incremental cost per QALY gained | (1) vs. (2) | $6,695 |
|  |  |  | 2 | $0 |  |  |  |  |  |  |
|  |  |  | 3 | $12,180,219 |  | (3) vs. (4) | 1,154 |  | (3) vs. (4) | $2,828 |
|  |  |  | 4 | $0 |  |  |  |  |  |  |
| Johnson, K., 2017 USD | (1) 3 INH RPT; (2) 9 INH; (3) No TPT | Cost of ART per 1000 individuals | 1 | $2,431,000 | Active TB Cases | 1 | 28 | Incremental cost per DALY averted | (1) vs. (3) | $272 |
|  |  |  | 2 | $2,432,000 |  | 2 | 37 |  | (2) vs. (3) | $266 |
|  |  | Cost of TPT per 1000 individuals | 1 | $3,430 | All-Cause Deaths | 1 | 465 |  | (1) vs. (2) | $9,402 |
|  |  |  | 2 | $59,333 |  | 2 | 466 | Incremental cost per TB case averted | (1) vs. (2) | Dominated |
|  |  | Cost of active TB treatment per 1000 individuals | 1 | $8,048 | TB-Specific DALYs | 1 | 10,837 |  |  |  |
|  |  |  | 2 | $6,016 |  | 2 | 10,843 |  |  |  |
| Kapoor, S., 2014 USD | (1) 6 INH for all patients; (2) 6 INH for patients with CD4 counts less than or equal to 200 cells/mL; (3) Standard of care - no TPT | Net cost per individual | 1 | $22.53 ($15.02 - $69.27) | DALYs per individual | 1 | 20.287 (20.105 - 23.614) | Incremental cost per DALY averted | (1) vs. (3) | $178 ($23 - $1309) |
|  |  |  |  |  |  | 2 | 20.391 (20.245 - 20.863) |  | (2) vs. (3) | $201 ($19 - $1197) |
|  |  |  |  |  |  | 3 | 20.401 (2.284 - 23.837) |  | (1) vs. (2) | $176 (-$309 - $343) |
|  |  |  | 2 | $4.28 ($3.76 - $35.02) | Active TB cases per 1000 patients | 1 | 16 (9 - 100) | Incremental cost per TB case averted | (1) vs. (3) | $965 |
|  |  |  |  |  |  | 2 | 34 (17 - 150) |  | (2) vs. (3) | $670 |
|  |  |  |  |  |  | 3 | 37 (20 - 184) |  | (1) vs. (2) | $1,014 |
| Kim, H-Y., 2016 USD | (1) 12 INH indicated by TPT (12); (2) 12 INH indicated by QGIT; (3) 12 INH for all | Average cost per patient for diagnostic testing | 1 | $3.90 | Active TB Cases per 1000 patients | 1 | 24.5 | Incremental cost per DALY averted | (1) vs. (3) | $640 |
|  |  |  | 2 | $58.11 |  | 2 | 22.6 |  |  |  |
|  |  |  | 3 | $0.23 |  | 3 | 21 |  |  |  |
|  |  | Average cost per patient for TPT | 1 | $2.79 | DALYs | 1 | 177 |  |  |  |
|  |  |  | 2 | $3.92 |  | 2 | 171 |  |  |  |
|  |  |  | 3 | $8.29 |  | 3 | 168 |  |  |  |
| Kowada, A., 2012 USD | (1) 6 INH indicated by T-SPOT; (2) 6 INH indicated by QFT; (3) 6 INH indicated by TST; (4) TST followed by T-SPOT directed 6 INH | Cost for PLHIV pregnant women in close contacts (Non-BCG vaccinated cohort during pregnancy) | 1 | $8,917.53 | QALYs for PLHIV pregnant women in close contacts (Non-BCG vaccinated cohort during pregnancy) | 1 | 20.82595 | Incremental cost per QALY gained (in close contacts) | (2), (3), or (4) vs. (1) | Dominated |
|  |  |  | 2 | $8,983.62 |  | 2 | 20.82516 |  |  |  |
|  |  |  | 3 | $9,513.82 |  | 3 | 20.8189 |  |  |  |
|  |  |  | 4 | $9,837.47 |  | 4 | 20.81856 |  |  |  |
| Linas, B., 2011 USD | (1) No screening; (2) Using TST to screen; (3) Using IGRA to screen, all three of which are followed by 9 INH | Discounted per person lifetime cost | 1 | $503,300 | Undiscounted per person life expectancy | 1 | 516.72 | Incremental cost per QALY gained | (2) vs. (1) | $12,800 |
|  |  |  |  |  |  | 2 | 516.93 |  |  |  |
|  |  |  | 2 | $503,420 |  | 3 | 516.95 |  |  |  |
|  |  |  |  |  | Discounted per person quality-adjusted life expectancy | 1 | 279.55 |  | (3) vs. (1) | $23,800 |
|  |  |  | 3 | $503,440 |  | 2 | 279.67 |  |  |  |
|  |  |  |  |  |  | 3 | 279.68 |  |  |  |
| Maheswaran, H., 2010 USD | 1. Presence of chronic cough; (2) Any classic symptom; (3) Any classic symptom followed by CXR; (4) Any classic symptom followed by sputum (all followed by TPT) | Mean cost per individual | 1 | $221 | Mean QALYs per individual | 1 | 1.137 | Incremental cost per QALY gained | (1) vs. (4) | Dominated |
|  |  |  | 2 | $325 |  | 2 | 1.147 |  |  |  |
|  |  |  | 3 | $191 |  | 3 | 1.136 |  |  |  |
|  |  |  | 4 | $221 |  | 4 | 1.142 |  |  |  |
| Masobe, P., 1993 SA Rands | (1) 6 INH; (2) No TPT | Cumulative total discounted cost | 1 | R51,333,954 | Active TB cases over 8 years | 1 | 12,000 | Incremental cost per TB case averted | (1) vs. (2) | $1,844 |
|  |  |  | 2 | R91,904,352 |  | 2 | 34,000 |  |  |  |
| Perlman, D. C., 1996 USD | (1) 9 INH with 65% efficacy; (2) No TPT | Cost per person of INH chemoprophylaxis under direct observation and of associated clinical monitoring | 1 | $768.59 | Cases of active tuberculosis over 5 years of follow up among PLHIV (with anergy) | 1 | 0 | Cost per active TB case averted | (1) vs. (2) | Dominated (i.e. IPT is cost-saving) |
|  |  |  | 2 | $0 |  |  |  |  |  |  |
|  |  | Hospital costs associated with active tuberculosis cases occuring over 5 years of follow up for PLHIV (with anergy) | 1 | $0 |  | 2 | 1 |  |  |  |
|  |  |  | 2 | $38,428 |  |  |  |  |  |  |
| Pho, M., 2009 USD | (1) 6 EMB INH; (2) 36 INH; (3) No TPT | Discounted mean per person cost | 1 | $2,820 | TB cases per 100 person-years | 1 | 3.62 | Incremental cost per life-month gained | (1) vs. (3) | $1,490 |
|  |  |  | 2 | $2,870 |  | 2 | 3.44 |  | (2) vs. (3) | $3,120 |
|  |  |  | 3 | $2,740 |  | 3 | 4.47 |  |  |  |
|  |  | Discounted mean per person lifetime cost | 1 | $5,730 | Discounted mean person life expectancy, months | 1 | 136.9 | Incremental cost per TB case averted | (1) vs. (3) | $9,412 |
|  |  |  | 2 | $5,780 |  | 2 | 137.1 |  | (2) vs. (3) | $12,621 |
|  |  |  | 3 | $5,630 |  | 3 | 136.1 |  |  |  |
| Samandari, T., 2008 USD | (1) Symptom screening before 6 INH; (2) Symptom screening + chest x-ray before 6 INH; (3) Symptom screening + chest x-ray + active TB patient tracking before 6 INH | Total cost per 10,000 people over 3 years (including INH) | 1 | $395,100 | New INH-R TB disease per 10,000 HIV-infected adults over 3 years | 1 | 21.63 | Incremental cost per INH-R TB cases averted | (2) vs. (1) | $7,933 |
|  |  |  |  |  |  | 2 | 5.61 |  | (3) vs. (1) | $14,368 |
|  |  |  |  |  |  | 3 | 6.84 | Incremental cost per death averted | (2) vs. (1) | Dominated |
|  |  |  | 2 | $522,300 | New deaths per 10,000 HIV-infected adults over 3 years | 1 | 116.53 |  | (3) vs. (1) | $2,816,061 |
|  |  |  |  |  |  | 2 | 131.11 |  |  |  |
|  |  |  |  |  |  | 3 | 116.45 |  |  |  |
| Sawert, H., 1997 USD | (1) INH for TST+; (2) INH for TST+ and those with CD4+ cell counts < 0.20x10^9/L; (3) INH for TST+ and those with CD4+ cell counts < 0.35x10^9/L; (4) INH for TST+ and all anergic cohort members | Marginal provider cost in millions, relative to no IPT, which is $61.9M ($57.8M - $66.1M) | 1 | -$7,700,000 (-$6,500,000 - $9,000,000) | QALYs relative to no IPT, which has 313,475 QALYs | 1 | 1151 (1026 - 1245) | Incremental cost per QALY gained | (2) vs. (1) | $578 ($1 - $1786) |
|  |  |  | 2 | -$800,000 (-$2,300,000 - $500,000) |  | 2 | 1057 (874 - 1163) |  |  |  |
|  |  |  | 3 | -$2,200,000 (-$1,300,000 - $3,300,000) |  | 3 | 528 (386 - 593) |  |  |  |
|  |  |  | 4 | -$1,700,000 (-$700,000 - $2,700,000) |  | 4 | 460 (245 - 539) |  |  |  |
| Shayo, G., 2012 USD | (1) 6 INH; (2) No TPT | Clinic personnel costs | 1 | $1,248,668 | TB incidence density/100,000 PY | 1 | 91 | Incremental cost per TB case averted | (1) vs. (2) | $405.93 |
|  |  |  | 2 | $1,177,786 |  | 2 | 511 |  |  |  |
|  |  | Laboratory test costs | 1 | $677 | Mortality incidence density/100,000 PY | 1 | 136 | Incremental cost per death averted | (1) vs. (2) | $174.15 |
|  |  |  | 2 | $619 |  | 2 | 1115 |  |  |  |
| Shrestha. R., 2003 USD | (1) Using TST prior to provision of 9 INH; (2) Not using TST prior to provision of 9 INH; (3) Using TST prior to provision of 6 INH; (4) Not using TST prior to provision of 6 INH; (5) No IPT | Program cost per 100 patients | 1 | $1,980 | Cases treated per 100 patients | 1 | 9.4 | Incremental cost per TB case treated | (1) vs. (5) | $210.64 |
|  |  |  | 2 | $5,130 |  | 2 | 13.5 |  | (2) vs. (5) | $768.29 |
|  |  |  | 3 | $1,650 |  | 3 | 10.5 |  | (3) vs. (5) | $157.14 |
|  |  |  | 4 | $3,870 |  | 4 | 15.1 |  | (4) vs. (5) | $482.61 |
|  |  |  | 5 | $0 |  | 5 | 0 |  |  |  |
| Shrestha. R., 2003 USD | (1) 9 INH for all; (2) Indicated 9 INH with TST; (3) 6 INH for all; (4) Indicated 6 INH with TST; (5) No IPT | Expected cost per 100 patients | 1 | $13,360 | Expected QALYs per 100 clients | 1 | 539 | Incremental cost per QALY gained | (2) vs. (5) | $102 |
|  |  |  | 2 | $10,160 |  | 2 | 509 |  |  |  |
|  |  |  | 3 | NR |  | 3 | 535 |  | (2) vs. (1) | $106 |
|  |  |  | 4 | NR |  | 4 | 507 |  |  |  |
|  |  |  | 5 | $9,060 |  | 5 | 498 |  |  |  |
| Smith, T., 2010 USD | (1) Provide all PLHIV 6 INH and initiate ART at CD4 < 250 cells/mL; (2) Provide all PLHIV with 36 INH and initiate ART at CD4 < 250 cells/mL; (3) Provide only TST-positive PLHIV with 6 INH and initiate ART at CD4 < 250 cells/mL; (4) Provide only TST-positive PLHIV with 36 INH and initiate ART at CD4 < 250 cells/mL; (5) Provide all PLHIV with 6 INH, and 36 INH for TST-positives, and initiate ART at CD4 < 250cells/mL | Total cost of program | 1 | $5,937,863 | Active TB Cases | 1 | NR | Incremental cost per TB case averted | (4) vs. (3) | $1,612 |
|  |  |  |  |  |  | 2 | NR |  |  |  |
|  |  |  | 2 | Dominated |  | 3 | 318 |  | (5) vs. (4) | $6,549 |
|  |  |  |  |  |  | 4 | 198 |  |  |  |
|  |  |  | 3 | $5,874,660 |  | 5 | 171 |  |  |  |
|  |  |  |  |  | All-Cause Deaths | 1 | NR | Incremental cost per death averted | (4) vs. (3) | $2,148 |
|  |  |  | 4 | $6,068,082 |  | 2 | NR |  |  |  |
|  |  |  |  |  |  | 3 | 301 |  | (5) vs. (4) | $58,944 |
|  |  |  | 5 | $6,244,913 |  | 4 | 221 |  |  |  |
|  |  |  |  |  |  | 5 | 218 |  |  |  |
| Snyder, D. C., 1998 USD | (1) TB screening and 12 INH; (2) No TPT | Total program cost (January 1990 to December 1995) per person enrolled | 1 | $287 | TB cases occurring over 10 years for those with and without HIV infection (19% of the cohort was PLHIV) | 1 | 27.7 | Net average savings per TB case prevented (the total program costs were less than the costs associated with managing active TB cases that would've occurred otherwise) | (1) vs. (2) | $3,724 |
|  |  |  | 2 | $0 |  | 2 | 57.7 |  |  |  |
| Sutton, B., 2008 USD | (1) Intensified case finding (ICF); (2) 9 INH | Cost per person | 1 | $24.08 |  | | | | | |
|  |  |  | 2 | $130.51 |  |  |  |  |  |  |
|  |  | Cost per TB case prevented | 1 | $363.06 |  |  |  |  |  |  |
|  |  |  | 2 | $955.33 |  |  |  |  |  |  |
| Tasillo, A., 2015 USD | (1) No testing and no TPT; (2) Confirm positive (those with positive TST given IGRA, and if still positive, then given 3 INH RPT); (3) Postive TST followed by 3 INH RPT; (4) Positive IGRA followed by 3 INH RPT; (5) Confirm negative (those with negative IGRA given TST, either with positive result given 3 INH RPT) | Incremental cost per person compared to preceding strategy for PLHIV cohort | 1 | NA | Incremental QALY per person compared to preceding strategy for PLHIV | 1 | NA | Incremental cost per QALY gained | (2) vs. (1) | $18,000 |
|  |  |  | 2 | $58 |  | 2 | 0.0032 |  | (3) vs. (2) | Dominated |
|  |  |  | 3 | $39 |  | 3 | 0.001 |  | (4) vs. (3) | $35,000 |
|  |  |  | 4 | $55 |  | 4 | 0.0017 |  | (5) vs. (4) | $63,000 |
|  |  |  | 5 | $50 |  | 5 | 0.0008 |  |  |  |
| Terris-Prestholt, F., 2007 USD | (1) ProTEST Initiative in the community of Chawama; (2) ProTEST Initiative in the community of Matero (ProTEST includes 6 INH) | Total annual cost of HIV clinic | 1 | $21,132 |  | | | | | |
|  |  |  | 2 | $15,991 |  |  |  |  |  |  |
|  |  | Total annual cost of voluntary counselling and testing centre | 1 | $47,598 |  |  |  |  |  |  |
|  |  |  | 2 | $8,532 |  |  |  |  |  |  |
|  |  | Total annual cost of TPT | 1 | $1,373 |  |  |  |  |  |  |
|  |  |  | 2 | $1,300 |  |  |  |  |  |  |

*TB = tuberculosis, HIV = human immunodeficiency virus, TPT = tuberculosis preventive therapy, LTBI = latent tuberculosis infection, PLHIV = people living with HIV, INH = isoniazid, TST = tuberculin skin test, IGRA = interferon gamma release assay, RPT = rifapentine, RIF = rifampin, AIDS = acquired immunodeficiency syndrome, DALY = disability adjusted life year, QALY = quality adjusted life year, CPT = cotrimoxazole preventive therapy, BCG = Bacillus Calmette–Guérin (vaccine), EMB = ethambutol, ART = antiretroviral therapy, CD4 = cluster of differentiation 4 (type of glycoprotein)*

**Cost values listed are in currency of the study**

* In some cases, studies compared more strategies and had more modelled outcomes than are listed in this table

## Table J. Detailed outcomes of studies that reported effectiveness outcomes only

| **Study Author** | **Strategies Compared*** | **Effectiveness Outcomes** | | |
| --- | --- | --- | --- | --- |
|  |  | **Outcome** | **Strategy** | **Value**  **(95% CI))** |
| Bacaer, N. | (1) No intervention; (2) IPT; (3) ART | TB notification rate per 100,000 per year in 1980 vs. 2080 | 1 | 208 vs. 2002 |
|  |  |  | 2 | 208 vs. 1053 |
|  |  |  | 3 | 208 vs. 886 |
|  |  | HIV prevalence in 1980 vs. 2080 | 1 | 0% vs. 23% |
|  |  |  | 2 | NA |
|  |  |  | 3 | 0% vs. 36% |
| Basu, S. | (1) No TST, 60% sensitivity of active TB screening, then INH; (2) No TST, 90% sensitivity of screening, then INH; (3) TST, 60% sensitivity of screening, then INH; (4) TST, 90% sensitivity of screening, then INH | Change in TB incidence per 100,000 (among PLHIV) | 1 | -21 (-42 - 0) |
|  |  |  | 2 | -22 (-43 - 0) |
|  |  |  | 3 | -14 (-25 - 0) |
|  |  |  | 4 | -14 (-20 - 0) |
|  |  | Change in TB prevalence per 100,000 (among PLHIV) | 1 | -11 (-9 - 0) |
|  |  |  | 2 | -11 (-10 - 0) |
|  |  |  | 3 | -7 (-5 - 0) |
|  |  |  | 4 | -7 (-4 - 0) |
|  |  | Change in % INH resistant (among PLHIV) | 1 | 19 (7 - 28) |
|  |  |  | 2 | 16 (8 - 29) |
|  |  |  | 3 | 19 (12 - 32) |
|  |  |  | 4 | 16 (8 - 29) |
| Brewer, T. | (1) 10% increase in access to current TPT; (2) BCG vaccination of 10% of the eligible population | Percent decline in active TB cases among chronically homeless individuals with AIDS | 1 | 8.90% |
|  |  |  | 2 | 1.00% |
|  |  | Percent decline in TB deaths among chronically homeless individuals with AIDS | 1 | 4.80% |
|  |  |  | 2 | 0.20% |
|  |  | Percent decline in active TB cases among transiently homeless individuals with AIDS | 1 | 10.90% |
|  |  |  | 2 | 1.20% |
|  |  | Percent decline in TB deaths among transiently homeless individuals with AIDS | 1 | 7.80% |
|  |  |  | 2 | 0.00% |
| Brewer, T. (1996) | (1) 10% increase in INH coverage; (2) 10% increase in INH efficacy; (3) 10% increase in TB treatment coverage; (4) 10% increase in TB treatment efficacy | Percentage difference in total number of TB cases after 10 years (among individuals with AIDS) | 1 | -34% |
|  |  |  | 2 | -6.2% |
|  |  |  | 3 | 70% (all drug resistant) |
|  |  |  | 4 | -30% |
|  |  | Reduction in number of deaths due to TB over 10 years (among individuals with AIDS) | 1 | -18 |
|  |  |  | 2 | -3 |
|  |  |  | 3 | -1 |
|  |  |  | 4 | -5 |
| Cohen, T. | (1) 0% INH coverage; (2) 33% INH coverage; (3) 66% INH coverage; (4) 99% INH coverage (in all four scenarios, duration of INH varies, but isn’t specified) | TB prevalence per 100,000 people over 5 years | 1 | 779 |
|  |  |  | 2 | 675 |
|  |  |  | 3 | 496 |
|  |  |  | 4 | 190 |
|  |  | Proportion of population with latent infection, % over 5 years | 1 | 37.4 |
|  |  |  | 2 | 34.1 |
|  |  |  | 3 | 28.6 |
|  |  |  | 4 | 19.8 |
| Dowdy, D. | (1) Continuous delivery of TST screening and 6 INH to 20% of the eligible population; (2) Usual care | % reduction in TB incidence among PLHIV | 1 | 15.6 (15.5 - 36.5) |
|  |  |  | 2 | Reference |
|  |  | % reduction in TB mortality among PLHIV | 1 | 14.3 (14.6 - 33.7) |
|  |  |  | 2 | Reference |
| Dye, C. | (1) Current program; (2) ART for PLHIV; (3) Treat latent TB with INH in PLHIV (or treat latent TB regardless of HIV status in India and China) | South Africa: TB cases per million per year in 1990 vs. 2050 | 1 | 5286 to 8429 |
|  |  |  | 2 | 5286 to 8285 |
|  |  |  | 3 | 5286 to 2286 |
|  |  | India: TB cases per million per year in 1990 vs. 2050 | 1 | 2248 to 1532 |
|  |  |  | 2 | NA |
|  |  |  | 3 | 2248 to 1 |
|  |  | China: TB cases per million per year in 1990 vs. 2050 | 1 | 2955 to 405 |
|  |  |  | 2 | NA |
|  |  |  | 3 | 2955 to 1 |
| Freiman, J. | (1) ART alone; (2) ART with 6 INH; (3) ART with 36 INH | Brazil - active TB cases per 1000 persons | 1 | 259 (142 - 374) |
|  |  |  | 2 | 209 (110 - 312) |
|  |  |  | 3 | 193 (99 - 293) |
|  |  | India - active TB cases per 1000 persons | 1 | 801 (594 - 908) |
|  |  |  | 2 | 706 (487 - 841) |
|  |  |  | 3 | 665 (453 - 809) |
|  |  | Uganda - active TB cases per 1000 persons | 1 | 418 (248 - 577) |
|  |  |  | 2 | 336 (194 - 503) |
|  |  |  | 3 | 308 (172 - 468) |
| Guwatudde, D. | (1) 0% coverage of TPT (drug regimen not specified); (2) 25% coverage of TPT; (3) 50% coverage of TPT; (4) 75% coverage of TPT; (5) 100% coverage of TPT | Number of TB deaths over 20 years | 1 | 214,525 |
|  |  |  | 2 | 202,782 |
|  |  |  | 3 | 191,092 |
|  |  |  | 4 | 179,456 |
|  |  |  | 5 | 167,874 |
| Heymann, S. J. | (1) No one received INH; (2) 10% of the population received INH; (3) 30% of the population received INH (in this population, prevalence of HIV is 20%, with an annual increase of 2.5%) | Percent of original population with new TB infection after 10 years | 1 | 30% |
|  |  |  | 2 | 11.25% |
|  |  |  | 3 | 0.75% |
|  |  | Percent of original population with active TB after 10 years | 1 | 1% |
|  |  |  | 2 | 0.50% |
|  |  |  | 3 | 0% |
| Houben, R. | (1) 6 INH; (2) 12 INH RPT; (3) 3 INH RIF; (4) 3 INH RIF PZA; (5) Placebo | Kenya - Estimated annual risk of infection, %/year | 1 | 4.9 (3 - 12.2) |
|  |  |  | 2 | NA |
|  |  |  | 3 | NA |
|  |  |  | 4 | 3.7 (2.9 - 5.1) |
|  |  |  | 5 | 4.9 (3 - 12.2) |
|  |  | Uganda - Estimated annual risk of infection, %/year | 1 | 3.7 (2.9 - 5.1) |
|  |  |  | 2 | NA |
|  |  |  | 3 | NA |
|  |  |  | 4 | NA |
|  |  |  | 5 | 3.7 (2.9 - 5.1) |
|  |  | South Africa - Estimated annual risk of infection, %/year | 1 | 3.6 |
|  |  |  | 2 | 3.6 |
|  |  |  | 3 | 3.6 |
|  |  |  | 4 | NA |
|  |  |  | 5 | NA |
| Jordan, T. J. | (1) IPT; (2) No IPT | Life expectancy for black men, unknown TST status | 1 | 9.12 yrs |
|  |  |  | 2 | 8.90 yrs |
|  |  | Life expectancy for black women, unknown TST status | 1 | 9.07 yrs |
|  |  |  | 2 | 8.90 yrs |
|  |  | Life expectancy for white men, unknown TST status | 1 | 9.18 yrs |
|  |  |  | 2 | 9.04 yrs |
|  |  | Life expectancy for white women, unknown TST status | 1 | 9.19 yrs |
|  |  |  | 2 | 9.04 yrs |
|  |  | Life expectancy for black men, TST+ | 1 | 8.80 yrs |
|  |  |  | 2 | 8.02 yrs |
|  |  | Life expectancy for black women, TST+ | 1 | 8.76 yrs |
|  |  |  | 2 | 8.02 yrs |
|  |  | Life expectancy for white men, TST+ | 1 | 8.97 yrs |
|  |  |  | 2 | 8.45 yrs |
|  |  | Life expectancy for white women, TST+ | 1 | 8.97 yrs |
|  |  |  | 2 | 8.45 yrs |
| Kendall, E. | (1) 12 INH regimen linked with ART; (2) Lifetime INH linked with ART | Total number of people treated with IPT, thousands after 5 years | 1 | 21.3 (17.9 - 26) |
|  |  |  | 2 | 20.8 (17.3 - 25.5) |
|  |  | Cumulative TB cases averted, thousands, over 5 years | 1 | 1.1 (0.7 - 1.9) |
|  |  |  | 2 | 2 (1.3 - 2.8) |
|  |  | Cumulative TB deaths averted, thousands, over 5 years | 1 | 0.14 (0.07 - 0.24) |
|  |  |  | 2 | 0.22 (0.11 - 0.36) |
| Kunkel, A. | (1) Lifetime INH with no dropout; (2) Realistic continuous INH accounting for dropout (median duration 4.7 years); (3) 6 INH; (4) No IPT | Drug sensitive TB incidence per 100,000 from 2020-2050 | 1 | 283 to 80 |
|  |  |  | 2 | 283 to 103 |
|  |  |  | 3 | 283 to 130 |
|  |  |  | 4 | 283 to 148 |
|  |  | Isoniazid monoresistant TB incidence per 100,000 from 2020-2050 | 1 | 22.8 to 23.2 |
|  |  |  | 2 | 22.8 to 25.3 |
|  |  |  | 3 | 22.8 to 29.4 |
|  |  |  | 4 | 22.8 to 32.2 |
| Long, E. | (1) 100% combination treatment (HAART, active TB treatment, LTBI treatment with INH – duration not specified); (2) 50% combination treatment (HAART, active TB treatment, INH); (3) 100% INH; (4) 50% INH; (5) No treatment | New HIV cases (millions) over 20 years | 1 | 21.88 |
|  |  |  | 2 | 24.51 |
|  |  |  | 3 | 32.04 |
|  |  |  | 4 | 26.85 |
|  |  |  | 5 | 22.91 |
|  |  | New active TB cases (millions) over 20 years | 1 | 0 |
|  |  |  | 2 | 6.23 |
|  |  |  | 3 | 0 |
|  |  |  | 4 | 6.53 |
|  |  |  | 5 | 13.55 |
| Mandal, S. | (1) Status quo (no 3 INH RPT-like regimen modelled)  (2) 3 INH RPT-like regimen scaled up in each country (11 countries included) over a course of 3 years  (3) Improved TB cascade plus 3 INH RPT-like regimen | Annual TB incidence per 100,000 in 2030 (baseline = 218 per 100,000 in 2019) | 1 | 196 |
|  |  |  | 2 | 173 |
|  |  |  | 3 | 129 |
|  |  | Number of annual TB deaths per 100,000 in 2030 (baseline = 6.2 per 100,000 in 2019) | 1 | 5.5 |
|  |  |  | 2 | 4.9 |
|  |  |  | 3 | 2.5 |
| Marx, F. M. | (1) Targetted active case finding only; (2) Targeted active case finding + secondary lifetime INH; (3) Base case with no strategy (strategies targeted to people who previously completed TB treatment) | Decrease in TB incidence (cases per 100,000 people) from 2015 to 2025 | 1 | 327 |
|  |  |  | 2 | 613 |
|  |  |  | 3 | 175 |
| Mills, H. (2013) | (1) 6 INH; (2) No TPT | Incidence of active DS-TB per 100,000 from time 0 to time 100 | 1 | 416 to 71.4 |
|  |  |  | 2 | 416 to 445 |
|  |  | Incidence of active DR-TB per 100,000 from time 0 to time 100 | 1 | 9.78 to 6.42 |
|  |  |  | 2 | 9.78 to 1.13 |
| Mills, H. (2011) | (1) 0% coverage of 9 INH and continuous ART; (2) 1% coverage of 9 INH and continuous ART; (3) 10% coverage of 9 INH and continuous ART; (4) 25% coverage of 9 INH and continuous ART; (5) 50% coverage of 9 INH and continuous ART | Number of active TB cases per 100,000 from year 0 to year 50 (IPT introduced after 25 years) | 1 | 250 to 354 |
|  |  |  | 2 | 250 to 322 |
|  |  |  | 3 | 250 to 174 |
|  |  |  | 4 | 250 to 108 |
|  |  |  | 5 | 250 to 81 |
| Rhines, A. | (1) 90% INH (duration not specified) coverage; (2) 50% INH coverage; (3) Regular (5%) INH coverage | TB incidence per 100,000 in 2012 vs. 2032 | 1 | 1113 vs. 486 |
|  |  |  | 2 | 1113 vs. 539 |
|  |  |  | 3 | 1113 vs. 815 |
|  |  | TB mortality per 100,000 in 2012 vs. 2032 | 1 | 268 vs. 103 |
|  |  |  | 2 | 268 vs. 112 |
|  |  |  | 3 | 268 vs. 158 |
| Rose, D. (1992) | (1) INH (duration not specified); (2) No TPT | Active TB Cases per 10,000 individuals (with initial CD4 count of 350 cells/mm3) over 10 years | 1 | 2828 |
|  |  |  | 2 | 4912 |
|  |  | TB deaths per 10,000 individuals (with initial CD4 count of 350 cells/mm3) over 10 years | 1 | 313 |
|  |  |  | 2 | 541 |
| Rose, D. N. (2000) | (1) Screening and 6 INH for PLHIV with unknown tuberculin status; (2) 6 INH for PLHIV with positive TST | Range of number needed to screen to prevent 1 TB case over 10 years | 1 | 40 - 947 |
|  |  |  | 2 | 2 - 25 |
|  |  | Range of TB cases per 1000 persons over 10 years | 1 | 1.2 - 34.6 |
|  |  |  | 2 | 59 - 804 |
| Sumner, T. | (1) 12 INH; (2) No TPT | TB incidence per 1000 | 1 | 1.34 to 2.16 |
|  |  |  | 2 | 4.48 to 2.75 |
| Sterling, T. R. | (1) TB vaccine; (2) TST followed by 12 INH | Total TB cases per 10,000 | 1 | 650 |
|  |  |  | 2 | 192 |
|  |  | Total TB deaths per 10,000 | 1 | 64 |
|  |  |  | 2 | 19 |
| Vynnycky, E. | (1) 9 INH; (2) No TPT | TB incidence rate per 100,000 from 2007 to 2017 | 1 | 5365 to 2904 |
|  |  |  | 2 | 5365 to 4077 |
| Yan, I. | (1) Increase in ART coverage from 0-30%; (2) Increase in INH coverage from 0-30%; (3) Increase in CPT coverage from 0-30%. (Panel regression coefficients | Panel regression coefficient with WHO-estimated TB deaths; e.g. -0.009 means a 1% increase in intervention coverage = 0.90% decrease in TB deaths | 1 | -0.0097 |
|  |  |  | 2 | -0.0005 |
|  |  |  | 3 | -0.0337 |

TB = tuberculosis, HIV = human immunodeficiency virus, TPT = tuberculosis preventive therapy, LTBI = latent tuberculosis infection, PLHIV = people living with HIV, INH = isoniazid, TST = tuberculin skin test, IGRA = interferon gamma release assay, RPT = rifapentine, RIF = rifampin, AIDS = acquired immunodeficiency syndrome, DALY = disability adjusted life year, QALY = quality adjusted life year, CPT = cotrimoxazole preventive therapy, BCG = Bacillus Calmette–Guérin (vaccine), EMB = ethambutol, ART = antiretroviral therapy, CD4 = cluster of differentiation 4 (type of glycoprotein)

* In some cases, studies compared more strategies and had more modelled outcomes than are listed in this table

# REGRESSION ANALYSIS OF INPUT PARAMETERS ASSOCIATED WITH OUTCOMES

## Table K. Data used for regression analyses

| **Study Author** | **Publication Year** | **Set in LMIC** | **Considered ART Use** | **TPT Regimen** | **LTBI Prevalence** | **Per-Person Cost of TPT** | **Time Horizon** | **TPT Efficacy** | **TPT Adherence** | **Probability of Fatal Adverse Event** | **ICER TPT vs. no TPT** | **Percent Reduction in Active TB** | **Per-Person Cost of TPT Study Arm** |
| --- | --- | --- | --- | --- | --- | --- | --- | --- | --- | --- | --- | --- | --- |
| Awoke, T. D. | 2018 | yes | yes |  | 0.31 |  | 10 |  |  |  |  | 98% |  |
| Awoke, T. D. | 2018 | yes | yes |  | 0.31 |  | 10 |  |  |  |  | 99% |  |
| Azadi, M. | 2014 |  | yes | ST |  | $36 | 20 | 0.87 |  |  | $2,590 | 13% | $116 |
| Bacaer, N. | 2008 | yes | yes |  |  |  | 100 | 0.80 |  |  |  | 47% |  |
| Bachmann, M. O. | 2006 | yes | yes | ST |  | $39 | 10 | 0.67 |  |  |  |  |  |
| Bell, J. | 1999 | yes | no | ST |  | $37 | 8 | 0.67 |  | 0.054 | Cost-saving | 21% | $120 |
| Bell, J. | 1999 | yes | no | RI |  | $59 | 8 | 0.60 |  | 0.036 | $114 | 19% | $148 |
| Brewer, T. | 2001 | no | no |  | 0.05 |  | 10 | 0.90 |  | 0.0005 |  | 8% |  |
| Brewer, T. | 2001 | no | no |  | 0.05 |  | 10 | 0.90 |  | 0.0005 |  | 10% |  |
| Brewer, T. | 1996 | no | no |  | 0.08 |  | 10 | 0.60 |  | 0.1 |  | 14% |  |
| Brewer, T. | 1996 | no | no |  | 0.08 |  | 10 | 0.60 |  | 0.1 |  | 2% |  |
| Burgos, J. | 2009 | yes | yes | ST | 0.02 | $3 | 20 | 0.69 | 0.80 | 0.16 | $658 | 21% | $2,306 |
| Cohen, T. | 2016 |  | yes |  | 0.35 |  | 30 | 1.00 |  |  |  | 13% |  |
| Cohen, T. | 2016 |  | yes |  | 0.35 |  | 30 | 1.00 |  |  |  | 36% |  |
| Cohen, T. | 2016 |  | yes |  | 0.35 |  | 30 | 1.00 |  |  |  | 76% |  |
| Currie, C. | 2005 | yes | yes | ST |  |  | 20 |  |  |  | $412 | 19% | $83 |
| Currie, C. | 2005 | yes | yes | EX |  |  | 20 |  |  |  | $1,925 | 49% | $90 |
| de Siqueira Filha, N. | 2018 | yes | yes | ST | 0.16 | $29 | 1 |  |  |  |  |  | $847 |
| de Siqueira Filha, N. | 2018 | yes | no | ST | 0.02 | $4 | 1 |  |  |  |  |  | $182 |
| de Siqueira Filha, N. | 2018 | yes | no | ST | 0.02 | $4 | 1 |  |  |  |  |  |  |
| Dowdy, D. | 2014 | yes | yes | ST |  |  | 5 | 0.67 |  |  |  | 16% |  |
| Dye, C. | 2013 | yes | yes | EX |  |  | 40 | 0.85 |  |  |  | 73% |  |
| Dye, C. | 2013 | yes | yes | EX |  |  | 40 | 0.85 |  |  |  | 100% |  |
| Dye, C. | 2013 | yes | yes | EX |  |  | 40 | 0.85 |  |  |  | 100% |  |
| Dye, C. | 2013 |  | yes | EX |  |  | 40 | 0.85 |  |  |  | 98% |  |
| Ferguson, O. | 2020 | yes | yes | RI | 0.26 | $32 | 20 | 0.90 | 0.94 |  |  |  | $1,547 |
| Ferguson, O. | 2020 | yes | yes | RI | 0.26 | $28 | 20 | 0.90 | 0.74 |  |  |  | $1,540 |
| Freiman, J. | 2018 | yes | yes | ST |  |  | 43 | 0.90 |  | 0.05 |  | 19% |  |
| Freiman, J. | 2018 | yes | yes | ST |  |  | 38 | 0.90 |  | 0.05 |  | 12% |  |
| Freiman, J. | 2018 | yes | yes | ST |  |  | 36 | 0.90 |  | 0.05 |  | 20% |  |
| Freiman, J. | 2018 | yes | yes | EX |  |  | 43 | 0.90 |  | 0.05 |  | 25% |  |
| Freiman, J. | 2018 | yes | yes | EX |  |  | 38 | 0.90 |  | 0.05 |  | 17% |  |
| Freiman, J. | 2018 | yes | yes | EX |  |  | 36 | 0.90 |  | 0.05 |  | 26% |  |
| Gilbert, J. A. | 2016 | yes | yes | EX | 0.45 | $4 | 10 | 1.00 | 0.87 |  |  |  | $247 |
| Gourevitch, M. | 1998 | no | yes | ST | 0.53 | $36 | 5 | 0.65 |  | 0.05 |  | 40% | $604 |
| Gourevitch, M. | 1998 | no | yes | ST | 0.53 | $36 | 5 | 0.65 |  | 0.05 | $16,249 | 60% | $363 |
| Gourevitch, M. | 1998 | no | yes | ST | 0.53 | $36 | 5 | 0.65 |  | 0.05 |  | 87% | $121 |
| Gupta, S. | 2014 | yes | yes | EX | 0.26 | $1 | 3 | 0.49 |  |  | $7,497 | 39% | $784 |
| Gupta, S. | 2014 | yes | yes | EX | 0.26 | $1 | 3 | 0.49 |  |  | $8,166 | 35% | $783 |
| Gupta, S. | 2014 | yes | yes | EX | 0.26 | $1 | 3 | 0.49 |  |  | $4,387 | 35% | $608 |
| Gupta, S. | 2014 | yes | yes | ST | 0.26 | $1 | 3 | 0.11 |  |  | $7,977 | 34% | $756 |
| Gupta, S. | 2014 | yes | yes | EX | 0.26 | $1 | 3 | 0.49 |  |  | $7,799 | 33% | $741 |
| Gupta, S. | 2014 | yes | yes | EX | 0.26 | $1 | 3 | 0.49 |  |  | $4,923 | 31% | $608 |
| Gupta, S. | 2014 | yes | yes | ST | 0.26 | $1 | 3 | 0.11 |  |  | $9,056 | 30% | $756 |
| Gupta, S. | 2014 | yes | yes | ST | 0.26 | $1 | 3 | 0.11 |  |  | $4,565 | 29% | $581 |
| Gupta, S. | 2014 | yes | yes | EX | 0.26 | $1 | 3 | 0.49 |  |  | $8,910 | 29% | $740 |
| Gupta, S. | 2014 | yes | yes | EX | 0.26 | $1 | 3 | 0.49 |  |  | $4,280 | 28% | $565 |
| Gupta, S. | 2014 | yes | yes | ST | 0.26 | $1 | 3 | 0.11 |  |  | $5,426 | 24% | $581 |
| Gupta, S. | 2014 | yes | yes | EX | 0.26 | $1 | 3 | 0.49 |  |  | $5,144 | 23% | $565 |
| Gupta, S. | 2014 | yes | yes | ST | 0.26 | $1 | 3 | 0.11 |  |  | $10,376 | 22% | $708 |
| Gupta, S. | 2014 | yes | yes | ST | 0.26 | $1 | 3 | 0.11 |  |  | $14,040 | 17% | $710 |
| Gupta, S. | 2014 | yes | yes | ST | 0.26 | $1 | 3 | 0.11 |  |  | $6,086 | 16% | $535 |
| Gupta, S. | 2014 | yes | yes | ST | 0.26 | $1 | 3 | 0.11 |  |  | $10,475 | 9% | $537 |
| Guwatudde, D. | 2004 | yes | no |  | 0.80 |  | 20 | 0.40 |  |  |  | 6% |  |
| Guwatudde, D. | 2004 | yes | no |  | 0.80 |  | 20 | 0.40 |  |  |  | 10% |  |
| Guwatudde, D. | 2004 | yes | no |  | 0.80 |  | 20 | 0.40 |  |  |  | 16% |  |
| Guwatudde, D. | 2004 | yes | no |  | 0.80 |  | 20 | 0.40 |  |  |  | 21% |  |
| Hausler, H. P. | 2006 | yes | no | ST |  | $0 | 1 | 0.60 |  |  | $1,379 |  | $16 |
| Hausler, H. P. | 2006 | yes | no | ST |  | $0 | 1 | 0.60 |  |  | $696 |  | $117 |
| Heymann, S. J. | 1993 | yes | no |  |  | $67 | 10 | 0.43 |  |  |  | 50% |  |
| Heymann, S. J. | 1993 | yes | no |  |  | $67 | 10 | 0.43 |  |  |  | 100% |  |
| Houben, R. | 2014 | yes | no | ST | 1.00 |  | 3 |  |  |  |  | 0% |  |
| Houben, R. | 2014 | yes | no | ST | 1.00 |  | 3 |  |  |  |  | 0% |  |
| Houben, R. | 2014 | yes | no | RI | 1.00 |  | 3 |  |  |  |  | 19% |  |
| Houben, R. | 2014 | yes | no | RI | 1.00 |  | 3 |  |  |  |  | 28% |  |
| Hsieh, Y. | 2020 | yes | yes | ST |  | $4 | 12 | 0.67 | 0.84 | 0.00017 | $285 | 7% |  |
| Hsieh, Y. | 2020 | yes | yes | EX |  | $25 | 12 | 0.67 | 0.84 | 0.00017 | $1,086 | 10% |  |
| Jo, Y. | 2020 | no | no | RI |  | $465 | 30 | 0.93 | 0.78 | 0.00015 | $9,609 |  | $1,921 |
| Jo, Y. | 2020 | no | no | RI |  | $417 | 30 | 0.93 | 0.78 | 0.00015 | $4,564 |  | $3,050 |
| Jo, Y. | 2020 | no | no | RI |  | $457 | 30 | 0.93 | 0.78 | 0.00015 | $15,818 |  | $2,660 |
| Jo, Y. | 2020 | no | no | RI |  | $406 | 30 | 0.93 | 0.78 | 0.00015 | $7,090 |  | $3,627 |
| Johnson, K. | 2018 | yes | yes | RI | 0.26 | $6 | 20 | 0.58 | 0.47 | 0.034 |  |  | $4 |
| Johnson, K. | 2018 | yes | yes | ST | 0.26 | $76 | 20 | 0.63 | 0.74 | 0.034 |  |  | $63 |
| Jordan, T. J. | 1991 | no | no |  |  |  | 9 | 0.68 |  | 0.08 |  |  |  |
| Kapoor, S. | 2016 | yes | yes | ST | 0.21 | $25 | 12 | 0.63 | 0.82 | 0.025 | $1,051 | 57% | $25 |
| Kapoor, S. | 2016 | yes | yes | ST | 0.21 | $25 | 12 | 0.63 | 0.82 | 0.025 | $730 | 8% | $5 |
| Kendall, E. | 2019 | yes | yes | ST |  |  | 5 |  | 0.85 |  |  | 5% |  |
| Kendall, E. | 2019 | yes | yes | EX |  |  | 5 |  | 0.85 |  |  | 11% |  |
| Kim, H-Y. | 2018 | yes | yes | ST | 0.77 | $22 | 1 | 0.38 | 0.65 | 0.03 |  |  | $7 |
| Kim, H-Y. | 2018 | yes | yes | ST | 0.77 | $22 | 1 | 0.38 | 0.65 | 0.03 |  |  | $67 |
| Kowada, A. | 2013 | no | no | ST | 0.36 | $576 | 30 | 0.68 | 0.80 | 0.011 |  |  |  |
| Kunkel, A. | 2016 | yes | yes | EX |  |  | 34 | 0.72 | 0.07 |  |  | 24% |  |
| Kunkel, A. | 2016 | yes | yes | EX |  |  | 34 | 0.72 | 0.07 |  |  | 16% |  |
| Kunkel, A. | 2016 | yes | yes | ST |  |  | 34 | 0.72 | 0.07 |  |  | 6% |  |
| Linas, B. | 2011 | no | no | ST | 0.05 | $527 | 20 | 0.90 | 0.52 | 0.0055 |  |  |  |
| Long, E. | 2007 | yes | yes |  | 0.00 |  | 20 |  |  |  |  | 100% |  |
| Long, E. | 2007 | yes | yes |  | 0.00 |  | 20 |  |  |  |  | 52% |  |
| Maheswaran, H. | 2012 | yes | no | ST | 0.31 | $25 | 2 | 0.84 |  | 0.0023 |  |  | $263 |
| Maheswaran, H. | 2012 | yes | no | ST | 0.31 | $25 | 2 | 0.84 |  | 0.0023 |  |  | $387 |
| Maheswaran, H. | 2012 | yes | no | ST | 0.31 | $25 | 2 | 0.84 |  | 0.0023 |  |  | $228 |
| Maheswaran, H. | 2012 | yes | no | ST | 0.31 | $25 | 2 | 0.84 |  | 0.0023 |  |  | $263 |
| Maheswaran, H. | 2012 | yes | no | ST | 0.31 | $25 | 2 | 0.84 |  | 0.0023 |  |  | $201 |
| Maheswaran, H. | 2012 | yes | no | ST | 0.31 | $25 | 2 | 0.84 |  | 0.0023 |  |  | $287 |
| Maheswaran, H. | 2012 | yes | no | ST | 0.31 | $25 | 2 | 0.84 |  | 0.0023 |  |  | $247 |
| Maheswaran, H. | 2012 | yes | no | ST | 0.31 | $25 | 2 | 0.84 |  | 0.0023 |  |  | $268 |
| Maheswaran, H. | 2012 | yes | no | ST | 0.31 | $25 | 2 | 0.84 |  | 0.0023 |  |  | $299 |
| Mandal, S. | 2020 | both | yes | RI |  |  | 12 | 0.60 |  |  |  | 12% |  |
| Marx, F. M. | 2018 | yes | yes | EX | 0.60 |  | 10 |  | 0.66 |  |  | 71% |  |
| Masobe, P. | 1995 | yes | no | ST | 0.49 | $37 | 8 | 0.90 | 0.69 | 0.0048 | $513 | 65% | $144 |
| Mills, H. | 2013 | yes | no | ST |  |  | 100 |  |  |  |  | 84% |  |
| Mills, H. | 2011 | no | yes | ST | 0.40 |  | 10 |  | 0.50 |  |  | 9% |  |
| Mills, H. | 2011 | no | yes | ST | 0.40 |  | 10 |  | 0.50 |  |  | 51% |  |
| Mills, H. | 2011 | no | yes | ST | 0.40 |  | 10 |  | 0.50 |  |  | 69% |  |
| Mills, H. | 2011 | no | yes | ST | 0.40 |  | 10 |  | 0.50 |  |  | 77% |  |
| Perlman, D. C. | 2001 | no | no | ST | 0.20 | $1,256 | 5 | 0.65 |  |  | Cost-saving | 67% | $1,273 |
| Pho, M. | 2012 | yes | yes | EX |  | $108 | 10 | 0.78 | 1.00 | 0.0029 | $15,166 | 28% | $3,477 |
| Rhines, A. | 2018 | yes | yes |  | 0.50 |  | 20 | 1.00 | 0.90 |  |  | 40% |  |
| Rhines, A. | 2018 | yes | yes |  | 0.50 |  | 20 | 1.00 | 0.90 |  |  | 34% |  |
| Rose, D. | 1992 | no | no | ST |  |  | 10 | 0.51 |  | 0.04 |  | 42% |  |
| Rose, D. N. | 2000 | no | no | ST |  |  | 10 | 0.75 |  | 0.0002 |  |  |  |
| Samandari, T. | 2011 | yes | no | ST |  | $20 | 3 | 0.63 |  |  |  |  | $63 |
| Samandari, T. | 2011 | yes | no | ST |  | $20 | 3 | 0.63 |  |  |  |  | $73 |
| Sawert, H. | 1998 | no | no |  |  | $345 | 10 |  | 0.75 |  |  |  | $878 |
| Sawert, H. | 1998 | no | no |  |  | $345 | 10 |  | 0.75 |  |  |  | $967 |
| Sawert, H. | 1998 | no | no |  |  | $345 | 10 |  | 0.75 |  |  |  | $989 |
| Sawert, H. | 1998 | no | no |  |  | $345 | 10 |  | 0.75 |  |  |  | $975 |
| Sawert, H. | 1998 | no | no |  |  | $345 | 10 |  | 0.75 |  |  |  | $959 |
| Shayo, G. | 2018 | yes | yes | ST |  | $152 | 2 | 0.78 | 0.98 |  | $456 | 33% | $1,251 |
| Shrestha. R. | 2006 | yes | no | ST | 0.34 | $114 | 2 |  | 0.87 |  | $295 | 91% | $28 |
| Shrestha. R. | 2006 | yes | no | ST | 0.34 | $114 | 2 |  | 0.87 |  | $1,076 | 87% | $72 |
| Shrestha. R. | 2006 | yes | no | ST | 0.34 |  | 2 |  | 0.89 |  | $220 | 90% | $23 |
| Shrestha. R. | 2006 | yes | no | ST | 0.34 |  | 2 |  | 0.89 |  | $676 | 85% | $55 |
| Shrestha. R. | 2006 | yes | no | ST | 0.34 | $114 | 2 |  | 0.87 |  |  |  | $189 |
| Shrestha. R. | 2006 | yes | no | ST | 0.34 |  | 2 |  | 0.89 |  |  |  | $144 |
| Smith, T. | 2015 | yes | yes | ST |  | $27 | 3 | 0.21 |  |  |  | 86% | $700 |
| Smith, T. | 2015 | yes | yes | EX |  | $141 | 3 | 0.77 |  |  |  | 16% | $723 |
| Snyder, D. C. | 1999 | no | no | ST | 0.16 | $478 | 10 | 0.93 | 0.72 |  | Cost-saving | 52% | $458 |
| Sterling, T. R. | 1999 | no | no | ST | 0.65 |  |  | 0.98 |  | 0.0002 |  | 70% |  |
| Sumner, T. | 2016 | yes | yes | ST | 0.74 |  | 2 |  | 1.00 |  |  | 21% |  |
| Sutton, B. | 2009 | yes | no | ST | 0.64 | $45 | 3 | 0.83 |  |  | $1,144 |  | $158 |
| Tasillo, A. | 2017 | no | no | RI | 0.16 | $630 | 34 | 0.90 | 0.78 | 0.005 | $15,144 | 26% | $58 |
| Tasillo, A. | 2017 | no | no | RI | 0.16 | $630 | 34 | 0.90 | 0.78 | 0.005 | $19,340 | 35% | $39 |
| Tasillo, A. | 2017 | no | no | RI | 0.16 | $630 | 34 | 0.90 | 0.78 | 0.005 | $21,647 | 48% | $55 |
| Tasillo, A. | 2017 | no | no | RI | 0.16 | $630 | 34 | 0.90 | 0.78 | 0.005 | $25,461 | 55% | $50 |
| Terris-Prestholt, F. | 2008 | yes | yes | ST |  | $36 | 2 |  | 0.47 |  |  |  | $36 |
| Terris-Prestholt, F. | 2008 | yes | yes | ST |  | $36 | 2 |  | 0.47 |  |  |  | $48 |
| Vynnycky, E. | 2014 | yes | yes | ST |  |  | 5 | 0.63 |  |  |  | 29% |  |
| Yan, I. | 2016 | no | yes |  |  |  | 15 |  |  |  |  |  |  |

*All costs are listed in 2020 USD
TB = tuberculosis, LTBI = latent tuberculosis infection, TPT = tuberculosis preventive therapy, ART = antiretroviral therapy, ST = standard isonazid-based regimen (6, 9 and 12 months), EX = extended isoniazid-based regimen (>12 months), RI = rifamycin-based regimen, ICER = incremental cost per active TB case averted, USD = United States Dollars*


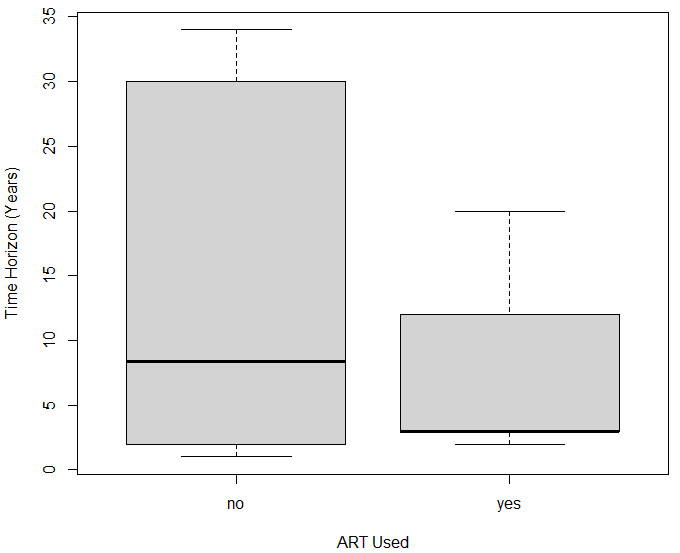


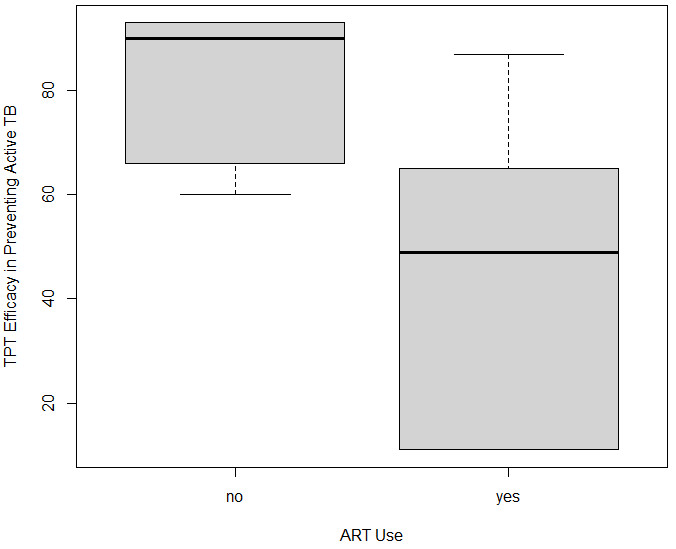


Fig A. Comparing the association between ART use, TPT efficacy and time horizon (model inputs). When a study considers ART use (i.e. considers a parameter for ART efficacy or cost), generally, the parameter for TPT efficacy is lower and the time horizon is shorter.


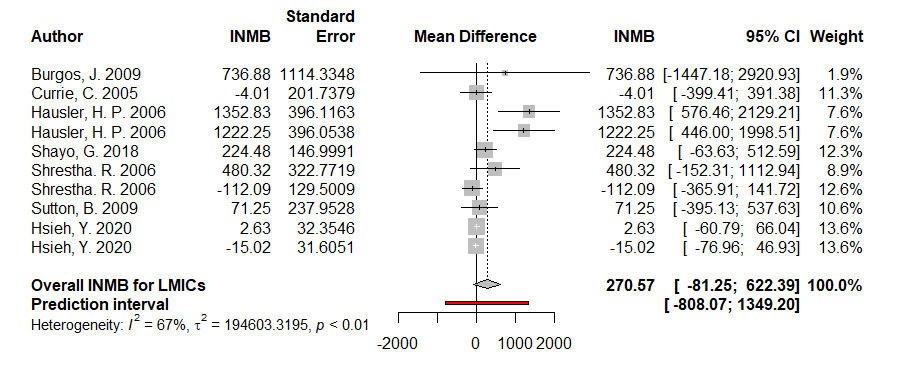


Fig B. Forest plot: pooling incremental net monetary benefit in low- and middle-income countries. Details on meta-analytical method: inverse variance method; Sidik-Jonkman estimator for $\tau$; Q-profile method for confidence interval of $\tau$ where applicable; Hartung-Knapp adjustment for random effects model. Abbreviations: INMB = incremental net monetary benefit, LMICs = low- and middle-income countries, CI = confidence interval


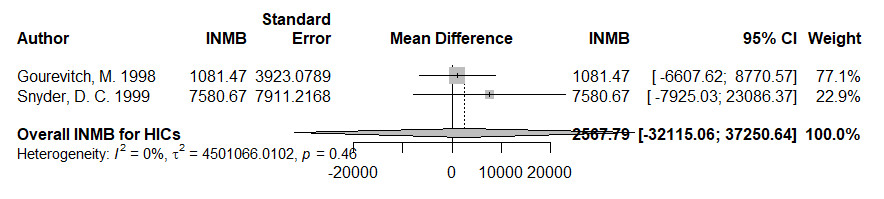


Fig C. Forest plot: pooling incremental net monetary benefit in high-income countries. Details on meta-analytical method: inverse variance method; Sidik-Jonkman estimator for $\tau$; Q-profile method for confidence interval of $\tau$ where applicable; Hartung-Knapp adjustment for random effects model. Abbreviations: INMB = incremental net monetary benefit, HICs = high-income countries, CI = confidence interval

# SUPPLEMENTAL RESULTS: SENSITIVITY AND THRESHOLD ANALYSES

The next two tables compare one-way sensitivity analysis results across studies. To note, several studies investigated the sensitivity of model outcomes to changes in ART coverage and efficacy. An analysis is provided below:

Outcomes may be sensitive to changes in ART coverage and efficacy: Twelve studies included ART coverage or impact as a variable in sensitivity analysis [5,9,14,17,18,19,20,27,28,33,40,56].  Of these, four studies found that increasing ART coverage increases related costs but providing TPT alongside ART remains cost-effective overall [9,18,27,40]. Five studies reported a further reduction in TB incidence as ART coverage expanded or its efficacy increased [5,14,19,28,56]. Two of these studies suggested that the respective reduction would be substantial [5,56] - one of them was set in Botswana [6] and one in South Africa [56]. Similarly, one study pointed out additive effects of ART and TPT in reducing TB incidence [17]. Lastly, one study found that TB incidence increased slightly as ART coverage increased; this was due to HIV prevalence increasing as individuals lived longer on ART [33].

## Table L. Comparing one-way sensitivity analysis results across cost and cost-effectiveness studies

| **Study** | **Publication Year** | **Name of key outcome driver (parameter) identified by sensitivity analysis** | **LOW parameter value** | **HIGH parameter value** | **Outcome** | **Outcome value that results from LOW parameter value** | **Outcome value that results from HIGH parameter value** |
| --- | --- | --- | --- | --- | --- | --- | --- |
| Azadi, M. | 2014 | Clinical training cost | $6,655 | NR | Incremental cost per DALY averted comparing TPT to no TPT | $3,075 | $2,055 |
|  |  | Disability weight for TB-HIV | 10 points worse than HIV | NR |  | $2,352 | $2,873 |
|  |  | THRio's hazard ratio | 0.7 | 0.97 |  | $2,486 | $2,650 |
| Bachmann, M. O. | 2006 | Monthly cost of ART drugs (exact range of values not reported) | Halved | Doubled | Incremental cost per QALY gained comparing TPT to no TPT | $1,562 | $3,159 |
|  |  | Monthly cost of laboratory tests (exact range of values not reported) | Halved | Doubled |  | $2,332 | $4,647 |
|  |  | Relative risk of TB infection on ART (independent of effects on CD4 and death) | 0.10 | 0.38 |  | $2,315 | $2,579 |
| Bell, J. | 1999 | Cost of preventive therapy | Half the cost | Double the cost | Incremental cost per QALY gained comparing TPT to no TPT | $13-$95 | $315-$636 |
|  |  | Number of secondary cases per active case | 2 | 10 |  | TPT less cost-effective | NR |
| Burgos, J. | 2009 | Cost for treating active TB | $500 | $15,000 | Incremental cost per QALY gained comparing TPT to no TPT | $1,050 | Cost-saving |
|  |  | ART coverage | 0% | 100% |  | $108 | $780 |
|  |  | Annual HIV incidence per 100 person-years | 0.3 | 4 |  | $708 | Cost-saving |
|  |  | TB incidence | 0.065 per 100 person years | 2 per 100 person years |  | $178 | $80 |
| de Siqueira Filha, N. (health system) | 2018 | Cost of medical appointment | -50% | +50% | Cost of prediagnosis, LTBI/HIV outpatient care | $112.05 | $222.60 |
|  |  |  |  |  | Cost of treatment, LTBI/HIV outpatient care | $383.45 | $876 |
|  |  | Cost of tests | -50% | +50% | Cost of prediagnosis, LTBI/HIV outpatient care | $116.15 | $214.40 |
|  |  |  |  |  | Cost of treatment, LTBI/HIV outpatient care | $514.65 | $613.20 |
|  |  | Cost of drugs | -50% | +50% | Cost of prediagnosis, LTBI/HIV outpatient care | $147.75 | $151.20 |
|  |  |  |  |  | Cost of treatment, LTBI/HIV outpatient care | $543.55 | $555.40 |
|  |  | Cost of ART | -50% | +50% | Cost of prediagnosis, LTBI/HIV outpatient care | $145.20 | $161.40 |
|  |  |  |  |  | Cost of treatment, LTBI/HIV outpatient care | $477.05 | $688.40 |
|  |  | Cost of LTBI drugs | -50% | +50% | Cost of treatment, LTBI/HIV outpatient care | $545.05 | $552.40 |
| Ferguson, O. | 2020 | 1 INH RPT Efficacy | 0.77 | 1 | Incremental cost-effectiveness of 1 INH RPT (relative to 3 INH RPT) | $5,220.76 | $665.25 |
|  |  | Proportion completing 1 INH RPT | 0.47 | 0.89 |  | $3,427.48 | $1,012.34 |
|  |  | Price of Rifapentine | $0.19 | $0.27 |  | $882.18 | $1,561.89 |
|  |  | LTBI Prevalence | 0.11 | 0.40 |  | $1,041.26 | $1,518.51 |
| Gilbert, J. A. | 2016 | ART coverage | 20% | 90% | Incremental cost per active TB case averted for annual TB/HIV screening and linkage to care (36 INH) compared to no TPT | $1,209.66 | $5,834.82 |
|  |  | Base TB/HIV screening cost | $10 | $120 |  | $942.82 | $4,073.70 |
|  |  | Proportion of those screened for TB/HIV initiating ART & INH | 80% | 20% |  | $1,725.54 | $2,730.62 |
| Gourevitch, M. | 1998 | LTBI prevalence | 10% among PLHIV | NR | TB cases prevented | 11 | NR |
|  |  |  |  |  | TB treatment costs prevented | $398,295 | NR |
|  |  | Including out-patient costs | NR | $3,009.90 | TB cases prevented | NR | 11 |
|  |  |  |  |  | TB treatment costs prevented | NR | $431,395 |
|  |  | Including multi-drug resistance costs | NR | $1,300,000 | TB cases prevented | NR | 11 |
|  |  |  |  |  | TB treatment costs prevented | NR | $498,370 |
| Gupta, S. | 2014 | ART coverage | 80% | 100% | Incremental cost per active TB case averted compared to no TPT | -$3,636,500 | -$3,340,700 |
|  |  | Specificity of cough (in ruling out active TB) | 0.65 | 0.92 |  | -$3,579,600 | -$3,331,200 |
|  |  | Sensitivity of cough (in ruling out active TB) | 0.19 | 0.62 |  | -$3,478,300 | -$2,428,600 |
| Hausler, H. P. | 2006 | Chest x-ray for INH screening | 0 (not having it) | 1 (including it) | Cost of TB case detected in primary health centre | $49 | $81 |
|  |  |  |  |  | Cost of TB case detected in community health clinic | $100 | $166 |
|  |  |  |  |  | Cost per person completing INH in primary health centre | $70 | $110 |
|  |  |  |  |  | Cost per person completing INH in community health clinic | $33 | $51 |
| Hsieh, Y. | 2020 | Episodes of INH-induced hepatotoxicity per  1000 INH initiations (3^rd^ year of anlaytic horizon on 6 INH) | 1 | 10 | Mean number of patients with  severe INH-associated hepatotoxicity | 1390 | 8632 |
|  |  | Episodes of INH-induced hepatotoxicity per  1000 INH initiations (3^rd^ year of analytic horizon on continuous INH) | 1 | 10 |  | 1137 | 6252 |
|  |  | Episodes of INH-induced hepatotoxicity per  1000 INH initiations (12^th^ year of analytic horizon on 6 INH) | 1 | 10 |  | 1664 | 14111 |
|  |  | Episodes of INH-induced hepatotoxicity per  1000 INH initiations (12^th^ year of analytic horizon on continuous INH) | 1 | 10 |  | 1327 | 8232 |
| Jo, Y. | 2020 | Expected decline in TB incidence (without targeted testing and treatment) | 0.00027 | 0.00079 | Incremental cost-effectiveness of targeted testing and treatment (TTT) in California | Cost saving | Dominated |
|  |  | IGRA cost | $60.81 | $120.59 |  | $2,711.72 | $11,828.09 |
|  |  | Cost of TB illness | $19,197.45 | $28,796.17 |  | $9,862.58 | $3,937.20 |
|  |  | Number of individuals testing positive | 12,766 | 19,149 |  | $5,184.32 | $8,638.13 |
|  |  | 3 INH RPT cost | $372.08 | $557.60 |  | $5,391.49 | $8,409.32 |
|  |  | Expected decline in TB incidence (without targeted testing and treatment) | 0.00027 | 0.00079 | Incremental cost-effectiveness of targeted testing and treatment (TTT) in Florida | Cost saving | $12,365.07 |
|  |  | IGRA cost | $53.60 | $108.22 |  | $264.88 | $6,075.86 |
|  |  | Cost of TB illness | $11,564.23 | $17,346.35 |  | $4,767.93 | $1,724.33 |
|  |  | Number of individuals testing positive | 4,990 | 7,485 |  | $2,450.96 | $3,420.83 |
|  |  | 3HP cost | $332.91 | $499.88 |  | $2,508.68 | $3,361.05 |
| Johnson, K. | 2018 | Percentage completing 3 INH RPT | 47% | 89% | Incremental cost per DALY averted comparing 3 INH RPT to 9 INH | $62,182 | $6,986 |
|  |  | Prevalence of LTBI | 11% | 40% |  | $22,565 | $6,070 |
|  |  | Mortality during treatment for TB | 5% | 18% |  | $16,744 | $5,659 |
|  |  | Price of 3 INH RPT per course | $15.12 | $72.72 |  | $535 | NR |
|  |  | Efficacy of 3 INH RPT | 39% | 79% |  | $11,696 | $5,732 |
| Kapoor, S. | 2016 | Proportion progressing to active TB (among those with CD4 > 200 cells/μl) | 0.03125 | 0.3 | Cost per DALY averted comparing 6 INH for CD4 > 200 cells/μl to no INH | $1,425 | $64.29 |
|  |  |  |  |  | Cost per DALY averted comparing 6 INH for CD4 ≤ 200 cells/μl to no INH | $1,658 | $61.47 |
|  |  | Relative risk reduction of progression to active TB among those taking INH | 0.2 | 0.94 | Cost per DALY averted comparing 6 INH for CD4 > 200 cells/μl to no INH | $875 | $114.29 |
|  |  |  |  |  | Cost per DALY averted comparing 6 INH for CD4 ≤ 200 cells/μl to no INH | $958 | $109.96 |
|  |  | Cost of 6 INH | $5.69 | $90.00 | Cost per DALY averted comparing 6 INH for CD4 > 200 cells/μl to no INH | $704 | $46.43 |
|  |  |  |  |  | Cost per DALY averted comparing 6 INH for CD4 ≤ 200 cells/μl to no INH | $709 | $44.16 |
| Kim, H-Y. | 2018 | Prevalence of LTBI | 0.454 | 0.932 | Incremental cost per DALY averted comparing INH for all to TST-indicated INH | $5,248 | $379 |
|  |  | Relative risk of active TB among those taking INH | 0.24 | 0.68 |  | $476 | $2,476 |
|  |  | Probability of developing severe drug-induced liver injury | 0.00005 | 0.025 |  | $131 | $1,731 |
| Linas, B. | 2011 | Rate of TB reactivation after remote infection | 0.04 | 0.11 | Incremental cost per QALY gained using TST screening prior to TPT compared to providing TPT to all | $16,800 | $11,600 |
|  |  |  |  |  | Incremental cost per QALY gained using IGRA screening prior to TPT compared to providing TPT to all | $35,300 | $20,400 |
| Masobe, P. | 1995 | Adherence to TPT | 41% | 68.50% | Total cost of INH chemoprophylaxis (assuming annual risk of developing active TB is 6.2%) | R1,538,566 | R26,952,775 |
|  |  | Annual risk of developing active TB | 5.50% | 7.90% | Total cost of INH chemoprophylaxis (assuming TPT adherence is 68.5%) | R22,624,241 | R40,570,398 |
| Perlman, D. C. | 2001 | INH effectiveness in preventing active TB | 40% | 90% | Total active TB cases during 5 years of follow-up | 18 | 6 |
|  |  |  |  |  | Total associated hospital costs during 5 years of follow-up | $655,084 | $218,361 |
|  |  | Chest x-ray adherence rate and cash incentive | 31% adherence with $0 incentive (baseline) | 50% adherence with $25 incentive | Total active TB cases prevented during 5 years of follow-up | 3 | 5 |
|  |  |  |  |  | Total associated hospital costs averted during 5 years of follow-up | $103,078 | $179,934 |
| Pho, M. | 2012 | Median CD4 count | 100 cells/mm3 | 500 cells/mm3 | Incremental cost per life-month gained | $1,019 | $3,013 |
| Samandari, T. | 2011 | Mortality among INH-resistant cohort | 0.1 | 0.25 | Incremental cost per death averted comparing symptom + chest x-ray + tracking policy to symptom only policy | $2,792,321 | $139,616 |
|  |  | Background mortality | 0.15 | 0.25 |  | $279,232 | $2,820,244 |
|  |  | Mortality among MDR cohort | 0.2 | 0.6 |  | $14,101,222 | $1,563,699 |
|  |  | Probability of abnormal chest x-ray | 0.05 | 0.2 | Incremental cost per TB case averted comparing symptom + chest x-ray + tracking policy to symptom only policy | $32,638 | $7,853 |
|  |  | Risk of INH-resistance | 0.2 | 0.5 |  | $21,431 | $2,454 |
|  |  | Probability of true abnormal chest x-ray with TB | 0.1 | 0.3 |  | $5,481 | $21,432 |
| Shayo, G. | 2018 | Medication cost for 6 INH | $84,267 | $102,993 | Incremental cost per TB case averted compared to no TPT | $383.64 | $428.22 |
|  |  |  |  |  | Incremental cost per death averted compared to no TPT | $164.58 | $183.71 |
|  |  | TB cases prevented by 6 INH | 378 | 462 | Incremental cost per TB case averted compared to no TPT | $451.03 | $369.03 |
|  |  |  |  |  | Incremental cost per death averted compared to no TPT | NA | NA |
|  |  | Deaths averted by 6 INH | 881 | 1077 | Incremental cost per TB case averted compared to no TPT | NA | NA |
|  |  |  |  |  | Incremental cost per death averted compared to no TPT | $193.52 | $158.30 |
| Shrestha, R. | 2006 | TST sensitivity | 0.2 | 1 | Incremental cost-effectiveness of TST strategy vs. no INH | NR | $200 |
|  |  |  |  |  | Incremental cost-effectiveness of non-TST strategy vs. no INH | $381 | $1,022 |
|  |  | LTBI prevalence | 0.17 | 0.51 | Incremental cost-effectiveness of TST strategy vs. no INH | $320 | $175 |
|  |  |  |  |  | Incremental cost-effectiveness of non-TST strategy vs. no INH | $1,764 | $432 |
|  |  | Treatment cost (9 INH and B6) | $57.07 | $139.30 | Incremental cost-effectiveness of TST strategy vs. no INH | $183 | $314 |
|  |  |  |  |  | Incremental cost-effectiveness of non-TST strategy vs. no INH | $585 | $1,424 |
| Shrestha, R. | 2007 | LTBI prevalence | 0.17 | 0.51 | Incremental cost-utility for targeted testing vs. no INH | $107 | $100 |
|  |  |  |  |  | Incremental cost-utility for test all vs. no INH | $107 | $104 |
|  |  | TST sensitivity | 0.2 | 0.99 | Incremental cost-utility for targeted testing vs. no INH | $115 | $101 |
|  |  |  |  |  | Incremental cost-utility for test all vs. no INH | $105 | $106 |
|  |  | Background risk of developing active TB | 0.034 | 0.1 | Incremental cost-utility for targeted testing vs. no INH | $110 | $69 |
|  |  |  |  |  | Incremental cost-utility for test all vs. no INH | $109 | $67 |
| Smith, T. | 2015 | Per-patient cost of ART | NA | | 55% influence on TB incidence, 46% influence on all-cause mortality | NA | |
|  |  | Percent of population below threshold CD4 250 cell/ml |  |  | 30% influence on TB incidence, 26% influence on all-cause mortality |  |  |
|  |  | Reduction in incident TB due to provision of ART |  |  | 50% influence on TB incidence, 19% influence on all-cause mortality |  |  |
| Snyder, D. C. | 1999 | Adherence to TPT | 30% | 95% | Cases of TB prevented | 16 | 32.5 |
|  |  |  |  |  | Average cost (savings in negative) per case prevented, compared to a scenario without screening for LTBI + TPT | $12,677 | -$6,674 |
|  |  | Proportion of cohort receiving medial evaluation | 75% | 100% (base case) | Cases of TB prevented | 21.9 | $1,776 |
|  |  |  |  |  | Average cost (savings in negative) per case prevented, compared to a scenario without screening for LTBI + TPT | 28.1 | -$3,724 |
|  |  | Proportion of cohort initiating preventive therapy | 75% | 100% (base case) | Cases of TB prevented | 23.2 | $822 |
|  |  |  |  |  | Average cost (savings in negative) per case prevented, compared to a scenario without screening for LTBI + TPT | 28.1 | -$3,724 |
| Sutton, B. | 2009 | Sputum smear cost | $6 | $18 | Cost per TB case prevented through 9 INH compared to no TPT | $732.03 | $1,178.63 |
|  |  | Culture cost | $2.32 | $6.96 |  | $868.99 | $1,041.67 |
|  |  | TB cases prevented by 9 INH | 13 | 21 |  | $1,273.78 | $764.27 |
| Tasillo, A. | 2017 | Probability of return for follow-up test | 0 | 1 | Incremental cost per QALY gained among PLHIV, comparing confirm negative strategy (IGRA+ means person has LTBI, IGRA- get TST, and if then TST-, classified as not having LTBI) to no testing before providing 3 INH RPT | $175,648 | $63,731 |
|  |  | Prevalence of LTBI | 0 | 100% |  | $205,181 | $15,544 |
| Terris-Prestholt, F. | 2008 | Allocation of ZAMBART administrative costs | Allocated equally across sites | Varies by site | Cost per person completing INH in Chawama | $78.32 | $27.48 |
|  |  |  |  |  | Cost per person completing INH in Matero | $105.46 | $35.39 |
|  |  |  |  |  | Cost per person reached for ProTEST cooridnation in Chawama | $1.47 | $1.10 |
|  |  |  |  |  | Cost per person reached for ProTEST cooridnation in Matero | $14.47 | $5.26 |
|  |  |  |  |  | Cost per ProTEST clinic visit in Chawama | $6.57 | $9.38 |
|  |  |  |  |  | Cost per ProTEST clinic visit in Matero | $19.15 | $25.53 |

*All currencies listed are in the currency of their study; they have not been standardized to 2020 USD*

*NR = Not Reported, ART = Antiretroviral Therapy, HIV = Human Immunodeficiency Virus, PLHIV = People Living with HIV, INH = Isoniazid, RPT = Rifapentine, QALY = Quality adjusted life years, DALY = Disability adjusted life years, TB = Tuberculosis, TPT = Tuberculosis Preventative Therapy, TST = Tuberculin Skin Test, IGRA = Interferon Gamma Release Assay, CXR = chest x-ray, HAART = Highly Active Antiretroviral Therapy, MDR = Multi-Drug Resistant, CD4 = cluster of differentiation 4 (type of glycoprotein), GDP = Gross Domestic Product, WHO = World Health Organization*

## Table M. Comparing one-way sensitivity analysis results across studies that only report effectiveness or utility outcomes

| **Study** | **Publication Year** | **Name of key outcome driver (parameter) identified by sensitivity analysis** | **LOW parameter value** | **HIGH parameter value** | **Outcome** | **Outcome value that results from LOW parameter value** | **Outcome value that results from HIGH parameter value** |
| --- | --- | --- | --- | --- | --- | --- | --- |
| Basu, S. | 2009 | Per capita transmission rate for INH-resistant strain (incorporating fitness) | 0.5 | 1.2 | Change in TB incidence per 100,000 overall population members compared to no TPT | -10.65 | 60.94 |
|  |  | Proportion of infected people who develop primary progressive TB | 0.32 | 0.74 |  | 13.65 | -15.89 |
|  |  | Proportion of eligible patients adhering to INH and not discontinuing due to adverse effects | 0.21 | 0.56 |  | 12.52 | -7.85 |
| Dowdy, D. | 2014 | Protective efficacy of INH against TB reactivation | 0.5 | 1 | Projected reduction in HIV associated TB incidence after 5 years among those on 6 INH | 25.60% | 11.10% |
|  |  | Relapse rate after TB treatment | 0.006/yr | 0.3/yr |  | 10.90% | 19.30% |
|  |  | Proportion of TB infections progressing rapidly among PLHIV | 0.09 | 1 |  | 15.60% | 23.60% |
| Freiman, J. | 2018 | Brazil: Fatal INH toxicity | NR | NR | Incremental gain in life expectancy (months) comparing ART+INH to ART alone | 5.43 months | -15.62 months |
|  |  | India: TB mortality | NR | NR |  | 51.73 months | -2.59 months |
|  |  | Uganda: TB mortality | NR | NR |  | 22.50 months | -2.48 months |
| Guwatudde, D. | 2004 | HIV infection prevalence at baseline | 5% | 30% | Percentage reduction in prevalence of TB after 20 years from baseline, given 50% coverage of TPT | 10.2 | 11.1 |
|  |  | TB transmission coefficient | 4.88x10^-7 | 9.75x10^-7 |  | 13.9 | 7.2 |
|  |  | Proportion of smear negative TB among PLHIV | 0.5 | 0.7 |  | 10.3 | 10.3 |
|  |  | Efficacy of preventive therapy | 0.24 | 0.65 |  | 13.2 | 6 |
| Kendall, E. | 2019 | Persistence of reduced TB progression risk after INH | 0.27 | 0.74 | Correlation with incidence reduction, mortality reduction is 0.9 | NA | NA |
|  |  | Reduction in risk of TB progression, INH vs no INH | 0.49 | 0.65 | Correlation with incidence reduction, mortality reduction is 0.9 |  |  |
|  |  | HIV progression rate, from [CD4+ > 500] to [200 < CD4+ < 500] | 0.11 | 0.14 | Correlation with incidence reduction, mortality reduction is 0.3 |  |  |
| Kunkel, A. | 2016 | TB transmission coefficient | NR | x1.25, x1.5, x1.75 base case value | Overall TB | NR | Higher as transmission increases |
|  |  |  |  |  | Cumulative mortality | NR | Higher as transmission increases |
|  |  |  |  |  | INHR TB | NR | Higher as transmission increases |
| Long, E. | 2007 | HIV progression rate with no TB | 0.01 | 0.2 | HIV term in R_0_ | 6.59 | 1.09 |
|  |  | Number of sexual partners among PLHIV | 1 | 10 |  | 0.69 | 3.63 |
|  |  | Probability of transmission with partner | 0.01 | 0.06 |  | 0.73 | 2.84 |
|  |  | Active TB sufficient contact rate | 2 | 10 | TB term in R_0_ | 1.76 | 8.81 |
|  |  | TB progression rate with no HIV | 0.001 | 0.01 |  | 1.06 | 7.11 |
|  |  | Active TB death rate | 0.1 | 0.3 |  | 2.46 | 6.66 |
| Mandal | 2020 | Effective coverage of preventative therapy (status quo comparator) * | 0 | 1 | Percent reduction of incidence relative to 2015 | 0 | 8.56 (6.44 -13.19) |
|  |  | Effective coverage of preventative therapy (improved TB cascade comparator) ** | 0 | 1 |  | 0 | 6.65 (5.15-8.13) |
|  |  | Effective coverage of preventative therapy (status quo comparator)* | 0 | 1 | Percent reduction of mortality relative to 2015 | 0 | 8.16 (5.56 – 12.50) |
|  |  | Effective coverage of preventative therapy (improved TB cascade comparator) ** | 0 | 1 |  | 0 | 4.26 (2.75 – 6.45) |
| Marx, F. M. | 2018 | Probability of receiving secondary INH after completing treatment for active TB (given targeted active case finding every 12 months) | 0% | 90% | Total TB cases averted | 895 | 2010 |
|  |  |  |  |  | Total TB deaths averted | 314 | 208 |
|  |  | Frequency of targeted active case finding (given 50% probability of receiving secondary INH after completing treatment for active TB) | Every 6 months | Every 24 months | Total TB cases averted | 1552 | 2129 |
|  |  |  |  |  | Total TB deaths averted | 350 | 256 |
| Mills, H. | 2013 | Partial immunity in transition from latent singly infected to latent multiply infected, following reinfection, PLHIV | 0.6375 | 1 | Correlation coefficient with active DR-TB incidence over 20 years | 0 | 0.69 |
|  |  |  |  |  | Correlation coefficient with latent DR-TB prevalence over 20 years | 0 | 0.72 |
|  |  | Death at susceptible stage for those without HIV | 0.015 | 0.025 | Correlation coefficient with active DR-TB incidence over 20 years | 0 | 0.42 |
|  |  |  |  |  | Correlation coefficient with latent DR-TB prevalence over 20 years | 0 | 0.2 |
| Rhines, A. | 2018 | TB transmission rate | 0.0000001 | 0.0000015 | TB incidence after 20 years | 1221 per 100,000 | 1640 per 100,000 |
|  |  | Recovery rate | 0.1 | 0.9 | TB incidence after 20 years | 1036 per 100,000 | 799 per 100,000 |
|  |  | TB activation rate in PLHIV | 0.005 | 0.9 | TB incidence after 20 years | 726 per 100,000 | 808 per 100,000 |
|  |  | Rate of ceasing INH | 0.05 | 0.15 | TB incidence after 20 years | 1191 per 100,000 | 1679 per 100,000 |
| Rose, D. | 1992 | Cohort's initial CD4 count (cells/mm^3) | <200 | 650 | TB cases, without 12 INH | 3771 | 577 |
|  |  |  |  |  | TB deaths, without 12 INH | 447 | 565 |
|  |  |  |  |  | TB cases, with 12 INH | 2302 | 2909 |
|  |  |  |  |  | TB deaths, with 12 INH | $273 | $288 |
| Sumner, T. | 2016 | Prevalence of INH resistance | 0% | 24% | Annual risk of TB infection (%) | 4.0 (2.7-5.9) | 4.0 (2.6-5.9) |
|  |  |  |  |  | Proportion cured following 12 INH (%) | 23.2 (1.0-61.5) | 39.8 (1.9-86.7) |

*All currencies listed are in the currency of their study; they have not been standardized to 2020 USD*

*NR = Not Reported, ART = Antiretroviral Therapy, HIV = Human Immunodeficiency Virus, PLHIV = People Living with HIV, INH = Isoniazid, RPT = Rifapentine, TB = Tuberculosis, TPT = Tuberculosis Preventative Therapy, TST = Tuberculin Skin Test, IGRA = Interferon Gamma Release Assay, CXR = chest x-ray, HAART = Highly Active Antiretroviral Therapy, MDR = Multi-Drug Resistant, DR = Drug Resistant, CD4 = cluster of differentiation 4 (type of glycoprotein)*

******The status quo comparator is referring to current TB services continuing without change.*

*** The improved TB cascade is referring to ongoing efforts to improve TB control other than preventative therapy.*

## Table N. Threshold analysis results among included studies (that reported key thresholds where conclusions changed)

| **Study Author** | **Publication Year** | **New conclusion vs. original conclusion** | **Parameter that changed conclusions** | **Value that this parameter changed conclusions at** | **Base case value of this parameter** |
| --- | --- | --- | --- | --- | --- |
| **Cost/Cost-Effectiveness Studies** | | | | | |
| Bachmann, M. O.* | 2006 | **New:** providing TPT to those with CD4 counts $\leq$200 ul is no longer cost-saving compared to no TPT  **Old:** providing TPT to those with CD4 counts $\leq$200 ul is cost-saving compared to no TPT | Relative risk of developing active TB among those that are taking INH | 0.8 | 0.5 |
| Bell, J. | 1999 | **New:** The only regimen that results in medical care cost savings is 6 INH (compared to the cost of treating active TB)  **Old:** 3 INH RIF and 6 INH result in medical care cost savings (compared to the cost of treating active TB) | Per-person cost of treating active TB | $113.51 | $227.02 |
| Burgos, J. | 2009 | **New:** 6 INH becomes cost-saving compared to no preventive therapy  **Old:** 6 INH is more effective at averting active TB cases compared to no preventive therapy, but costs more | Annual risk of HIV infection | > 6% | 2% |
| Ferguson, O. | 2020 | **New:** 1 INH RPT is cost-effective compared to 3 INH RPT at a willingness to pay threshold of $1,500 per DALY averted  **Old:** 1 INH RPT is not cost-effective compared to 3 INH RPT at a willingness to pay threshold of $1,500 per DALY averted | Proportion completing 1 INH RPT: a) in a setting where the LTBI prevalence is 0.26 b) in a setting where the LTBI prevalence is 0.50 | a) 0.90  b) 0.81 | a) 0.74 b) 0.74 |
| Gilbert, J. A. | 2016 | **New:** Community-based TB/HIV screening with linkage to care (36 INH) is very cost-effective  **Old:** Community-based TB/HIV screening with linkage to care (36 INH) is cost-effective, but moderately so | Willingness to pay threshold (GDP per capita in South Africa) | $19,854 | $6,618 |
| Johnson, K. | 2018 | **New:** 3 INH RPT likely to be cost-effective relative to 9 INH at a willingness to pay of $1000 per DALY averted  **Old:** 3 INH RPT is not cost-effective compared to 9 INH at a willingness to pay of $1000 per DALY averted | Price of rifapentine and rate of treatment completion | $20 per course per person, 85% treatment completion | $72 per course per person, 74% treatment completion |
| Linas, B. | 2011 | **New:** no indication before providing 9 INH is cost-effective compared to indication using IGRA or TST  **Old:** indication using IGRA and TST before providing 9 INH is cost-effective compared to no indication | TST specificity, IGRA specificity | < 0.6 for TST,  < 0.675 for IGRA | 0.92 for TST, 0.99 for IGRA |
| Maheswaran, H. | 2012 | **New:** No screening strategy before providing 6 INH would be cost-effective  **Old:** Screening for active TB before providing 6 INH is cost-effective compared to providing it to all | Active TB prevalence | < 5% | 8.60% |
| Shrestha, R. | 2006 | **New:** indicating 9 INH using TST becomes more cost effective than providing TPT to all  **Old:** providing 9 INH to all is more cost effective than indicating it using TST | Adherence to TPT among those who are in the “no indication” cohort | 72% | 92% |
| Snyder, D. C. | 1999 | **New:** Screening for infection in methadone maintenance clinics and providing directly observed preventive therapy (12 INH) is no longer cost saving compared to no screening/no preventive treatment  **Old:** Screening for infection in methadone maintenance clinics and providing directly observed preventive therapy (12 INH) is cost saving compared to no screening/no preventive treatment | Proportion of people that begin preventive therapy (among those that are found to have infection through the screening process) | The threshold lies between 75% and 100% | 100% |
| Tasillo, A. | 2017 | **New:** Using TST prior to initiating 3 INH RPT is more cost-effective than using IGRA or no test (among those with no comorbidities)  **Old:** The most commonly preferred strategy to screen before initiating 2 INH RPT is confirm negative (patients with a negative IGRA given TST, with either positive resulting in LTBI diagnosis). Using TST prior to initiating TPT is most cost-effective in only 6% of simulations. | TST sensitivity | > 92.5% | 88.6% |
| **Effectiveness Only Studies** | | | | | |
| Jordan, T. J. | 1991 | **New:** Isoniazid preventative therapy is no longer favorable for black women  **Old:** Isoniazid preventative therapy is favourable for all PLHIV | INH-induced mortality for tuberculin-negative black women | 6-9.6% | 5.9% |
| Dowdy, D. | 2014 | **New:** 6 INH reduces TB incidence among PLHIV by 4.6%  **Old:** 6 INH reduces TB incidence among PLHIV by 15.6% | Duration of protection (from developing active TB) due to INH | 6 months | 5 years |
| Freiman, J. | 2018 | **New:** 6 INH improves life expectancy more than ART alone in Brazil  **Old:** Neither 6 INH nor 36 INH improve life expectancy more than ART alone in Brazil | INH toxicity | < 0.023 | 0.029 |
| Kendall, E. | 2019 | **New:** The epidemiological impact of INH is reduced by more than 80%, i.e. <1.04% reduction in TB incidence among those taking 12 INH and <2.1% reduction in TB incidence among those taking lifetime INH  **Old:** There is 5.2% reduction in TB incidence among those taking 12 INH and a 10.5% reduction in TB incidence among those taking lifetime INH | Effect of INH | Effect of INH restricted only to the prevention of infection during treatment | Effect of INH includes prevention of infection during treatment and progression to disease |
| Rose, D. N. | 2000 | **New:** The risks of 6 INH outweigh the benefits for PLHIV with positive TST  **Old:** The benefits of 6 INH outweigh the risks for all PLHIV | Probability of fatal toxicity with INH | 15.20% | 0.002% |
| Sterling, T. R. | 1999 | **New:** TB vaccination prevents more cases of active TB than TST indicated 12 INH (there were 6 other combinations of these three variables that gave this conclusion; only one combination is presented here)  **Old:** TST indicated 12 INH prevents more cases of active TB than TB vaccination | Risk of TB despite taking INH (i.e. 1 - TPT efficacy) | 4.2% | 0.017% |
|  |  |  | Probability that someone has TB if they are anergic | 11.5% | 0.031% |
|  |  |  | Protective efficacy of vaccine | 74% | 50% |

*All currencies listed are in the currency of their study; they have not been standardized to 2020 USD*

*ART = Antiretroviral Therapy, HIV = Human Immunodeficiency Virus, PLHIV = People Living with HIV, INH = Isoniazid, RPT = Rifapentine, QALY = Quality adjusted life years, DALY = Disability adjusted life years, TB = Tuberculosis, TPT = Tuberculosis Preventative Therapy, TST = Tuberculin Skin Test, IGRA = Interferon Gamma Release Assay, CXR = chest x-ray, HAART = Highly Active Antiretroviral Therapy, MDR = Multi-Drug Resistant, CD4 = cluster of differentiation 4 (type of glycoprotein)*

** Example interpretation: When the relative risk of developing active TB among those that are taking INH is equal to 0.5, providing TPT to those with CD4 counts* $\leq$*200 ul is cost-saving compared to no TPT. When this relative risk increases to 0.8, then providing TPT to those with CD4 counts* $\leq$*200 ul is no longer cost-saving compared to no TPT.*

## Table O. Effect of 0.5x and 3x GDP per capita willingness-to-pay threshold on univariable analysis of incremental net monetary benefit

| ***Categorical variables: group of interest*** | ***Categorical variables: reference*** | ***Number of strategies*** | ***Incremental Net Monetary Benefit***  ***(Willingness-to-pay = 0.5x GDP per capita)*** | | ***Incremental Net Monetary Benefit***  ***(Willingness-to-pay = 3x GDP per capita)*** | |
| --- | --- | --- | --- | --- | --- | --- |
|  |  |  | ***Estimate*** | ***95% CI*** | ***Estimate*** | ***95% CI*** |
| Strategy is set in a high-income country | Strategy is set in a low- or middle-income country | 46 | $1346 | $134 to $4164 | $11527 | $414 to $21053 |
| Strategy includes ART-related variables in analysis | Strategy does not include ART-related variables in analysis | 47 | -$254 | -$608 to -$119 | -$1603 | -$4505 to -$357 |
| Strategy models 3 INH RPT | Strategy models an isoniazid regimen 6-12 months^*^ | 47 | $68 | $31 to $1327 | $152 | -$106 to $11649 |
| Strategy models an isoniazid regimen > 12 months^†^ | Strategy models an isoniazid regimen 6-12 months^*^ | 47 | -$76 | -$238 to -$60 | -$106 | -$602 to -$12 |
| ***Continuous variables*** | ***Definition of unit increase*** | ***Number of strategies*** | ***Incremental Net Monetary Benefit***  ***(Willingness-to-pay = 0.5x GDP per capita)*** | | ***Incremental Net Monetary Benefit***  ***(Willingness-to-pay = 3x GDP per capita)*** | |
|  |  |  | ***Estimate*** | ***95% CI*** | ***Estimate*** | ***95% CI*** |
| LTBI prevalence | 10% | 32 | -$75 | -$152 to $158 | $10 | $2 to $115 |
| Time Horizon | 1 year | 47 | $7 | $3 to $41 | $6 | -$6 to $156 |
| TPT efficacy in preventing active disease | 10% | 41 | $41 | $15 to $52 | $75 | $18 to $137 |
| Level of TPT adherence | 10% | 21 | -$345 | -$1530 to -$14 | -$1202 | -$10481 to $149 |
| Probability of fatal adverse event | 0.1% | 18 | $2 | -$52 to $7 | $12 | -$483 to $76 |

*Values in this table represent the median change incremental net monetary benefit between PLHIV given TPT and PLHIV not given TPT; values were estimated using quantile regression.** *These regimens include 6, 9, and 12 months of isoniazid*

*^†^ These regimens include 36 months and lifetime isoniazid*
*Abbreviations: CI = confidence interval, ART = antiretroviral therapy, PLHIV = people living with HIV, INH = isoniazid, LTBI = latent tuberculosis infection, TPT = tuberculosis preventative therapy, RPT = rifapentine, USD = United States Dollars*

## Table P. Effect of 0.5x and 3x GDP per capita willingness-to-pay threshold on multivariable analysis of incremental net monetary benefit

|  |  | **Model 1 (n = 46)** | | **Model 2 (n =46)** | | **Model 3 (n = 46)** | | **Model 4 (n = 46)** | |
| --- | --- | --- | --- | --- | --- | --- | --- | --- | --- |
| ***Categorical variables: group of interest*** | ***Categorical variables: reference*** | ***Incremental Net Monetary Benefit***  ***(Willingness-to-pay = 0.5x GDP per capita)*** | | | | | | | |
|  |  | ***Estimate*** | ***95% CI*** | ***Estimate*** | ***95% CI*** | ***Estimate*** | ***95% CI*** | ***Estimate*** | ***95% CI*** |
| Set in a high-income country | Set in a low- or middle-income country | $1267 | $373 to $20,915 | $1069 | $430 to $19,680 | $1186 | $355 to $20,305 | $1101 | $359 to $19,284 |
| Strategy includes ART-related variables in analysis | Strategy does not include ART-related variables in analysis | -$295 | -$600 to -$125 | -$285 | -$699 to -$179 | -$222 | -$559 to -$68 | -$223 | -$559 to -$190 |
| Strategy models rifamycin-based regimen | Strategy models an isoniazid regimen 6-12 months** | -$210 | -$9062 to $407 | -$230 | -$4681 to -$100 | -$193 | -$20,878 to $483 | -$184 | -$4121 to -$25 |
| Strategy models an isoniazid regimen > 12 months^†^ | Strategy models an isoniazid regimen 6-12 months** | -$13 | -$179 to $28 | -$118 | -$132 to $14 | -$47 | -$208 to  -$10 | -$63 | -$197 to -$15 |
| ***Categorical variables: group of interest*** | ***Categorical variables: reference*** | ***Incremental Net Monetary Benefit***  ***(Willingness-to-pay = 3x GDP per capita)*** | | | | | | | |
|  |  | ***Estimate*** | ***95% CI*** | ***Estimate*** | ***95% CI*** | ***Estimate*** | ***95% CI*** | ***Estimate*** | ***95% CI*** |
| Set in a high-income country | Set in a low- or middle-income country | $11,821 | $3201 to $131,035 | $12,600 | $3651 to $123,156 | $11,972 | $3123 to $127,070 | $13,104 | $3213 to $120,626 |
| Strategy includes ART-related variables in analysis | Strategy does not include ART-related variables in analysis | -$2061 | -$4387 to -$905 | -$1924 | -$4582 to -$1273 | -$2198 | -$4222 to -$845 | -$1460 | -$3792 to -$631 |
| Strategy models rifamycin-based regimen | Strategy models an isoniazid regimen 6-12 months** | -$2145 | -$55,068 to $2774 | -$1915 | -$19,940 to -$1239 | -$2159 | -$1.4x10^17^ to $6654 | -$1659 | -$101,149 to $1461 |
| Strategy models an isoniazid regimen > 12 months^†^ | Strategy models an isoniazid regimen 6-12 months** | $194 | -$38 to $377 | $109 | -$34 to $295 | $267 | -$360 to $419 | -$160 | -$533 to $309 |

*Values in this table represent the median change in incremental net monetary benefit between PLHIV given TPT and PLHIV not given TPT; values were estimated using quantile regression. Each column titled “Model” illustrates the results of one multivariable model. Model 1 only included only categorical variables, Model 2 included time horizon in addition, Model 3 included TPT efficacy, and Model 4 included both; missing values for time horizon and TPT efficacy were imputed using medians. Estimates for time horizon and TPT efficacy aren’t shown as they were negligible.*** *These regimens include 6, 9, and 12 months of isoniazid*

*^†^ These regimens include 36 months and lifetime isoniazid
Abbreviations: CI = confidence interval, ART = antiretroviral therapy, PLHIV = people living with HIV, INH = isoniazid, LTBI = latent tuberculosis infection, TPT = tuberculosis preventative therapy, RPT = rifapentine, USD = United States Dollar*

## Table Q. Effect of 0.5x and 3x GDP per capita willingness-to-pay threshold on pooling analysis of incremental net monetary benefit

|  | **Value** | | | $\boldsymbol{\tau}$ | | $\boldsymbol{I}^{\boldsymbol{2}}$ | |
| --- | --- | --- | --- | --- | --- | --- | --- |
|  | ***Estimate*** | ***95% CI*** | ***p value*** | ***Estimate*** | ***95% CI*** | ***Estimate*** | ***95% CI*** |
| **Low- and Middle-Income Countries** | | | | | | | |
| Pooled Incremental Net Monetary Benefit (n = 10)  ***(Willingness-to-pay = 0.5x GDP per capita)*** | $46 | -$121 to $212 | 0.55 | $208 | $81 to $429 | 71% | 44% to 85% |
| Pooled Incremental Net Monetary Benefit (n = 10)  ***(Willingness-to-pay = 3x GDP per capita)*** | $1198 | $40 to $2356 | 0.04 | $1455 | $667 to $2957 | 77% | 57% to 87% |
| **High-Income Countries** | | | | | | | |
| Pooled Incremental Net Monetary Benefit (n = 2)  ***(Willingness-to-pay = 0.5x GDP per capita)*** | $1281 | -$19,154 to $21,718 | 0.57 | $1357 | NA | 0% | NA |
| Pooled Incremental Net Monetary Benefit (n = 2)’ ***(Willingness-to-pay = 3x GDP per capita)*** | $7801 | -$84,485 to $100,088 | 0.48 | $5263 | NA | 0% | NA |

# SUPPLEMENTAL RESULTS: FIGURES


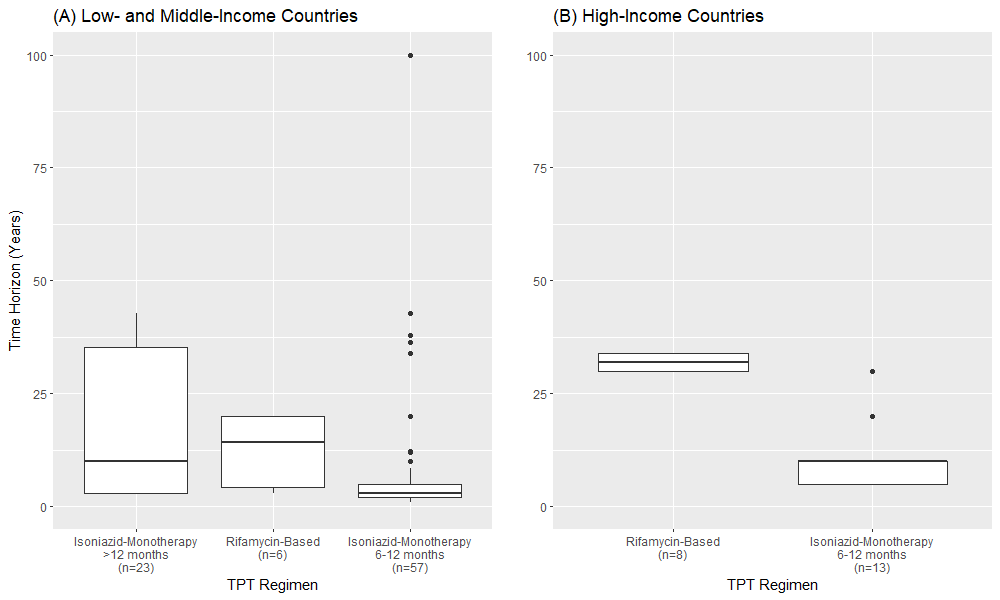


Fig D. Model inputs: comparing time horizon by TPT regimen category and country-level income. The thick horizontal line within each box indicates the median time horizon that pertains to that regimen. The dots in graph (A) represent study arms that were outliers, and had a time horizon that was longer or shorter than usual. Rifamycin-based regimens consist of three months of isoniazid and rifampin as well as 3 months of isoniazid and rifapentine.

Abbreviations: TPT = tuberculosis preventive therapy


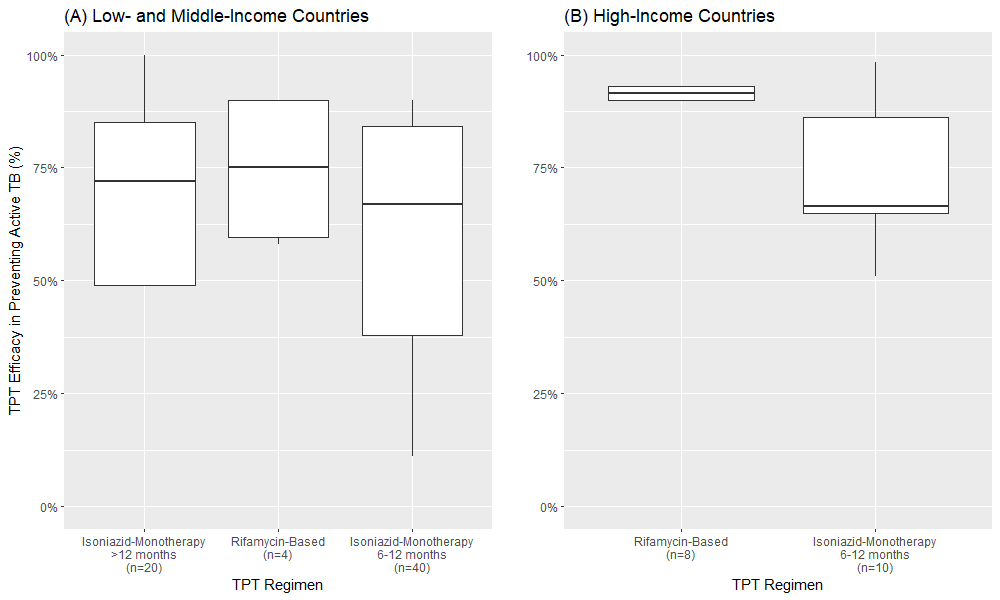


Fig E. Model inputs: comparing TPT efficacy in preventing active TB by TPT regimen category and country-level income. The thick horizontal line within each box indicates the median efficacy that pertains to that regimen. The dots in graph (A) represent study arms that were outliers, and had a value for TPT efficacy that was more or less than usual. Rifamycin-based regimens consist of three months of isoniazid and rifampin as well as 3 months of isoniazid and rifapentine.

Abbreviations: TPT = tuberculosis preventive therapy

Fig F. Model inputs: comparing level of TPT adherence by TPT regimen category and country-level income. The thick horizontal line within each box indicates the median level of adherence that pertains to that regimen. The dots in graph (A) represent study arms that were outliers, and had a value for TPT adherence that was more or less than usual. Rifamycin-based regimens consist of three months of isoniazid and rifampin as well as 3 months of isoniazid and rifapentine.


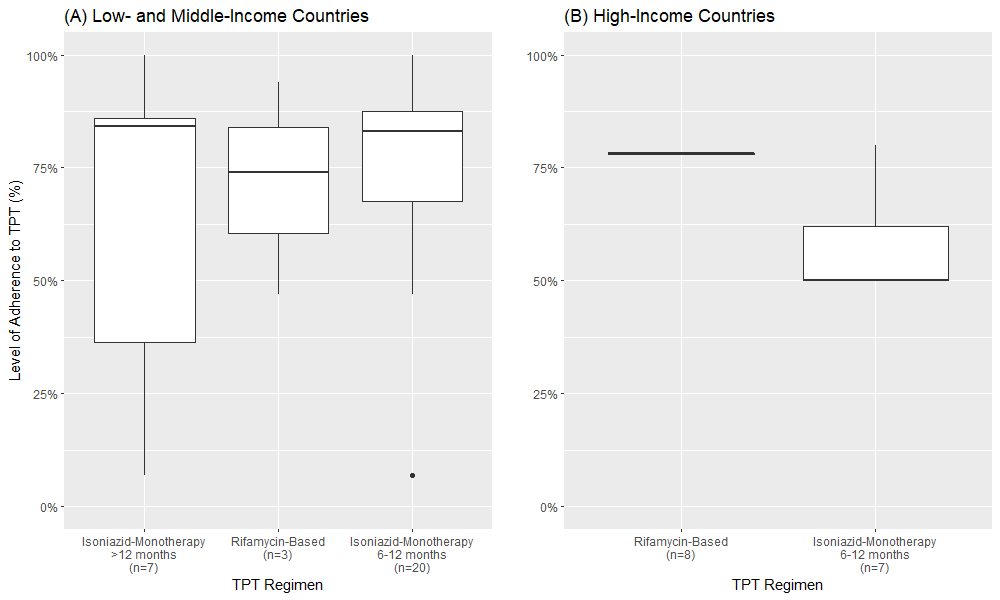


Abbreviations: TPT = tuberculosis preventive therapy


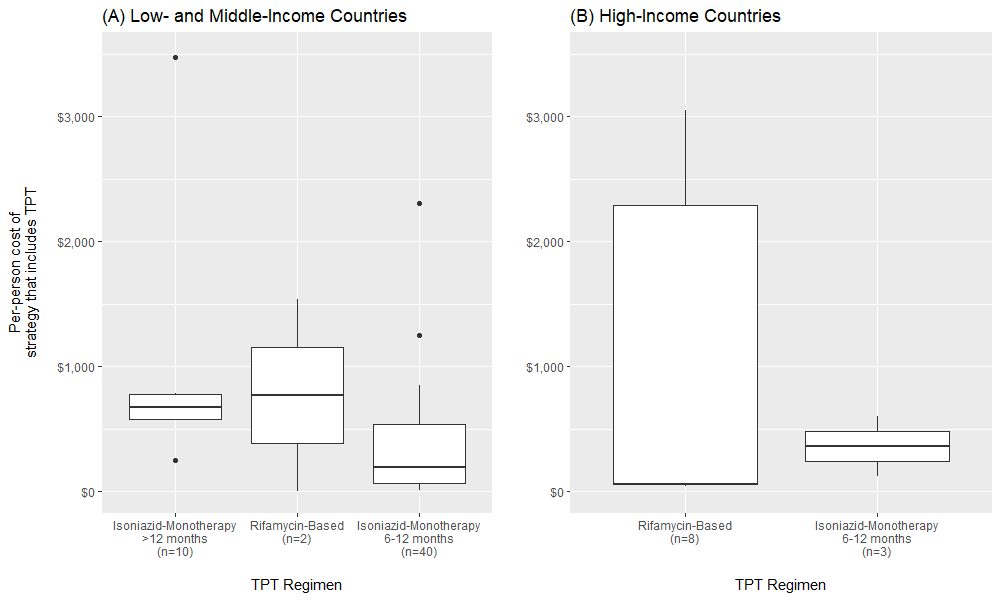


Fig G. Model outputs: comparing per-person cost of strategies that included TPT by TPT regimen category and country-level income. The thick horizontal line within each box indicates the median per-person cost of the strategy that pertains to that regimen. The dots in graph (A) and (B) represent strategies that were outliers, and found that TPT was more or less expensive than usual. Outliers are analyzed in more detail in the text. Costs displayed in this figure include program costs related to TPT delivery (drug costs, personnel costs, material costs) **as well as** costs related to TB care for those who develop active TB (drug costs, hospitalization costs, personnel costs). Rifamycin-based regimens consist of three months of isoniazid and rifampin as well as 3 months of isoniazid and rifapentine.

Abbreviations: TPT = tuberculosis preventive therapy


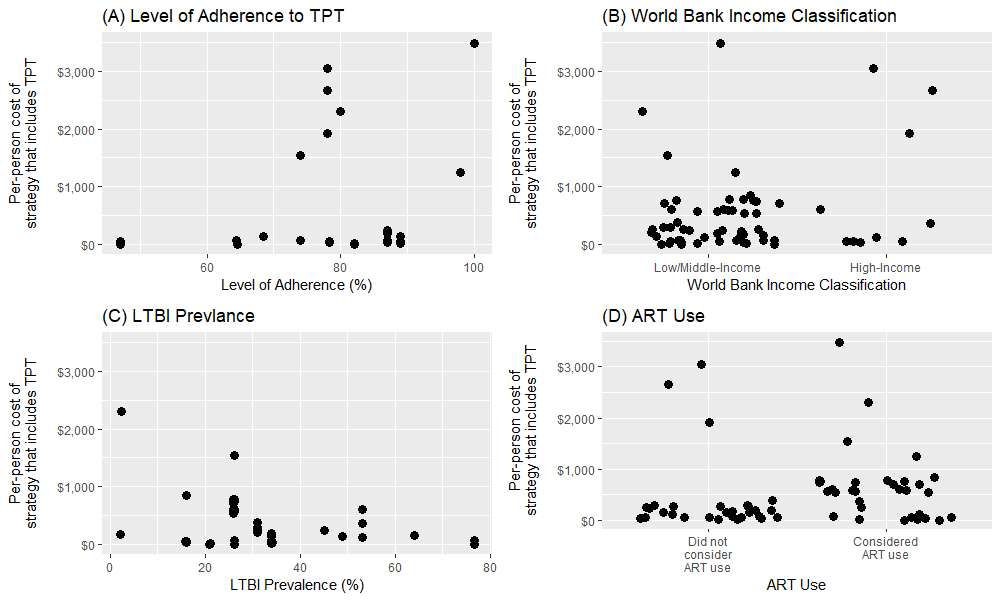


Fig H. Model inputs: select variables and their relationship to the per-person cost of strategies that included TPT. Each data point represents an individual study arm, or “strategy”. Costs displayed in this figure include program costs related to TPT delivery (drug costs, personnel costs, material costs) **as well as** costs related to TB care for those who develop active TB (drug costs, hospitalization costs, personnel costs).

Abbreviations: TPT = tuberculosis preventive therapy, LTBI = latent TB infection, ART = antiretroviral therapy


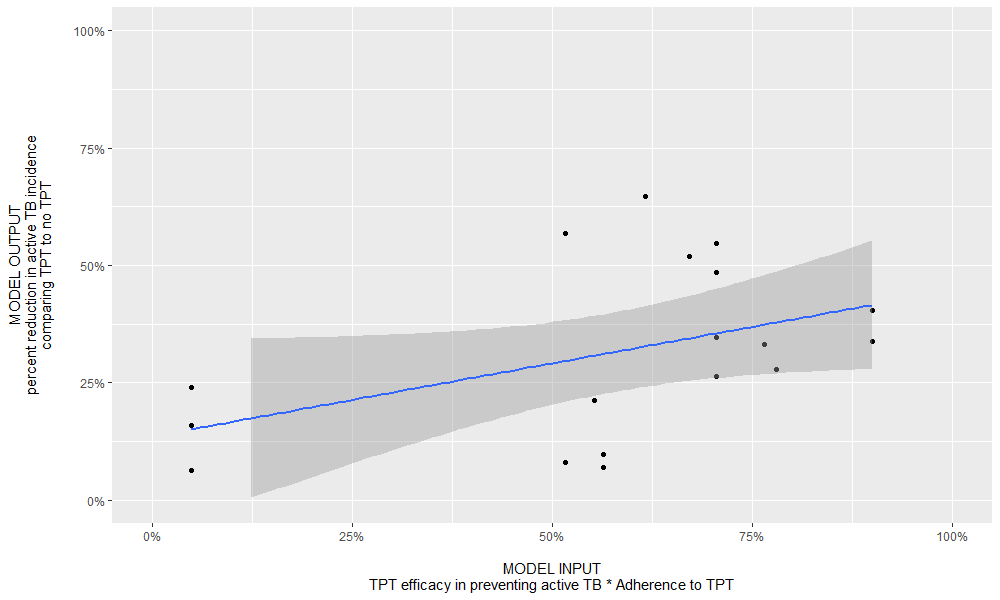


Fig I. Model inputs versus model outputs: comparing calculated effectiveness based on model inputs (efficacy x adherence) to reported effectiveness based on model outputs (percent reduction in active TB incidence). Each data point represents a study arm that had complete data for efficacy, adherence, and predicted reduction in active TB incidence comparing TPT to no TPT. There is a positive correlation between the model inputs and outputs, meaning that predicted TPT effectiveness tended to be higher where input parameters for TPT efficacy and adherence were higher.

Abbreviations: TPT = tuberculosis preventive therapy, TB = tuberculosis


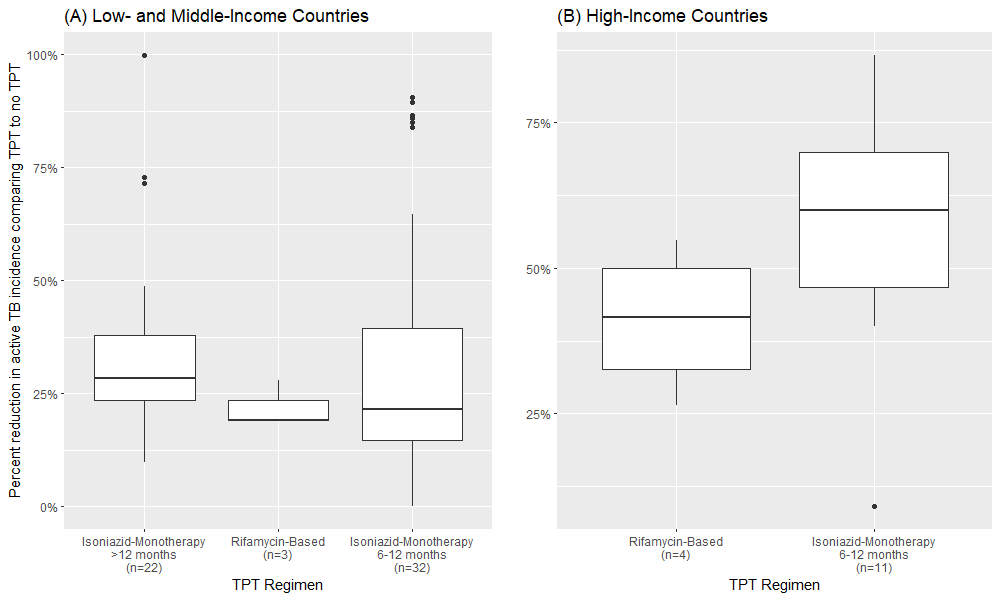


Fig J. Model outputs: comparing reduction in active TB incidence by TPT regimen category and country-level income. The thick horizontal line within each box indicates the median percent reduction in TB incidence comparing TPT to no TPT that pertains to that regimen. The dots in graph (A) represent study arms that were outliers, and found that TPT was more or less effective than usual. Outliers are analyzed in more detail in the text. Rifamycin-based regimens consist of three months of isoniazid and rifampin as well as 3 months of isoniazid and rifapentine.

Abbreviations: TB = tuberculosis, TPT = tuberculosis preventive therapy


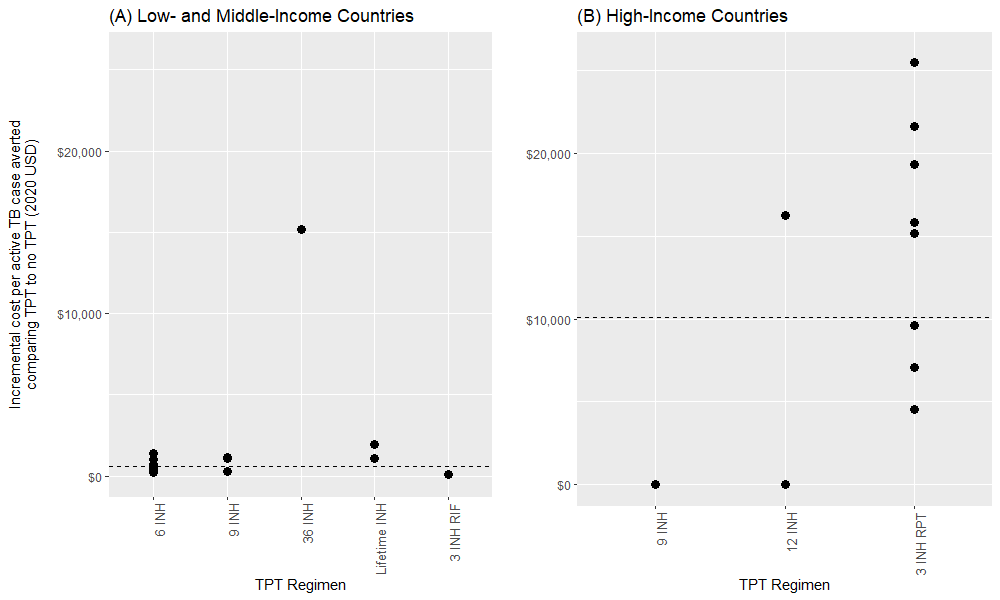


Fig K. Model outputs: comparing incremental cost per active TB case averted by country-level income and TPT regimen category. Each data point represents an individual study arm. Studies may have had more than one study arm that compared the same TPT regimen to no TPT, but using different screening strategies to indicate TPT. Such is the case with the four data points attributed to 3 INH RPT in graph (B); starting from the top, study arms employed IGRA followed by TST, IGRA alone, TST alone, and TST followed by IGRA as different screening strategies to indicate TPT.

Any data point at $0 represents a situation where TPT was cost-saving compared to no TPT. The dashed lines represent the upper limit of the cost-effectiveness thresholds in low/middle-income countries and high-income countries [62]. Study arms that fall above these thresholds, however, may still be considered cost-effective because cost-effectiveness is often based on feasibility and acceptability as well, instead of a threshold alone.

Outliers are analyzed in more detail at the end of this document.

Abbreviations: TB = tuberculosis, TPT = tuberculosis preventive therapy, TST = tuberculin skin test, IGRA = interferon gamma release assay, INH = isoniazid, RIF = rifampin, RPT = rifapentine

## Outliers

There were several studies that reported cost savings of TPT compared to no TPT [6,16,39,53]. One of these studies considered additional costs for secondary active TB cases, which were a lot higher among the portion of the cohort that did not take TPT [6]. Another study found that TPT was more expensive compare to no TPT, but the cost of averting one active TB case was cheaper than the cost of treating one active TB case, so it would lead to health system cost savings in the long run [53]. One study investigated long durations of TPT efficacy (from 6 months of protection to lifelong protection), which resulted in high numbers of active TB cases being averted, and thereby large cost savings [16]. Finally, one study predicted zero active TB cases over 5 years of follow up among individuals taking TPT at a syringe exchange program, so there were substantial cost savings in TB care [39].

Similarly, there were two studies that reported high incremental costs per active TB case averted comparing TPT to no TPT (although both studies concluded that TPT was still a cost-effective option). One of these studies considered directly observed preventive therapy (DOPT), so there were extra costs related to patient follow-up [19]. The other considered full adherence to TPT (for 36 months), thereby increasing the medication costs and overall program costs [40].

There were some outliers among effectiveness outcomes across studies. Two studies reported marked differences in TB incidence between individuals taking TPT and individuals not taking TPT. One of these studies was also an outlier among cost-effectiveness outcomes; by including long durations of protection from TPT, high numbers of active TB cases were averted, and TPT appeared to be far more beneficial than no TPT [16]. The other study in this category had a low number of predicted active TB cases overall, so the difference between TPT and no TPT cohorts was more pronounced [5]. On the other hand, there was one study that predicted a very modest impact of TPT on reducing TB incidence compared to no TPT. This study had a low prevalence of LTBI (4.5%) and only considered a 10% increase in coverage of TPT [8].

# DATA EXTRACTION FORM

**Table R**. Form used for data extraction with working example

| **PART 1. Study Characteristics** | | | | | | | | | | | |
| --- | --- | --- | --- | --- | --- | --- | --- | --- | --- | --- | --- |
| Study Design | First Author | Year of Publication | Study Setting | Baseline TB incidence | Baseline HIV prevalence | Population | Modelling Method | Analytic Horizon | TPT Regimens Included | Test Used to Indicate TPT? | Study Objective |
| ***EXAMPLES OF EXTRACTION*** | | | | | | | | | | | |
| Cost Effectiveness Analysis | Azadi, M. | 2014 | Rio de Janeiro, Brazil | 95.3 per 100,000 person-years | 10% of TB cases are co-infected with HIV | PLHIV | Decision Analysis | 20 years | 6 INH | Yes (TST) | To analyze the costs, impact on disability adjusted life years (DALY), and cost effectiveness of the THRio study |

| **PART 2. Input Parameters** | | | | | | | | | | | | | | | | | |
| --- | --- | --- | --- | --- | --- | --- | --- | --- | --- | --- | --- | --- | --- | --- | --- | --- | --- |
| Study Author | LTBI prevalence | Probability of progression to active TB | Probability of reactivation to active TB | TPT REGIMEN | Rate of TPT completion/ adherence | TPT efficacy in preventing active TB | Duration of TPT efficacy | Probability of adverse event from TPT | TST sensitivity | TST specificity | IGRA sensitivity | IGRA specificity | Cost of TPT (2020 USD) | Cost of TST (2020 USD) | Cost of IGRA (2020 USD) | Type of adverse event | Cost of adverse event (2020 USD) |
| ***EXAMPLES OF EXTRACTION*** | | | | | | | | | | | | | | | | | |
| Azadi, M. | NR |  |  | 6 INH |  | 0.87 | 20 years |  |  |  |  |  | $36.27 per unit | $32.92 per unit |  |  |  |

| **PART 3. Outcomes** | | | | | | | | | | | | | |
| --- | --- | --- | --- | --- | --- | --- | --- | --- | --- | --- | --- | --- | --- |
| Study Author | Perspective | Currency (Country & Year) | Annual Discount Rate | Strategies Compared | Cost Outcomes | | | Effectiveness Outcomes | | | Cost-Effectiveness Outcomes | | |
|  |  |  |  |  | Outcome | Strategy | Value (95% CI) | Outcome | Strategy | Value (95% CI) | Outcome | Strategy | Value (95% CI) |
| ***EXAMPLES OF EXTRACTION*** | | | | | | | | | | | | | |
| Azadi, M. | Medical Sector | 2010 USD | 3% | (1) THRio: training HIV clinics in using TST and IPT (6 months of isoniazid 300 mg and pyridoxine 25 mg daily); (2) Usual care | Median discounted cost including intervention implementation, diagnosis, follow-up, and therapy in 100 patients followed 20 years | 1 | $9,748 ($9,530 - $10,078) | Discounted DALYs per 100 patients followed 20 years | 1 | 580.88 | Incremental cost per DALY averted | (1) vs. (2) | $2,273 ($1,179 - $3,135) |
|  |  |  |  |  |  |  |  |  | 2 | 582.02 |  |  |  |
|  |  |  |  |  |  |  |  | DALYs lost by 100 people over 20 years (not discounted) | 1 | 821 |  |  |  |
|  |  |  |  |  |  |  |  |  | 2 | 822 |  |  |  |
|  |  |  |  |  |  | 2 | $6461 ($6,278 - $6,783) | TB Deaths | 1 | 1.1 | Incremental cost per TB case averted | (1) vs. (2) | $2,191 |
|  |  |  |  |  |  |  |  |  | 2 | 1.3 |  |  |  |
|  |  |  |  |  |  |  |  | Active TB Cases | 1 | 10 |  |  |  |
|  |  |  |  |  |  |  |  |  | 2 | 11.5 |  |  |  |

| **PART 4. Assumptions** | | | | | |
| --- | --- | --- | --- | --- | --- |
| Study Author | Assumption Related To: | If applicable, assumed value (point estimate) | Lower bound of 95% CI | Upper bound of 95% CI | Describe assumption |
| ***EXAMPLES OF EXTRACTION*** | | | | | |
| Azadi, M. | Duration of protection due to TPT | 20 years | NR | NR | IPT remains effective for 20 years |
|  | TPT efficacy in preventing active disease |  |  |  | Only effect of IPT would be through a reduction in TB incidence (i.e., no ancillary benefits on mortality beyond a reduction in strictly measured incident TB cases) - loosened this assumption in sensitivity analysis |
|  | Utility Scores |  |  |  | State of TB/HIV disability lasted for one year before reverting to the disability state of chronic stable HIV |

| **PART 5. Sensitivity Analysis** | | | | | | | |
| --- | --- | --- | --- | --- | --- | --- | --- |
| Study | Type of Sensitivity Analysis (One-way, Two-way, Multi-way) | Name of key outcome driver (parameter) identified by sensitivity analysis | LOW parameter value | HIGH parameter value | Outcome | Outcome value that results from LOW parameter value | Outcome value that results from HIGH parameter value |
| ***EXAMPLES OF EXTRACTION*** | | | | | | | |
| Azadi, M. | One-way and probabilistic/Monte Carlo | Clinical training cost | $6,655 | NR | Incremental cost per DALY averted | $3,075 | $2,055 |
|  |  | Disability weight for TB-HIV | 10 points worse than HIV | NR |  | $2,352 | $2,873 |
|  |  | THRio's hazard ratio | 0.7 | 0.97 |  | $2,486 | $2,650 |

# REFERENCES

1. Awoke TD, Kassa SM. Optimal control strategy for TB-HIV/AIDS Co-infection model in the presence of behaviour modification. Processes. 2018 May;6(5):48.
2. Azadi M, Bishai DM, Dowdy DW, Moulton LH, Cavalcante S, Saraceni V, Pacheco AG, Cohn S, Chaisson RE, Durovni B, Golub JE. Cost-effectiveness of tuberculosis screening and isoniazid treatment in the TB/HIV in Rio (THRio) Study. The International journal of tuberculosis and lung disease. 2014 Dec 1;18(12):1443-8.
3. Bacaër N, Ouifki R, Pretorius C, Wood R, Williams B. Modeling the joint epidemics of TB and HIV in a South African township. Journal of mathematical biology. 2008 Oct 1;57(4):557.
4. Bachmann MO. Effectiveness and cost effectiveness of early and late prevention of HIV/AIDS progression with antiretrovirals or antibiotics in Southern African adults. AIDS care. 2006 Feb 1;18(2):109-20.
5. Basu S, Maru D, Poolman E, Galvani A. Primary and secondary tuberculosis preventive treatment in HIV clinics: simulating alternative strategies. The International journal of tuberculosis and lung disease. 2009 May 1;13(5):652-8.
6. Bell JC, Rose DN, Sacks HS. Tuberculosis preventive therapy for HIV-infected people in sub-Saharan Africa is cost-effective. Aids. 1999 Aug 20;13(12):1549-56.
7. Brewer TF, Heymann SJ, Colditz GA, Wilson ME, Auerbach K, Kane D, Fineberg HV. Evaluation of tuberculosis control policies using computer simulation. JAMA. 1996 Dec 18;276(23):1898-903.
8. Brewer TF, Heymann SJ, Krumplitsch SM, Wilson ME, Colditz GA, Fineberg HV. Strategies to decrease tuberculosis in US homeless populations: a computer simulation model. Jama. 2001 Aug 15;286(7):834-42.
9. Burgos JL, Kahn JG, Strathdee SA, Valencia-Mendoza A, Bautista-Arredondo S, Laniado-Laborin R, Castañeda R, Deiss R, Garfein RS. Targeted screening and treatment for latent tuberculosis infection using QuantiFERON®-TB Gold is cost-effective in Mexico. The International journal of tuberculosis and lung disease. 2009 Aug 1;13(8):962-8.
10. Cohen T, Lipsitch M, Walensky RP, Murray M. Beneficial and perverse effects of isoniazid preventive therapy for latent tuberculosis infection in HIV–tuberculosis coinfected populations. Proceedings of the National Academy of Sciences. 2006 May 2;103(18):7042-7.
11. Currie CS, Floyd K, Williams BG, Dye C. Cost, affordability and cost-effectiveness of strategies to control tuberculosis in countries with high HIV prevalence. BMC Public Health. 2005 Dec 1;5(1):130.
12. de Siqueira Filha NT, Legood R, Rodrigues L, Santos AC. The economic burden of tuberculosis and latent tuberculosis in people living with HIV in Brazil: a cost study from the patient perspective. Public health. 2018 May 1;158:31-6.
13. de Siqueira-Filha NT, de Albuquerque MD, Rodrigues LC, Legood R, Santos AC. Economic burden of HIV and TB/HIV coinfection in a middle-income country: a costing analysis alongside a pragmatic clinical trial in Brazil. Sexually transmitted infections. 2018 Sep 1;94(6):463-9.
14. Dowdy DW, Golub JE, Saraceni V, Moulton LH, Cavalcante SC, Cohn S, Pacheco AG, Chaisson RE, Durovni B. Impact of isoniazid preventive therapy for HIV-infected adults in Rio de Janeiro, Brazil: an epidemiological model. Journal of acquired immune deficiency syndromes (1999). 2014 Aug 15;66(5):552.
15. Dye C, Glaziou P, Floyd K, Raviglione M. Prospects for tuberculosis elimination. Annual review of public health. 2013 Mar 20;34.
16. Foster S, Godfrey-Faussett P, Porter J. Modelling the economic benefits of tuberculosis preventive therapy for people with HIV: the example of Zambia. Aids. 1997 Jun 11;11(7):919-25.
17. Freiman JM, Jacobson KR, Muyindike WR, Horsburgh CR, Ellner JJ, Hahn JA, Linas BP. Isoniazid Preventive Therapy for People with HIV who are Heavy Alcohol Drinkers in High TB/HIV Burden Countries: A Risk-Benefit Analysis. Journal of acquired immune deficiency syndromes (1999). 2018 Apr 1;77(4):405.
18. Gilbert JA, Shenoi SV, Moll AP, Friedland GH, Paltiel AD, Galvani AP. Cost-effectiveness of community-based TB/HIV screening and linkage to care in rural South Africa. PLoS One. 2016 Dec 1;11(12):e0165614.
19. Gourevitch MN, Alcabes P, Wasserman WC, Arno PS. Cost-effectiveness of directly observed chemoprophylaxis of tuberculosis among drug users at high risk for tuberculosis. The International Journal of Tuberculosis and Lung Disease. 1998 Jul 1;2(7):531-40.
20. Gupta S, Abimbola T, Suthar AB, Bennett R, Sangrujee N, Granich R. Cost-effectiveness of the Three I's for HIV/TB and ART to prevent TB among people living with HIV. The International journal of tuberculosis and lung disease. 2014 Oct 1;18(10):1159-65.
21. Guwatudde D, Debanne SM, Diaz M, King C, Whalen CC. A re-examination of the potential impact of preventive therapy on the public health problem of tuberculosis in contemporary sub-Saharan Africa. Preventive medicine. 2004 Nov 1;39(5):1036-46.
22. Hausler HP, Sinanovic E, Kumaranayake L, Naidoo P, Schoeman H, Karpakis B, Godfrey-Faussett P. Costs of measures to control tuberculosis/HIV in public primary care facilities in Cape Town, South Africa. Bulletin of the World Health Organization. 2006 Jul 10;84:528-36.
23. Heymann SJ. Modelling the efficacy of prophylactic and curative therapies for preventing the spread of tuberculosis in Africa. Transactions of the Royal Society of Tropical Medicine and Hygiene. 1993 Jul 1;87(4):406-11.
24. Houben RM, Sumner T, Grant AD, White RG. Ability of preventive therapy to cure latent Mycobacterium tuberculosis infection in HIV-infected individuals in high-burden settings. Proceedings of the National Academy of Sciences. 2014 Apr 8;111(14):5325-30.
25. Johnson KT, Churchyard GJ, Sohn H, Dowdy DW. Cost-effectiveness of preventive therapy for tuberculosis with isoniazid and rifapentine versus isoniazid alone in high-burden settings. Clinical Infectious Diseases. 2018 Sep 14;67(7):1072-8.
26. Jordan TJ, Lewit EM, Montgomery RL, Reichman LB. Isoniazid as preventive therapy in HIV-infected intravenous drug abusers: a decision analysis. JAMA. 1991 Jun 12;265(22):2987-91.
27. Kapoor S, Gupta A, Shah M. Cost-effectiveness of isoniazid preventive therapy for HIV-infected pregnant women in India. The International Journal of Tuberculosis and Lung Disease. 2016 Jan 1;20(1):85-92.
28. Kendall EA, Azman AS, Maartens G, Boulle A, Wilkinson RJ, Dowdy DW, Rangaka MX. Projected population-wide impact of antiretroviral therapy-linked isoniazid preventive therapy in a high-burden setting. AIDS (London, England). 2019 Mar 1;33(3):525.
29. Kim HY, Hanrahan CF, Martinson N, Golub JE, Dowdy DW. Cost-effectiveness of universal isoniazid preventive therapy among HIV-infected pregnant women in South Africa. The International Journal of Tuberculosis and Lung Disease. 2018 Dec 1;22(12):1435-42.
30. Kowada A. Cost effectiveness of interferon-γ release assay for TB screening of HIV positive pregnant women in low TB incidence countries. Journal of Infection. 2014 Jan 1;68(1):32-42.
31. Kunkel A, Crawford FW, Shepherd J, Cohen T. Benefits of continuous isoniazid preventive therapy may outweigh resistance risks in a declining TB/HIV co-epidemic. AIDS (London, England). 2016 Nov 13;30(17):2715.
32. Linas BP, Wong AY, Freedberg KA, Horsburgh Jr CR. Priorities for screening and treatment of latent tuberculosis infection in the United States. American journal of respiratory and critical care medicine. 2011 Sep 1;184(5):590-601.
33. Long EF, Vaidya NK, Brandeau ML. Controlling co-epidemics: analysis of HIV and tuberculosis infection dynamics. Operations research. 2008 Dec;56(6):1366-81.
34. Maheswaran H, Barton P. Intensive case finding and isoniazid preventative therapy in HIV infected individuals in Africa: economic model and value of information analysis. PLoS One. 2012 Jan 23;7(1):e30457.
35. Marx FM, Yaesoubi R, Menzies NA, Salomon JA, Bilinski A, Beyers N, Cohen T. Tuberculosis control interventions targeted to previously treated people in a high-incidence setting: a modelling study. The Lancet Global Health. 2018 Apr 1;6(4):e426-35.
36. Masobe P, Lee T, Price M. Isoniazid prophylactic therapy for tuberculosis in HIV-seropositive patients-a least-cost analysis. South African Medical Journal. 1995;85(2):75-81.
37. Mills HL, Cohen T, Colijn C. Community-wide isoniazid preventive therapy drives drug-resistant tuberculosis: a model-based analysis. Science translational medicine. 2013 Apr 10;5(180):180ra49-.
38. Mills HL, Cohen T, Colijn C. Modelling the performance of isoniazid preventive therapy for reducing tuberculosis in HIV endemic settings: the effects of network structure. Journal of the royal society interface. 2011 Oct 7;8(63):1510-20.
39. Perlman DC, Gourevitch MN, Trinh C, Salomon N, Horn L, Des Jarlais DC. Cost-effectiveness of tuberculosis screening and observed preventive therapy for active drug injectors at a syringe-exchange program. Journal of Urban Health. 2001 Sep;78(3):550-67.
40. Pho MT, Swaminathan S, Kumarasamy N, Losina E, Ponnuraja C, Uhler LM, Scott CA, Mayer KH, Freedberg KA, Walensky RP. The cost-effectiveness of tuberculosis preventive therapy for HIV-infected individuals in southern India: a trial-based analysis. PloS one. 2012 Apr 30;7(4):e36001.
41. Rhines AS, Feldman MW, Bendavid E. Modeling the implementation of population-level isoniazid preventive therapy for tuberculosis control in a high HIV-prevalence setting. AIDS (London, England). 2018 Sep 24;32(15):2129.
42. Rose DN, Schechter CB, Sacks HS. Preventive medicine for HIV-infected patients. Journal of general internal medicine. 1992 Nov 1;7(6):589-94.
43. Rose DN. Benefits of screening for latent Mycobacterium tuberculosis infection. Archives of Internal Medicine. 2000 May 22;160(10):1513-21.
44. Samandari T, Bishai D, Luteijn M, Mosimaneotsile B, Motsamai O, Postma M, Hubben G. Costs and consequences of additional chest x-ray in a tuberculosis prevention program in Botswana. American journal of respiratory and critical care medicine. 2011 Apr 15;183(8):1103-11.
45. Sawert H, Girardi E, Antonucci G, Raviglione MC, Viale P, Ippolito G. Preventive therapy for tuberculosis in HIV-infected persons: analysis of policy options based on tuberculin status and CD4+ cell count. Archives of internal medicine. 1998 Oct 26;158(19):2112-21.
46. Shayo GA, Chitama D, Moshiro C, Aboud S, Bakari M, Mugusi F. Cost-Effectiveness of isoniazid preventive therapy among HIV-infected patients clinicaly screened for latent tuberculosis infection in Dar es Salaam, Tanzania: A prospective Cohort study. BMC public health. 2018 Dec 1;18(1):35.
47. Shrestha RK, Mugisha B, Bunnell R, Mermin J, Hitimana-Lukanika C, Odeke R, Madra P, Adatu F, Blandford JM. Cost-effectiveness of including tuberculin skin testing in an IPT program for HIV-infected persons in Uganda. The International Journal of Tuberculosis and Lung Disease. 2006 Jun 1;10(6):656-62.
48. Shrestha RK, Mugisha B, Bunnell R, Mermin J, Odeke R, Madra P, Hitimana-Lukanika C, Adatu-Engwau F, Blandford JM. Cost-utility of tuberculosis prevention among HIV-infected adults in Kampala, Uganda. The International Journal of Tuberculosis and Lung Disease. 2007 Jul 1;11(7):747-54.
49. Smith T, Samandari T, Abimbola T, Marston B, Sangrujee N. Cost-effectiveness of antiretroviral therapy and Isoniazid prophylaxis to reduce tuberculosis and death in people living with HIV in Botswana. Journal of acquired immune deficiency syndromes (1999). 2015 Nov 1;70(3):e84.
50. Snyder DC, Paz EA, Mohle-Boetani JC, Fallstad R, Black RL, Chin DP. Tuberculosis prevention in methadone maintenance clinics: effectiveness and cost-effectiveness. American journal of respiratory and critical care medicine. 1999 Jul 1;160(1):178-85.
51. Sterling TR, Brehm WT, Moore RD, Chaisson RE. Tuberculosis vaccination versus isoniazid preventive therapy: A decision analysis to determine the preferred strategy of tuberculosis prevention in HIV-infected adults in the developing world. The International Journal of Tuberculosis and Lung Disease. 1999 Mar 1;3(3):248-54.
52. Sumner T, Houben RM, Rangaka MX, Maartens G, Boulle A, Wilkinson RJ, White RG. Post-treatment effect of isoniazid preventive therapy on tuberculosis incidence in HIV-infected individuals on antiretroviral therapy. Aids. 2016 May 15;30(8):1279-86.
53. Sutton BS, Arias MS, Chheng P, Eang MT, Kimerling ME. The cost of intensified case finding and isoniazid preventive therapy for HIV-infected patients in Battambang, Cambodia. The International journal of tuberculosis and lung disease. 2009 Jun 1;13(6):713-8.
54. Tasillo A, Salomon JA, Trikalinos TA, Horsburgh CR, Marks SM, Linas BP. Cost-effectiveness of testing and treatment for latent tuberculosis infection in residents born outside the United States with and without medical comorbidities in a simulation model. JAMA internal medicine. 2017 Dec 1;177(12):1755-64.
55. Terris-Prestholt F, Kumaranayake L, Ginwalla R, Ayles H, Kayawe I, Hillery M, Godfrey-Faussett P. Integrating tuberculosis and HIV services for people living with HIV: costs of the Zambian ProTEST Initiative. Cost Effectiveness and Resource Allocation. 2008 Dec 1;6(1):2.
56. Vynnycky E, Sumner T, Fielding KL, Lewis JJ, Cox AP, Hayes RJ, Corbett EL, Churchyard GJ, Grant AD, White RG. Tuberculosis control in South African gold mines: mathematical modeling of a trial of community-wide isoniazid preventive therapy. American journal of epidemiology. 2015 Apr 15;181(8):619-32.
57. Yan I, Bendavid E, Korenromp EL. Antiretroviral treatment scale-up and tuberculosis mortality in high TB/HIV burden countries: an econometric analysis. PloS one. 2016 Aug 18;11(8):e0160481.
58. Ferguson O, Jo Y, Pennington J, Johnson K, Chaisson RE, Churchyard G, Dowdy D. Cost‐effectiveness of one month of daily isoniazid and rifapentine versus three months of weekly isoniazid and rifapentine for prevention of tuberculosis among people receiving antiretroviral therapy in Uganda. J Int AIDS Soc. 2020 Oct;23(10):e25623.
59. Hsieh YL, Jahn A, Menzies NA, Yaesoubi R, Salomon JA, Girma B, Gunde L, Eaton JW, Auld A, Odo M, Kiyiika CN. Evaluation of 6-Month Versus Continuous Isoniazid Preventive Therapy for Mycobacterium tuberculosis in Adults Living With HIV/AIDS in Malawi. J Acq Immun Def Synd. 2020 Dec 15;85(5):643-50.
60. Jo Y, Shrestha S, Gomes I, Marks S, Hill A, Asay G, Dowdy D. Model-Based Cost-Effectiveness of State-level Latent Tuberculosis Interventions in California, Florida, New York and Texas. Clin Infect Dis. 2020 Jun 25.
61. Mandal S, Bhatia V, Sharma M, Mandal PP, Arinaminpathy N. The potential impact of preventive therapy against tuberculosis in the WHO South-East Asian Region: a modelling approach. BMC Med. 2020 Dec;18(1):1-0.
62. Woods B, Revill P, Sculpher M, Claxton K. Country-level cost-effectiveness thresholds: initial estimates and the need for further research. Value in Health. 2016 Dec 1;19(8):929-35.
