# Supplementary material for: Economic and modeling evidence for tuberculosis preventive therapy among people living with HIV: A systematic review and meta-analysis
Source: PLoS Med. 2021 Sep 14;18(9):e1003712. doi: 10.1371/journal.pmed.1003712 (PMC8439468; doi:10.1371/journal.pmed.1003712)
Supplement: Attachment — Submitted filename: uppal.pdf [file pmed.1003712.s004.pdf]

This paper reports a systematic review of studies of tuberculosis preventive therapy in people living with HIV.

I was asked for a statistical report and I interpret that to include all aspects of the design and conduct of the study. I note that this is one of a collection of three papers and I have previously reviewed the other two.

Points of detail

Page 6 If the authors did wish to weight their meta–analysis then one possible choice of weights would be some function of the sample size. I think technically they have done a meta–regression but not the usual inverse–variance weighted one but I think their description is fine.

Page 6 Selecting a subset of variables in a way driven by the data leads to a model which is unlikely to replicate (Babyak, 2004). Screening variables to enter the model has been criticised too (Sun et al., 1996) In fact the results presented in Table S9 do not match my understanding of what the authors state here in the methods as there they seem to have included all the categorical covariates and then added each of four continuous ones alone. If that is what was done it seems to me more defensible than a full forward selection approach.

Table 3c Why are there seven continuous variables here but only five in 3a and 3b?

Figures I think it might be better for Figures 2, 3 and 4 to use unfilled symbols as it is hard to tell whether over–printing is spoiling the visual impact. An alternative would be adding some horizontal jitter. The same applies to Figure S5.

Some of the continuous predictors seem to me to suggest that they are ecological in nature. For instance I assume that adherence means being enrolled in a study with that level of average adherence, not personally adhering. If that is correct then perhaps a mention in limitations might be in order.

Point of more substance

One possible explanation of the very convincing conclusion that all studies report the same effect, at least qualitatively, is that anyone who found the converse would struggle to find an outlet for it. Are the authors convinced that their search strategy can rule this out? There is no mention of an

explicit language restriction but the choice of bibliographic databases has a bias towards English language publications (Pilkington et al., 2005).

Summary

No major concerns.

Michael Dewey

References

M A Babyak. What you see may not be what you get: a brief, nontechnical introduction to overfitting in regression–type models. *Psychosomatic Medicine*, 66:411–421, 2004.

K Pilkington, A Boshnakova, M Clarke, and J Richardson. 'No language restrictions' in database searches: what does this really mean? *The Journal of Alternative and Complementary Medicine*, 11:205–207, 2005.

G-W Sun, T L Shook, and G L Kay. Inappropriate use of bivariable analysis to screen risk factors for use in multivariable analysis. *Journal of Clinical Epidemiology*, 49:907–916, 1996.
