# Supplementary material for: Economic and modeling evidence for tuberculosis preventive therapy among people living with HIV: A systematic review and meta-analysis
Source: PLoS Med. 2021 Sep 14;18(9):e1003712. doi: 10.1371/journal.pmed.1003712 (PMC8439468; doi:10.1371/journal.pmed.1003712)
Supplement: Attachment — Submitted filename: CostEffectiveness TPTinHIV Reviewer Responses.docx [file pmed.1003712.s005.docx]

Dear Dr Turner,

We appreciate the opportunity to address comments and suggestions and resubmit our manuscript to PLOS Medicine. In each point-by-point response, we have provided exact locations in our manuscript (page and line number) where revised text may be found. Note that we numbered the reviewers’ comments to make it easier to follow our responses, especially when different reviewers raised the same point - we responded only once. As per the editor’s request, these changes are not tracked or highlighted; we are submitting a clean version of our manuscript as the main article. A marked up version is available in the submission as well, in which all changes are indicated in red text. Note that the Line numbers below relate to the CLEAN version. Also note that we are submitting a figures file in WORD.; this has all figures, plus figure titles and legends - for ease of reading by reviewers/editors. But high quality figure files are also attached (.tif files).

We believe the revised manuscript addresses all reviewers comments, and hope this revised version is now acceptable for publication in PLOS Medicine.

Aashna Uppal and Samiha Rahman, on behalf of all authors.

**Requests from the editors:**

Did this study have a protocol or prespecified analysis plan, and was it registered prior to being carried out?

Response: Before beginning the study, we submitted the study protocol to PROSPERO; it was registered on May 7^th^ 2020 and the registration number is CRD42020187934. We have included the registration number at the beginning of the methods section, on Page 5, Line 126.

Please update the search to the end of 2020, say.

Response: We have applied the search strategy to include publications in each of the selected databases until December 31^st^, 2020. We have updated this methodological detail in the text on Page 5, Line 128-129.

Please remove the information on funding from the title page. In the event of publication, this information will appear in the article metadata, via entries in the submission form.

Response**:** We have removed this information from the title page and have now included it at the end of the document, Page 27, Lines 530-531.

Please make that "data were" in the abstract; just before that, a duplicate full point needs to be removed.

Response: We have made it “data were” in the abstract. Page 2, Line 39.

We suggest adding an additional sentence, say, to the abstract to summarize study designs, settings and other study characteristics such as regimens.

Response: We have added the following to our abstract: “41 were set in low- and middle-income countries (LMICs), while 12 were set in high-income countries (HICs); 2 were set in both. Most studies considered isoniazid-based regimens 6-12 months long (n = 45), or longer than 12 months (n = 11).” Page 2, Lines 44-47.

Please add a new final sentence to the "Methods and findings" subsection of your abstract, which should quote 2-3 of the study's main limitations.

Response: We have added the following to the end of the Methods and Findings section of our abstract: “Key limitations of this review included the heterogeneity of input parameters and assumptions from included studies, which limited pooling of effect estimates, inconsistent reporting of model parameters, which limited sample sizes of quantitative analyses, and database bias toward English publications.” Page 2, Line 61-64.

After the abstract, please add a new, accessible "author summary" section in non-identical prose. You may find it helpful to consult one or two recent research articles published in PLOS Medicine to get a sense of the preferred style.

Response: We have added an accessible author summary right after the abstract. This summarizes why the study was done, what our main methods were, what we found, and what our findings mean. Page 3, Line 71-99.

Please add a completed checklist for the most appropriate reporting guideline as a supplementary document, labelled "S1_PRISMA_Checklist" or similar, referred to in your Methods section. In the checklist, individual items should be referred to by section (e.g., "Methods") and paragraph number rather than by line or page numbers, as the latter generally change in the event of publication.

Response: Thank you for pointing this out. We have attached a supplementary document, labelled “PRISMA_Checklist”.

Please make that "low- and middle-income countries" throughout.

Response: Thank you for this suggestion. We have made sure to stay consistent and have used this terminology throughout.

Please ensure that journal names are abbreviated consistently in your reference list. Please add "U S A" to the journal name for reference 18 and any other relevant entries.

Response: We have added USA to the journal name for reference 2, 18 and 32, and have abbreviated all journal names in our reference list.

**Reviewer #1:**

The authors conducted a systematic review on economic and modeling evidence for TB preventive therapy in PLHIV. This is an important area of research as TPT in both the general and PLHIV populations is an important early intervention for TB control and prevention. As with many past systematic review studies carried out by the authors' TB research group, this study carries forward the same basic strengths in thoroughness and principle-based systematic assessment of the literature in the research topic. However, this reviewer finds that this manuscript lacks clear descriptions in some of the analytic methods used and in-depth discussions on key issues in the heterogeneity that exists across the studies. Compared to the complexities of the topics that the authors investigated in this study, this reviewer finds the author's approach to writing the manuscript have been a bit to concise, which makes some of the study's findings difficult to follow (i.e. the richness of the information captured in this study are not adequately reflected in the writing).

Response: Thank you for your comments. We have described in more detail the analytic methods, key results, and heterogeneity of model parameter values. Specific actions taken are described in detail in the point by point responses below.

(1) Therefore, this reviewer has following two key improvements to enhance both the interpretation and the content of the manuscript:

The authors state in their objective that this study was carried out to "systematically review" and "synthesize the costs, cost-effectiveness, as well as risks and effect on TB morbidity and mortality associated with TPT provided to PLHIV." This reviewer finds that in general, this manuscript leaves it up to the readers to synthesize many of the important findings (e.g. the highly heterogenous nature of data, methods, and outcomes across the study) and understand what need to improve this issue for future studies. These can be improved by looking at study level differences (e.g. modeling techniques, costing methods, validity of parameter values) within a larger groups of studies (e.g. model-based studies: decision analysis vs. transmission) as this would be very difficult to do across all studies (hence the highly heterogenous nature of study methods and use of parameters)

Response: We appreciate this suggestion. We have now described study heterogeneity at the beginning of results - study characteristics and quality assessment section; “Study characteristics varied widely and input parameters included in modelling studies were clinically heterogeneous.” on Page 8, Line 203-204. As well, the beginning of our results section is divided as follows: description of effectiveness results, description of cost results, and description of cost-effectiveness results. We have described studies that fall into each of these categories in terms of modelling methods, parameter values, and costing methods (where applicable) to illustrate the extent of heterogeneity, as suggested. What follows is an example of what we have added to the cost results section: “Of the 38 unique strategies within studies that reported cost outcomes, 9 obtained cost parameters exclusively from secondary data sources, while 10 employed empiric costing methods, gathering cost parameters exclusively from primary data sources (Table 1). Of those that undertook empiric costing or a combination of secondary and empiric costing, 15 studies included costs associated with TPT implementation. Most of these 38 studies were modelling studies that excluded transmission (n = 30), while a minority were transmission modelling studies (n = 4) or analyses conducted alongside clinical trials or observational studies (n = 4). Within these 38 studies, parameters differed widely; TPT efficacy in averting active TB ranged from 11% to 100%, time horizon ranged from 1 to 100 years, and LTBI prevalence ranged from 3% to 100%.” (Page 12, Lines 261-268). We have added a similar description of included studies to the effectiveness section (Page 11, Lines 242-246) and cost-effectiveness section (Page 13, Lines 289-292).

(2) Costs are listed as one of the key topic areas that were considered for the main objective. However, the authors limit their assessment on this at high-level discussion. Given that costs parameters and estimates are equal component in cost-effectiveness analyses (and the sole objective of cost analyses studies), it will be important to dedicate a section within this manuscript to describe how studies differ on their methods in evaluating (and or the use of) costs of TPT (most important) and other health services included in their assessment (e.g. top down vs. bottom-up, or use of more empiric approach vs. referenced or assumed cost estimates)

Response: Thank you for pointing this out. While Figure 3 in the original manuscript had illustrated cost results, we have added additional results on costs. We have added to Table 1, and in the results section, the types of costing methods employed in studies; see response to Reviewer 1, Comment 1. We have also taken a closer look at whether downstream health systems costs associated with TB care are included in the overall costs of strategies modelling TPT compared to no TPT. We draw on data used to created Figure 3; “The strategies included in Figure 3 (n = 63) compared TPT to no TPT, and included the downstream health systems costs related to active TB care. The median per person cost was $299 (interquartile range $73 to $756) among these strategies. 6 other strategies also reported per person costs for TPT, but excluded downstream health systems costs related to active TB care. The median per person cost was $148 (interquartile range $117 to $579) among these strategies.” (Page 12-13, Lines 268-275)

Also, following are section and line-specific comments which should guide the authors in addressing the two issues raised above:

**A. Introduction**

(3) Line 80-81: Transmission modeling and cost-effectiveness studies only cover part of the key evidence that are needed to improve TPT coverage and service delivery. Please add.

Response: We have added this; “Transmission modelling and cost-effectiveness studies can provide part of the key evidence that are needed to improve TPT coverage and service delivery”. Page 4, Line 118-119.

**B. Methods**

(4) Line 95-96: Can authors expand on what types of economic evaluations were considered (e.g. cost analysis, budget impact analyses, etc.)

Response: We considered cost analyses, econometric analyses and cost-effectiveness analyses. We have added this on Page 5, Lines 138-139.

(5) Line 111-112: What does the author mean by "at least one key input parameters"? If study provided appropriate source for one parameter but used assumptions for rest, would this study deem to make the mark for quality? Also, how did the authors determine appropriateness of the source?
 
Response: We considered an appropriate source to be a systematic review or meta-analysis, with some exceptions; see Table S2 for details (Page 6, Line 157-158). Table S2 lists an appropriate source as: Uses a systematic review or meta-analysis to inform the following input parameters included in the model, OR, uses local trial data (studies published before 1999 are exempted from this criteria for TPT related parameters because no reviews specific to TPT efficacy in PLHIV existed prior to that year). We have also elaborated on our rationale for making this an essential criterion, in the appendix: Criterion #1 was deemed essential because descriptions of “study arms” or “strategies” are key to being able to tease apart effects of TPT among outcomes, and criterion #3 was deemed essential because a model’s outcomes are only of high-quality if the model’s input parameters are of high quality. Indeed, by definition, if a study had one input parameters that came from an appropriate source and others that did not, it would be considered to be of high quality, as long as it also met the other essential checklist item (i.e. included a clear description of the interventions under evaluation)

(6) Line 136-137: also costs are incremental (i.e. provision of TPT would increase costs compared to the status quo)

Response: Indeed, all costs are incremental compared to no TPT. We have, however, revised our analyses to focus on incremental net monetary benefit in lieu of incremental cost. As such, this line has been removed.

(7) Line 140-143: Can authors provide more descriptions as to why forward selection was chosen over other methods (e.g. all-in vs. backwards elimination vs. bidirectional elimination vs. score comparison)? This reviewer is not asking to perform and compare these model selection methods but is asking the authors provide one to two short statements on the rationale for choosing forward selection method (for the benefit of the readers).

General comment regarding description of the regression method: This reviewer feels that the authors' minimalistic description of the regression analyses that were performed for the study makes it difficult to connect the dots across data and information presented in Tables S8, 3a, b, & c, and the texts in the methods section. In short, readers are having to juggle across various parts of the manuscript to truly understand what was exactly done (e.g. there were three main regression outputs assessed study level determinants on 1. reduction in active TB; 2. Reduction in TB-related mortality; and 3. ICER estimates for TPT (vs. no TPT). Following is an example of the disconnect this reviewer experiencing when reading through the text/tables/figures: It's clear to this reviewer than the regression output is either change in percent reduction of mortality (vs. reference standard) or ICER (measured as cost per active TB averted), but it's unclear how number of strategies were used in the regression analyses.

(8) Suggestion: Can authors provide a bit more description on how the regression analysis was conducted? For lay readers, it would be helpful to spell out the equation and/or text to enhance the understanding of how data in Table S8 was assessed to provide outputs in shown in Table 3a, b & c. The authors provide example interpretations as sub-texts for Tables 3a, b, and c, but this reviewer feels that the authors should provide more information about their regression analysis in the methods section.

Response: We have revised our quantitative analyses completely to rigorously explore study outcomes,
 and to explain this better, have added the following text to the methods section: “Subsequent quantile regression analysis to estimate the median of the target value (instead of the mean), was used to explore the extent to which key input parameters were associated with key outcomes. Both univariable and multivariable quantile regression analyses were performed. Quantile regression was chosen over simple linear regression because our data was unlikely to meet assumptions required for linear regression. We included all categorical variables (country-level income, ART use, and TPT regimen given) in multivariate analyses and subsequently added combinations of continuous variables, as long as the number of studies with non-missing values was 10 or more, and there was no strong correlation (i.e. the correlation coefficient was not close to 1 or -1) between independent variables included in the model. To ensure a consistent sample size among final multivariable models, missing values for independent variables were imputed using medians.” (Page 7, Lines 171-183)

**C. Results**

(9) Line 146-148: In the abstract, the authors write "search identified 6,135 titles" (vs. 4,228 unique records in this line). It'd be important for the authors to be consistent in search results and selection process figures in the respective sections of the manuscript.

Response: We have changed this so that the results match the abstract – 6,615 titles were identified from the search.

(10) Line 156: For the rest of 30 studies, what methods did they use for the analysis and reporting of the economic evaluation of TPT in PLHIV? The authors break this down in Table 1 (modeling studies only), but for the text, it'd better to report as the following, for example: "50 studies (out of 57) used modeling methods to evaluate impact and/or cost-effectiveness TPT in PLHIV; 27 used decision analysis method and 23 used transmission modeling. Seven studies that did not use modeling used …(here, describe what type(s) of analysis was done for these studies that did not use modeling methods)."

Response: Thank you for this helpful suggestion. We have reworded as you have suggested (Page 8, Lines 205-209).

(11) Also to this reviewer, decision analysis models can also incorporate transmission model (as developed by one of the co-authors). As such, it may be better to describe these differences as modeling analysis that includes vs. excludes transmission or TB natural history models.

Response: We now use the terms “modelling study that considers TB transmission” and “modelling study that does not consider TB transmission” in order to describe types of modelling studies. This has been changed in Table 1, as well as in the text, where mentioned, to remain consistent.

(12) Analytic horizons: Can the authors classify studies by following categories?

*  No analytic horizons stated or less than 2 years
*  Analytic horizon between 2 and 10 years
*  Analytic horizon more than 10 years (for this, indicate in the sub-text maximum analytic horizon used)

Response: We have now classified studies according to these categories in Table 1. For those with analytic horizons more than 10 years, we have indicated in a footnote that the longest time horizon was 100 years. (Page 11, Line 226).
 
**Comments on Table 1**

(13) Population à it may be better to report each study's main population investigated and whether PLHIV was a sub-set or main population that was studied.

Response: We have now separated studies by whether population investigated was entirely made up of PLHIV, or if PLHIV were a subset. See Table 1 Study characteristics.

(14) It will be important to further classify studies if they did more detailed empiric costing or simple costing using reported unit cost estimates from other studies (or databases). Also for empiric costing, it'd be good to indicate if studies include costs associated with implementation process (e.g. Sohn H, Tucker A, Ferguson O, Gomes I, Dowdy D. Costing the implementation of public health interventions in resource-limited settings: a conceptual framework. Implement Sci [Internet]. 2020 Dec 29;15(1):86. Available from: <https://implementationscience.biomedcentral.com/articles/10.1186/s13012-020-01047-2>)

Response: We appreciate this suggestion. We have added a new section to Table 1 titled “Costing Details” where we elaborate on costing method (simple costing vs. empiric costing). Of those that undertook empiric costing or a combination of simple and empiric costing, 15 studies included costs associated with TPT implementation (Page 12, Lines 263-264). We also elaborate on the costing perspective used in studies that report cost or cost-effectiveness outcomes (Table 1).

(15) For studies that modeled or assessed LTBI tests (e.g. either TST or IGRA), were any of these studies compared impact/effectiveness/cost-effectiveness of TST vs. IGRA and how did studies used parameters for diagnostic accuracy for LTBI tests?

Response: Three studies compared the impact/effectiveness/cost-effectiveness of TST vs. IGRA; we have now indicated this in Table 1. These studies included parameters for diagnostic accuracy in models as a way to determine how many individuals would receive TPT. Diagnostic accuracy parameters were assessed in sensitivity analysis. In two studies [1,2]. TST sensitivity was found to be one of the more influential parameters in determining study outcomes (see S10a Table).
[1] <https://pubmed.ncbi.nlm.nih.gov/16776453/>

[2] <https://pubmed.ncbi.nlm.nih.gov/17609049/>

**Comments on Table S5**

(16) For probabilities, it'd be important to indicate of they are life time probabilities or time-associated probabilities.

Response: Thank you for this suggestion. We have now added a footnote to this effect: “The probability of an adverse event from TPT is a time-associated probability; as long as an individual is given TPT, there exists a probability of occurrence of an adverse event. The probabilities of progression and reactivation are also time-associated; when we refer to progression we mean “progression to active disease within the first 2-5 years of the analytic horizon”, and reference to reactivation means “progression to active disease after the first 5 years of the analytic horizon” under Table S5, Page 10.

(17) Cost of adverse event: It'd be good to indicate what were the most frequently cited adverse event and their associated probability (describe in the sub-title)

Response: We have also added a footnote to this effect under Table S5, Page 10: “Reflects the probability and costs of severe adverse event, which included management of hepatotoxicity (median probability of occurring = 0.005), acquired drug resistance (median probability of occurring = 0.010), or symptoms of rash, pruritus, gastrointestinal distress, and arthralgia (median probability of occurring = 0.045)”.

(18) Line 209-210 & 255: In the method section or directly next to the text in line 255, can authors provide rational for using $1,000 as a threshold (if possible, provide what would $1,000 per percent reduction in active TB incidence would measure against more traditional ICER estimate - e.g. cost per DALY averted/life years save, TB cases averted etc.)?

Response: We have changed the word “threshold” to “value” (i.e. the dashed line represents an incremental cost-effectiveness *value* of $1000), in the figure, Page 14 Lines 315-316. This line was intended to help visualize the distribution of data points on the cost-effectiveness plane. As well, we have changed this figure to illustrate the distribution of incremental cost **per active TB case averted**, instead of incremental cost per percent reduction in active TB incidence.

(19) Line 290-291: Was this due to increase in cost or reduced effectiveness?

Response: This was due to increase in cost. However, as you will note in our revised submission, we are no longer using ICERs in quantitative analyses due to difficulty of interpretation, as you have pointed out in this comment. Instead, we are using incremental net monetary benefit, which is easier to interpret.

**D.  Discussions (comments are specific to paragraphs)**

(20) Potential determinants of CE would also depend on what WTP is considered. These vary significantly from one setting to another. Therefore, I agree with the use of cautionary vocabulary of "potential" but it would be good to indicate under what threshold considerations this study considered these variables to be 'potentially' influencial in determining CE of TPT in PLHIV.

Response: Thank you for pointing this out. We have replaced our ICER outcomes with incremental net monetary benefit, which incorporates willingness to pay. We have indicated in our methods that we assume the willingness to pay threshold to be the study setting’s GDP per capita; Page 7, Line 171-172. We have also revised the text in the discussion to read as follows, in order to mention the potential influencers of cost-effectiveness: “We identified several potential determinants of cost-effectiveness in modeling studies, however only country-level income and consideration of ART use or cost remained associated with TPT cost-effectiveness in multivariable analysis.” Page 24, Line 463-466.

(21) Third paragraph: It's quite a lengthy discussion on heterogeneity. What is noted here is important and adequate, but other studies have already summarized these issues. What would be beneficial for the readers is to know what types of variabilities/heterogeneities are there across the studies on three key things: 1) input parameters (a. cost ; b. key epidemiologic and TB natural history parameters) 2) modeling strategy; and 3) outcome measures. This study has gone lengths to discover and summarize a lot of information from various studies that investigated impact/effectiveness and cost effectiveness of TPT in PLHIV, but did not adequately described differences across these studies in a systematic manner that could help with better alignment and standardization in evaluating effectiveness/impact and cost-effectiveness of TPT.

Response: We have elaborated on our results to summarize heterogeneity in input parameter values, which account for most of the heterogeneity. See response to Reviewer 1, Comment 1. By providing ranges of values of input parameters used in these studies, we illustrate the extent of heterogeneity. As well, in the discussion we explain that this heterogeneity made studies difficult to summarize and limited the extent of pooling through meta-analytical techniques; Page 25, Line 497-499. On the other hand, there wasn’t much heterogeneity in modelling methods and outcome measures, so we have not discussed this at greater length.

(22) Fourth paragraph: It is important for these modeling studies to separately and conjointly investigate the main drivers of costs, effectiveness, and cost-effectiveness. Many economic evaluation studies that investigate the cost-effectiveness solely focus on ICER estimate. The authors mention that provision of ART modify the effect of TPT among PLHIV. Contrastingly, provision of TPT enables positive health outcomes (i.e. people are surviving longer) which subsequently increases health systems costs. Without investigating these in more detail within the model and reporting these results, many of the future studies investigating the value of TPT will likely suffer the same issue of limited interpretation of the ICERs. Authors can help strengthen the contents of this paragraph by providing a bit more guidance on what are important analyses these modeling studies concerning TPT for PLHIV (or knowledge gaps that can improve overall modeling).

Response: Thank you for this suggestion. We have investigated some key results in more detail, in the third paragraph of our discussion (Page 24-25, Lines 477-486). We also state, later on in our discussion, what may improve on knowledge gaps among modelling studies. Namely, we mention the need for more studies on shorter, rifamycin based regimens: “In addition, shorter, rifamycin-based regimens are increasingly recommended for TPT worldwide, making it important to understand their potential costs, effectiveness, and cost-effectiveness, compared to other treatment options” (Page 26, Line 521-523). We also discuss key knowledge gaps, such as inconsistent reporting of input parameters and inconsistent reporting of outcome uncertainty. We refer to a modeller’s wishlist that underscores the importance of improved reporting among TB modelling studies in order to help fill knowledge gaps [1]. Page 25-26, Line 501-506.

[1] <https://pubmed.ncbi.nlm.nih.gov/23743307/>

(23) If the authors consider the fifth paragraph is the most important part of their study ('important finding'), it should be one of the first ones to come. I'd suggest a bit of restructuring of the paragraphs so that ordering of the contents be:

1)      Overall summary of the findings
2)      Most important finding of the study - TPT in PLHIV is cost-effective
3)      Discussion of other key findings (see further comment on this matter below)
4)      Discussion on strengths of the author's work
5)      Discussion on limitations of the authors work
6)      Conclusion

Response: Thank you for this helpful suggestion. We have rearranged our discussion accordingly.

**E.  Minor content and formatting issues**

(24) Line 38: There is an extra dot after the word "quality".

Response: We have removed this dot.

(25) Line 45: Effectiveness measured as what?

Response: Effectiveness measured as the extent of reduction in active TB incidence; we have added this detail. Page 2, Line 49.

(26) Line 46: Specify what downstream costs considered (e.g. post TPT health systems and/or patient costs?)

Response: Downstream costs considered are post TPT health systems costs. We have added this detail. Page 2, Line 50.

(27) Line 71: Perhaps revise to: "those who may be infected with and are at risk of"

Response: We have reworded as such. Page 4, Line 109.

(28) Line 74-75: Recommend revising the sentence to "Uptake of TPT has increased substantially in recent years, with close to two-fold (1.8 million to 3.6 million between 2018 and 2019) increase in TPT initiation amongst PLHIV."

Response: We have reworded as such. Page 4, Lines 112-114.

(29) Line 75: Can authors provide reference or comment on whether this 50% TPT coverage in PLHIV represent

Response: This referred to the extent of coverage of TPT in PLHIV; since revising our statement as suggested above, this point has been removed.

(30) Line 92: Please replace "excluded" to "ruled-out"

Response: We have reworded as such. Page 5, Line 133.

(31) Line 93-94: Please revise the sentence "study's projected outcomes…" to "study investigated costs, cost-effectiveness (assessed for programmatic yields such as case detection or extended to utility outcomes such as DALYs or QALYs), and epidemiologic impact estimates such as TB incidence, deaths."

Response: We have reworded as such. Page 5, Line 133-136.

(32) Table 2a & b: Would be better to switch the order of the columns (suggestion: switch the position of the "key outcome reported" with "Comparison Made" so that "Key outcome reported" is next to "Outcome value"). This would better help with interpretation of the units of outcome value.

Response: We have switched around the columns. Notably, these tables have been moved to the Supplementary Appendix as Tables S7a and S7b on Pages 16 and 18.

**Reviewer #2:**

**SUMMARY**

This well-written manuscript summarizes cost-effectiveness and modelling research that has investigated the consequences of preventive treatment of LTBI among individuals with HIV. I think the subject being summarized is worth addressing, but I have some methodological concerns, particularly regarding the regression analysis applied to the cost-effectiveness results.

Response: Thank you for your comments. We have addressed each of your points below.

**MAJOR ISSUES**

(1) Page 4, search strategy: I think a critical inclusion criterion would be that the study represented a comparison of two populations/cohorts where the ONLY difference was the receipt vs. non-receipt of IPT by HIV-positive individuals. This is implicit in the title and introduction of the paper, but I don't see this described explicitly at the top of the methods.

Response: We have added the following text: “study outcomes were disaggregated by receipt of TPT”. Page 5, Line 137. We did not specify that the only difference between study cohorts should be the receipt vs. non-receipt of TPT by PLHIV for two reasons: (1) in some studies, cohorts received different TPT regimens; and (2) in some studies, one cohort received an LTBI test (TST or IGRA) to determine who would receive TPT, while the other cohort did not receive this test (so all received TPT). We did not include either of these types of studies’ results in our quantitative analyses, which explored the receipt vs. non-receipt of TPT, but we did include these studies in more general descriptive analyses.

(2) A more specific concern regarding inclusion/exclusion criteria: I expect some studies described the results of IPT interventions where there is an initial screen for TB disease, and where this screen does not occur in the 'no-IPT' branch. Therefore, the CEA results would represent the costs/benefits of IPT, plus the costs/benefits of TB treatment triggered by the TB disease screening. The first inclusion criterion ('study population included at least a subset of PLHIV in whom active TB had been excluded') could be interpreted as ruling out studies that looked at this typical IPT strategy, but I am not sure if this is what was actually done. If both types of studies were included (ie studies that considered the costs and benefits of TB disease identified by the initial screen), then it would be good to include this as a stratification in the subsequent results.

Response: Indeed, six studies included the costs and benefits of TB disease identified by an initial screen [1-6]. Each of these, however, either included the costs and benefits of active TB screening in both TPT and no TPT study arms, or considered active TB screening as a separate intervention altogether. In all of these studies, we were able to identify the costs and effects of TPT, distinct from the costs and impact of active TB screening. As such, we have not further stratified our results to consider these studies separately.

[1] <https://www.thelancet.com/journals/langlo/article/PIIS2214-109X(18)30022-6/fulltext>

[2] <https://pubmed.ncbi.nlm.nih.gov/19460246/#:~:text=Results%3A%20Adherence%20to%20IPT%20at,through%20IPT%20was%20US%24955>.

[3] <https://pubmed.ncbi.nlm.nih.gov/21148723/>

[4] <https://journals.plos.org/plosone/article?id=10.1371/journal.pone.0030457>

[5] <https://pubmed.ncbi.nlm.nih.gov/16878226/>

[6] <https://www.ncbi.nlm.nih.gov/pmc/articles/PMC4886505/>

(3) The study seems to include papers that compare 'IPT' vs 'no-IPT' scenarios, as well as 'IPT for all' vs. 'IPT only for TST/IGRA positive'. These are quite different comparisons - I think it is fine for them to be considered in the same paper, but these results should not be included in the same quantitative analysis (as in Figure 3?), and care should be taken so that readers do not conflate them.

Response: In fact, we did not combine these two scenarios in our analyses. However, this reviewer’s comments made us realize that we were not clear enough about this. Therefore we have revised the title of all figures to clarify that the only scenarios included are those that compare TPT to no TPT.

(4) Page 6 lines 131: I have concerns about the approach taken to conduct quantitative analyses of the ICER results. By confirming that the ICER denominators are strictly positive (ie IPT strategy always has positive health benefits vs. no-IPT) the authors avoid the stickiest scenario, but this doesn't resolve the issue that the outcome (ICER) is inversely related to the denominator, so very small denominators could lead to very large ICERs. Bottom-coding the ICERs at zero also makes the interpretation of the regression results more difficult (as the denominators are strictly positive, the interpretation of these ICERs as cost-savings seems pretty straightforward), and it is unclear why the arithmetic means that are being calculated through the regression are meaningful quantities. Several of these concerns go away if one considers the median (since not outliers don't matter), and so median regression might represent a more defensible approach. Additional discussion of the difficulties of systematic reviews of cost-effectiveness results are found here (Shields and Elvidge Systematic Reviews 2020 <https://systematicreviewsjournal.biomedcentral.com/articles/10.1186/s13643-020-01536-x>). On net, I believe the methodological shortcomings one needs to accept to include this kind of analysis are not worth it.  I don't think this represents mistakes made by the investigators in implementing the approach, but the problematic conceptual justification of such comparisons.

Response: We have since switched to quantitative analyses of incremental net monetary benefit (INMB). We calculated INMB for each study that reported an incremental cost per active TB case averted comparing PLHIV given and not given TPT. We used the study setting’s GDP per capita as their willingness-to-pay threshold for these calculations. In addition, we have switched to conducting quantile regression (for the median) in lieu of simple linear regression. See also response to Reviewer 1 Comment 8. We have explained our regression methodology on Page 7, Line 175-184.
 
(5) As a separate concern about the regression analysis, I am not sure why variable selection is performed. If a variable is of interest I would think it would be included, and predictive performance isn't really an objective here.

Response: Variable selection for our regression analysis has been raised by all three reviewers. Pease see our consolidated response - Reviewer 1, Comment 8.

(6) The consideration of costing perspective seems insufficient. Whether the costs of averted TB disease care are included, and whether the costs of additional HIV care are included (a result of improved survival), are both factors that could have a meaningful impact on the ICER, yet these distinctions do not appear in the paper (unless I missed them!).

Response: Thank you for pointing this out. We have now added a section to Table 1 that elaborates on costing methodology, as well as costing perspective (e.g. health system, society, patient, etc.) Page 9.

(7) Page 13, lines 209: it is stated that 'In all studies, TPT was found to be cost-effective compared to no TPT, even with diverse willingness-to-pay thresholds specific to each study setting'. I don't see where this statement is supported in the results. For something to be cost-effective there needs to be either comparison to an explicit CE threshold, OR direct comparison to other interventions being considered for funding, in addition to a budget limit. Is this a result based on the conclusions drawn by the authors in each paper?  If so it would be good to state this. If it is a conclusion drawn based on the extracted ICERs this needs to be done carefully, noting that the historical approach of comparing to 1x and 3x per capita GDP is now increasingly questioned (as an example see Woods et al Value in Health 2016 <https://pubmed.ncbi.nlm.nih.gov/27987642/>).

Response: Indeed, this was based on the conclusions of individual studies, and not based on our own analyses. We have clarified this in the results on Page 13=14, Line 292-295, and in the discussion on Page 24, Line 461-462.

**MINOR ISSUES**

(8) Page 5, line 120: there is a list of values extracted from the papers, termed 'input parameters'. These are not all input parameters for the models, so perhaps a more general description can be used. In this list, it is unclear what 'consideration of ART use' means.

Response: We have revised the text to say “key model parameters” instead of “key input parameters”. Consideration of ART use meant that the study included an input parameter for ART efficacy or considered cost of ART in their model; we have added this description to help clarify what ART use means.

(9) Page 11, Table 2a: It is unclear what the 'Outcome Value' is.

Response: We have revised this to say “Value of Key Outcome Reported”. Notably, Tables 2a and 2b have moved to the Supplementary Appendix as Tables S7a and S7b, Page 16 and 18.

 (10) Page 25, lines 333-334: would be important to cite the work that has compared the parameterization and structure of models for LTBI (Ragonnet et al Epidemics 2017; <https://www.ncbi.nlm.nih.gov/pmc/articles/PMC6070419/> Menzies et al Lancet Infect Dis 2018 <https://pubmed.ncbi.nlm.nih.gov/29653698/>; Sumner and White BMC Infect Dis 2020 <https://pubmed.ncbi.nlm.nih.gov/33228580/>)

Response: Thank you for this suggestion! We have added those references to Page 25, Line 500.

**Reviewer #3:**

This paper reports a systematic review of studies of tuberculosis preventive therapy in people living with HIV. I was asked for a statistical report and I interpret that to include all aspects of the design and conduct of the study. I note that this is one of a collection of three papers and I have previously reviewed the other two.

Response: Thank you for your comments. We have responded to each of your points below.

**Points of detail**

(1) Page 6 If the authors did wish to weight their meta–analysis then one possible choice of weights would be some function of the sample size. I think technically they have done a meta–regression but not the usual inverse–variance weighted one but I think their description is fine.

Response: We have now employed inverse-variance weights in our meta-analysis, and have added a description accordingly to our methods: “We also performed meta-analysis on our two key outcomes. As there is no universal method to meta-analyze results of modelling studies, we referred to other publications methodology, which include estimating standard error from probabilistic sensitivity analyses or one-way analyses if PSA was not done. We pooled studies conservatively using an inverse variance method, with a Sidik-Jonkman estimator for tau and Hartung-Knapp adjustment for our random effects model. We pooled studies conducted in high income countries and low- and middle-income countries separately and where possible, further stratified on key input parameters.” Page 7, Line 185-191.

(2) Page 6 Selecting a subset of variables in a way driven by the data leads to a model which is unlikely to replicate (Babyak, 2004). Screening variables to enter the model has been criticised too (Sun et al., 1996) In fact the results presented in Table S9 do not match my understanding of what the authors state here in the methods as there they seem to have included all the categorical covariates and then added each of four continuous ones alone. If that is what was done it seems to me more defensible than a full forward selection approach.

Response: Variable selection for our regression analysis has been raised by all three reviewers. Pease see our consolidated response - Reviewer 1, Comment 8.

(3) Table 3c Why are there seven continuous variables here but only five in 3a and 3b?

Response: Indeed, because studies with missing values could not included be included in these multivariable models we were forced to reduce the number of continuous variables considered in Tables 3a and 3b, compared to 3c. However, to simplify our presentation of results Table 3c has been removed; we now only focus on two outcomes: percent reduction in active TB incidence and incremental net monetary benefit. We decided to drop the analysis of mortality because of the limited number of studies eligible for inclusion, and to focus on only one effectiveness outcome (active TB).

**Figures**

(4) I think it might be better for Figures 2, 3 and 4 to use unfilled symbols as it is hard to tell whether over–printing is spoiling the visual impact. An alternative would be adding some horizontal jitter. The same applies to Figure S5.

Response: Thank you for this suggestion. We chose to add horizontal jitter to these figures.

(5) Some of the continuous predictors seem to me to suggest that they are ecological in nature. For instance I assume that adherence means being enrolled in a study with that level of average adherence, not personally adhering. If that is correct then perhaps a mention in limitations might be in order.

Response: We have added the following to our limitations discussion: “Individual level adherence to TPT is likely to vary within a population, but model parameterization did not allow us to investigate this factor” Page 26, Lines 512-513.

**Point of more substance**

(6) One possible explanation of the very convincing conclusion that all studies report the same effect, at least qualitatively, is that anyone who found the converse would struggle to find an outlet for it. Are the authors convinced that their search strategy can rule this out? There is no mention of an explicit language restriction but the choice of bibliographic databases has a bias towards English language publications (Pilkington et al., 2005).

Response: We have added the following text to our discussion to this effect: “Another limitation could be publication bias towards positive effects of TPT, as models showing a lack of benefit may not be published. Individual level adherence to TPT is likely to vary among a population, but model parameterization did not allow us to investigate this factor. Finally, our bibliographic databases favor English publications, which may introduce bias.” Page 26, Lines 510-514.

**Summary**

No major concerns.

Michael Dewey

References

1. M A Babyak. What you see may not be what you get: a brief, nontechnical introduction to overfitting in regression–type models. Psychosomatic Medicine, 66:411–421, 2004.
2. K Pilkington, A Boshnakova, M Clarke, and J Richardson. ‘No language restrictions’ in database searches: what does this really mean? The Journal of Alternative and Complementary Medicine, 11:205–207, 2005.
3. G-W Sun, T L Shook, and G L Kay. Inappropriate use of bivariable analysis to screen risk factors for use in multivariable analysis. Journal of Clinical Epidemiology, 49:907–916, 1996.
