# Supplementary material for: Economic and modeling evidence for tuberculosis preventive therapy among people living with HIV: A systematic review and meta-analysis
Source: PLoS Med. 2021 Sep 14;18(9):e1003712. doi: 10.1371/journal.pmed.1003712 (PMC8439468; doi:10.1371/journal.pmed.1003712)
Supplement: Attachment — Submitted filename: CostEffectiveness TPTinHIV Response to Reviewers_R2.docx [file pmed.1003712.s006.docx]

Dear Dr Turner,

Thank you for your letter of June 10, 2021. We appreciate the opportunity to address remaining comments and suggestions and have revised the attached manuscript to address these. In the point-by-point response below, we have provided exact locations in our manuscript (page and line number) where revised text may be found.

We hope this revised version is now acceptable for publication.

Aashna Uppal and Dick Menzies, on behalf of all authors.

**Requests from Editors:**

1) Throughout the paper, please modify the text to soften language such as "was effective ...", which at PLOS Medicine we generally reserve for evidence from randomized studies.

We have added “potentially” or “predicted to be” to all statements like that, so that it reads “was potentially effective”, or “was predicted to be cost-effective”.

2) At line 48, please amend the wording to "... was predicted to be effective at averting TB disease." or similar, given the study designs included.

We have reworded as such.

3) At line 51, please amend the text to "... were estimated to be less than $1500 ...".

We have reworded as such.

4) Please restructure the author summary so that each of the three subsections contains 2-4 points. For example, the number of studies (61) does not need a specific point and can be included in another.

We have combined some points under the “What did the researchers do and find?” subsection; it now contains 4 points instead of 6.

5) Please use the style "... 2 studies" throughout the text, although numbers should be spelt out at the start of sentences (as at present).

We have updated the text to use this style of depicting numbers throughout.

6) Please quote p values alongside 95% CI, where available.

We have added p values to Tables 4 and S14. Where the p value was greater than or equal to 0.001, we stated the exact p value. Where it was less than 0.001, we wrote “<0.001”. We have also quoted p values beside 95% CIs in the text where required. Notably, we were not able to add p values to Tables 2, 3, S12, and S13 (univariable and multivariable analyses). This is because we used the rank method to estimate standard error in our quantile regression models; this method is recommended for use unless there are specific requirements, like a sample size >1000, for example. One limitation of this rank method is that it cannot generate p values, nor can it generate parameters that allow one to back calculate p values. As such, we are limited to confidence intervals only for these analyses. We have drawn statements of “significance” from these confidence intervals. To make this clear, we have added this sentence on Page 16 Line 382:

“Importantly, we define a parameter to be significant if its confidence interval does not cross the null (0).”

7) Please remove the information on funding from the end of the main text. In the event of publication, this information will appear in the article metadata, via entries in the submission form.

We have removed the funding information from the end of the main text.

8) Please make that "-$74 to $66" in table 3b.

Thank you for noting this; we have added the dollar sign to $66.

9) We generally ask that exact p values are quoted, or "p<0.001"; and note that "p<0.01" appears in fig. 5.

Where the p value was greater than or equal to 0.001, we have now stated the exact p value. Where it was less than 0.001, we wrote “<0.001”.

10) Please ensure that appropriate journal name abbreviations are used, e.g., "Proc Natl Acad Sci U S A" for reference 2 and others; and "PLoS ONE".

Changes were made to journal name abbreviations accordingly for reference 2, 20, 29, 35, 47, 54, 71, 78 and 80.

11) Noting reference 12 and others, please list a maximum of 6 author names, followed by "et al.".

Changes were made to authors lists accordingly for reference 2, 7, 12, 17, 19, 24, 26, 28, 33, 36, 41, 49, 54, 58, 61, 62, 66, 69, 70, 74, 81, and 86.

12) Noting reference 37, please ensure that all references have full access information.

We have ensured all references have full access information; more information was added to references 25 and 37.

13) Please rename the attached checklist "S1_PRISMA_Checklist" or similar and refer to it by this label around line 125.

We have referred to the checklist as S1_PRISMA_Checklist in Line 124, and have renamed the attached checklist as such.

**Comments from Reviewers:**

*** Reviewer #1:

Authors of the manuscript have adequately address all of the issues raised in my review. Thank you for this important work!

*** Reviewer #2:

These revisions largely resolve my original concerns. I just have some minor issues with the NMB analysis.

1) For the analysis of NMB, there is great uncertainty as to what the correct threshold is for a given country. For this reason it would be helpful to do a sensitivity analysis with alternative values (eg 0.5x and 3x per capita GDP), which could go in the appendix.
   a. Thank you for this helpful suggestion. We have explored univariable, multivariable, and pooling analyses using 0.5x and 3x per-capita GDP as thresholds for calculating incremental net monetary benefit. We have stated in the method section on Page 7 Line 175: “We assumed a willingness-to-pay threshold of country-level gross domestic product (GDP) per capita, and explored this assumption’s effects with sensitivity analyses.” We have also stated in the results section Page 24 Line 466: “Repeating pooling analyses with a lower (0.5x GDP) and higher (3x GDP) willingness-to-pay threshold demonstrated that incremental net monetary benefit is proportional to scale; as GDP increased or decreased, the pooled incremental net monetary benefit also increased or decreased. Similarly, repeating univariable and multivariable analyses with a lower and higher willingness-to-pay threshold did not change any conclusions; variables that were significantly associated with incremental net monetary benefit tended to remain significantly associated (see S12-S14 Tables)”.
2) Secondly, the text that describes the NMB calculation needs to be clearer. What was multiplied by per capita GDP to value the health effects? Conventionally this would be DALYs (at least in high-burden settings), and this is the outcome for which conventions around defining WTP as some multiple of per capita GDP are based. Also, the text should state that these NMB is calculated using the per-person costs and DALYs -- while the ICER in unchanged by scale, NMB is proportional to scale, so need to specify this. Also useful to provide some citation for NMB (eg the Stinnett paper <https://pubmed.ncbi.nlm.nih.gov/9566468/>).
   a. We have now clarified on Page 7 Line 171-180 as follows: “We calculated incremental net monetary benefit as: Difference in Effectiveness*Willingness to Pay – Difference in Cost. Conventionally, DALYs are used to complete the net monetary benefit calculation, however, we considered relative reduction in active TB incidence as a measure of effectiveness instead because only 6 studies reported DALYs. We assumed a willingness-to-pay threshold of country-level gross domestic product (GDP) per capita, and explored this assumption’s effects with sensitivity analyses. Net monetary benefit was chosen over ICERs because of ease of interpretation (i.e. the magnitude of negative ICERs does not convey useful information) [Stinnett 1998] and proportionality to scale vis-à-vis the willingness-to-pay threshold.”

*** Reviewer #3:

The authors have addressed my points.

Michael Dewey

***
