# Supplementary material for: Economic and modeling evidence for tuberculosis preventive therapy among people living with HIV: A systematic review and meta-analysis
Source: PLoS Med. 2021 Sep 14;18(9):e1003712. doi: 10.1371/journal.pmed.1003712 (PMC8439468; doi:10.1371/journal.pmed.1003712)
Supplement: Attachment — Submitted filename: CostEffectiveness TPTinHIV Response to Reviewers_R4.docx [file pmed.1003712.s007.docx]

Dear Dr Turner,

Thank you for your letter of June 20, 2021. We appreciate the opportunity to address final comments and have revised the attached manuscript to address these. In the point-by-point response below, we have provided exact locations in our manuscript (line number) where revised text may be found.

We hope this revised version is now acceptable for publication.

Aashna Uppal and Dick Menzies, on behalf of all authors.

**Requests from Editors:**

1) Please check that numbers are quoted consistently throughout the ms: we note that $271 at line 58 appears to be quoted as $270 at line 441.

We changed line 441 to read $271. Apologies for that. We have carefully checked that numbers are quoted consistently throughout.

2) Where p values are quoted in table 4, please also quote these numbers alongside the relevant 95% CI in the abstract, e.g., at line 58.

We have added p values to the abstract where Table 4 results are quoted. In order to reduce the word count to less than 500 after this addition, we adjusted wording of lines 40-41 and 65 slightly.

3) We feel that more cautious language is needed in the abstract, and at the corresponding points in the main text. We note "... found TPT to be more effective" at line 56 and similar wording at line 59. We ask you to convert the phrasing to "... TPT appeared to be more effective" or similar (we did not see statistical tests supporting the arguments of "more effective", and the 95% CI appear to overlap).

We have changed the phrasing to “suggested that TPT may be more effective” and “TPT appeared to be more effective” in lines 56 and 59.

4) At line 84, please add the missing "at" and again adopt more cautious language regarding effectiveness.

We have added the missing “at” and have changed the language to “TPT appeared to be more effective” in line 84.

5) At line 489, again we suggest "apparent effectiveness" or similar.

The wording has been changed to “TPT’s apparent effectiveness” in line 489.
